# Controlling Intramolecular Interactions in the Design of Selective, High-Affinity Ligands for the CREBBP Bromodomain

**DOI:** 10.1021/acs.jmedchem.1c00348

**Published:** 2021-07-13

**Authors:** Michael Brand, James Clayton, Mustafa Moroglu, Matthias Schiedel, Sarah Picaud, Joseph P. Bluck, Anna Skwarska, Hannah Bolland, Anthony K. N. Chan, Corentine M. C. Laurin, Amy R. Scorah, Larissa See, Timothy P. C. Rooney, Katrina H. Andrews, Oleg Fedorov, Gabriella Perell, Prakriti Kalra, Kayla B. Vinh, Wilian A. Cortopassi, Pascal Heitel, Kirsten E. Christensen, Richard I. Cooper, Robert S. Paton, William C. K. Pomerantz, Philip C. Biggin, Ester M. Hammond, Panagis Filippakopoulos, Stuart J. Conway

**Affiliations:** † Department of Chemistry, Chemistry Research Laboratory, University of Oxford, Mansfield Road, Oxford OX1 3TA, U.K.; ‡ Nuffield Department of Clinical Medicine, Structural Genomics Consortium, University of Oxford, Old Road Campus Research Building, Roosevelt Drive, Oxford OX3 3TA, U.K.; § Department of Biochemistry, University of Oxford, South Parks Road, Oxford OX1 3QU, U.K.; ∥ Oxford Institute for Radiation Oncology, Department of Oncology, University of Oxford, Oxford OX3 7DQ, U.K.; # Department of Chemistry, University of Minnesota, 207 Pleasant Street SE, Minneapolis, Minnesota 55455, United States; ∇ Department of Chemistry, Colorado State University, 1301 Center Ave, Ft. Collins, Colorado 80523-1872, United States

## Abstract

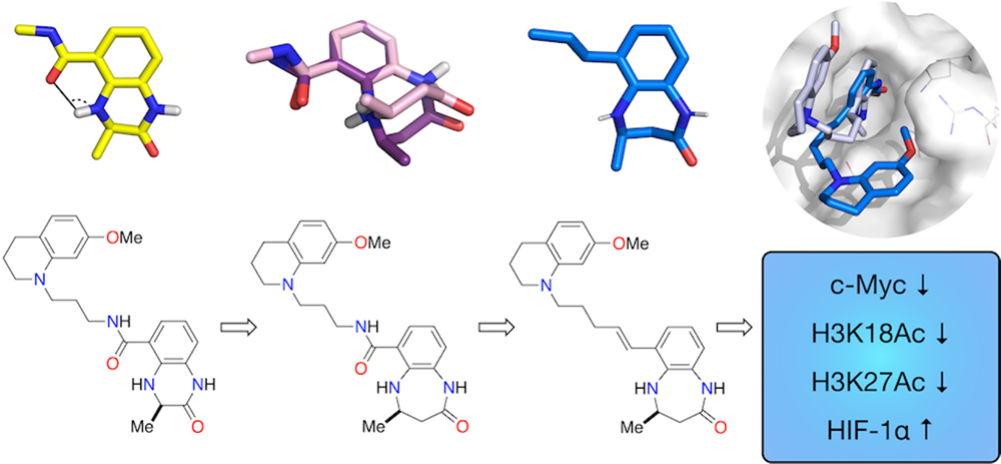

CREBBP (CBP/KAT3A) and its paralogue EP300 (KAT3B) are lysine acetyltransferases (KATs) that are essential for human development. They each comprise 10 domains through which they interact with >400 proteins, making them important transcriptional co-activators and key nodes in the human protein–protein interactome. The bromodomains of CREBBP and EP300 enable the binding of acetylated lysine residues from histones and a number of other important proteins, including p53, p73, E2F, and GATA1. Here, we report a work to develop a high-affinity, small-molecule ligand for the CREBBP and EP300 bromodomains [(−)-OXFBD05] that shows >100-fold selectivity over a representative member of the BET bromodomains, BRD4(1). Cellular studies using this ligand demonstrate that the inhibition of the CREBBP/EP300 bromodomain in HCT116 colon cancer cells results in lowered levels of c-Myc and a reduction in H3K18 and H3K27 acetylation. In hypoxia (<0.1% O_2_), the inhibition of the CREBBP/EP300 bromodomain results in the enhanced stabilization of HIF-1α.

## Introduction

CREBBP (also CBP or KAT3A) and its paralogue EP300 (also KAT3B) are lysine acetyltransferases (KATs)^1,2^ that are essential for normal human development. Somatic mutations in CREBBP and EP300 are associated with a range of cancers^3^ germline CREBBP mutations and are linked with Rubinstein-Taybi syndrome (RTS).^4^ This syndrome is characterized by growth impairment, learning difficulties, and distinctive facial and skeletal anomalies.^5^ RTS patients also have an increased likelihood of developing some forms of cancer. That germline mutations in CREBBP, but rarely those in EP300, result in RTS demonstrates the non-redundancy of these two proteins, an observation that is supported by experiments on embryo development in mice.^6^

**Figure 1:**
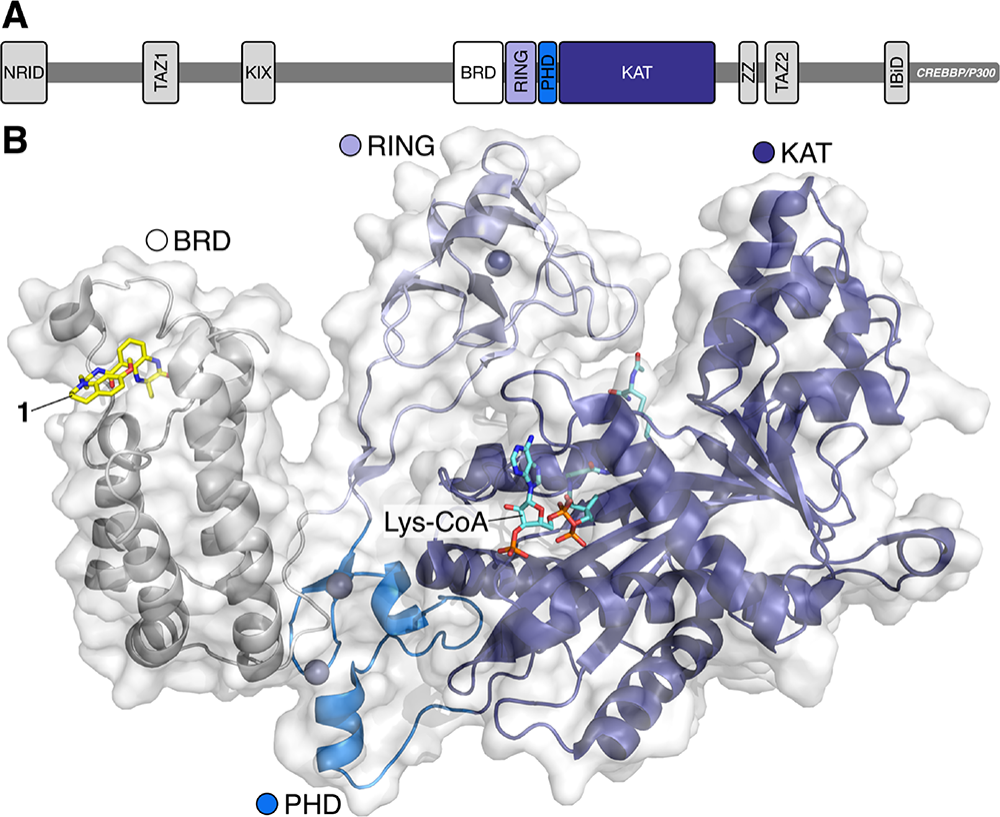
(A) Domain structure of CREBBP and EP300, which comprise 10 regions: NRID (N-terminal nuclear receptor interaction domain); TAZ1 (transcription adaptor zinc finger 1); KIX (kinase inducible); BRD (bromodomain); RING (really interesting new gene); KAT/HAT (lysine/histone acetyltransferase; ZZ (zinc finger); TAZ2 (transcription adaptor zinc finger 1); IBiD (interferon binding domain).^9,10^ (B) An X-ray crystal structure of the BRD, RING, PHD, and KAT domains of EP300 bound to Lys-CoA (carbon = aquamarine) obtained by Delvecchio *et al.*(10) overlaid with an X-ray crystal structure of compound **1** (carbon = yellow) bound to the CREBBP bromodomain (PDB code: 4NYX).^11^

CREBBP and EP300 comprise 10 domains each: NRID, TAZ1, KIX, bromodomain, RING, PHD, KAT, ZZ, TAZ2, and IBiD (Figure 1) through which they interact with over 400 different proteins. These interactions make them important transcriptional co-activators and key nodes in the human protein–protein interactome.^5^ CREBBP and EP300 are both capable of acetylating lysine residues on all four histones, although they show differing selectivities for which lysine residues they target.^7^ While the histone-targeted KAT activity of CREBBP/EP300 has been heavily studied, they also acetylate thousands of other proteins,^8^ including p53, p73, E2F, and GATA1,^3^ meaning that CREBBP/EP300 are involved in multiple signaling pathways. Despite the advances made in our understanding of CREBBP/EP300 KAT function, less is known about the specific role of the other protein domains.^9^

This has prompted work to make small-molecule probes for these domains, including the KIX,^12^ TAZ1,^13^ NRID,^14^ and bromodomain,^11,15−25^ in addition to the KAT domain.^25,26^ These molecules are starting to allow dissection of the specific role of a given domain within the context of the whole protein function.

Over the last decade, bromodomains have emerged as exciting targets in medicinal chemistry.^27−32^ While work has focused on the development of ligands for the BET bromodomains,^33^ especially BRD4, as a result of their role in a number of cancers,^34^ more recent work has seen the development of high-affinity ligands for non-BET bromodomains.^35−38^ Building on a pioneering work by Zhou, who reported the first ligands for the CREBBP bromodomain,^39^ we reported the first high-affinity ligands for the CREBBP/EP300 bromodomain.^11^ This study identified the key interactions required for binding to the CREBBP bromodomain, and these findings have subsequently underpinned the development of a number of other CREBBP bromodomain ligands.^40^ One challenge in the development of CREBBP bromodomain ligands for use as probes is ensuring that they have sufficient selectivity over other bromodomains. This is to enable firm conclusions to be drawn from data generated using the probes in cells and more intact systems. While there are some differences between the CREBBP bromodomain and those found in the BET proteins,^11,27,30,41^ the presence of the LPF shelf (CREBBP) and WPF shelf (BET) regions has made developing probes with high selectivity challenging. For example, SGC-CBP30, one of the first high-affinity CREBBP/EP300 bromodomain ligands retains some activity against BRD4,^15^ although more selective ligands have subsequently been developed (Figure S1).

The use of these ligands has demonstrated that the CREBBP/EP300 bromodomain is an important therapeutic target in castration-resistant prostate cancer^42,43^ and diffuse large B cell lymphoma.^44^ In addition, the CREBBP bromodomain ligand CCS1477 is currently in clinical trials to evaluate its effects in the treatment of acute myeloid leukemia and multiple myeloma (NCT04068597). Here, we report ligand development and optimization building on our initial series of compounds, resulting in a high affinity (ITC *K*_d_ = 102 ± 10 nM) CREBBP bromodomain ligand, (−)-OXFBD05 (**2**). This compound binds selectively to the CREBBP and EP300 bromodomains, is >100-fold selective over the first of the tandem BRD4 bromodomains, BRD4(1), and shows no binding to a phylogenetically diverse panel of 10 bromodomains at a concentration of 1 μM. The enantiomeric companion compound, (+)-OXFBD05 (**3**), shows no binding to the CREBBP and EP300 bromodomains and does not bind to the same panel of 10 bromodomains, making it a useful companion compound. Studies in HCT116 colon cancer cells demonstrate that the inhibition of the CREBBP/EP300 bromodomain results in the downregulation of c-Myc, which is consistent with the hypothesis that (−)-OXFBD05 (**2**) is selectively inhibiting the CREBBP/EP300 bromodomains in this cell line. A modest but repeatable reduction in H3K18 acetylation is observed, demonstrating that the bromodomain plays a role in the KAT function of CREBBP/EP300. In hypoxia, the stabilization of HIF-1α above the level observed in hypoxia alone was observed. In contrast, the inactive enantiomer, (+)-OXFBD05 (**3**), shows none of these effects in the same cell line.

## Results and Discussion

**Figure 2:**
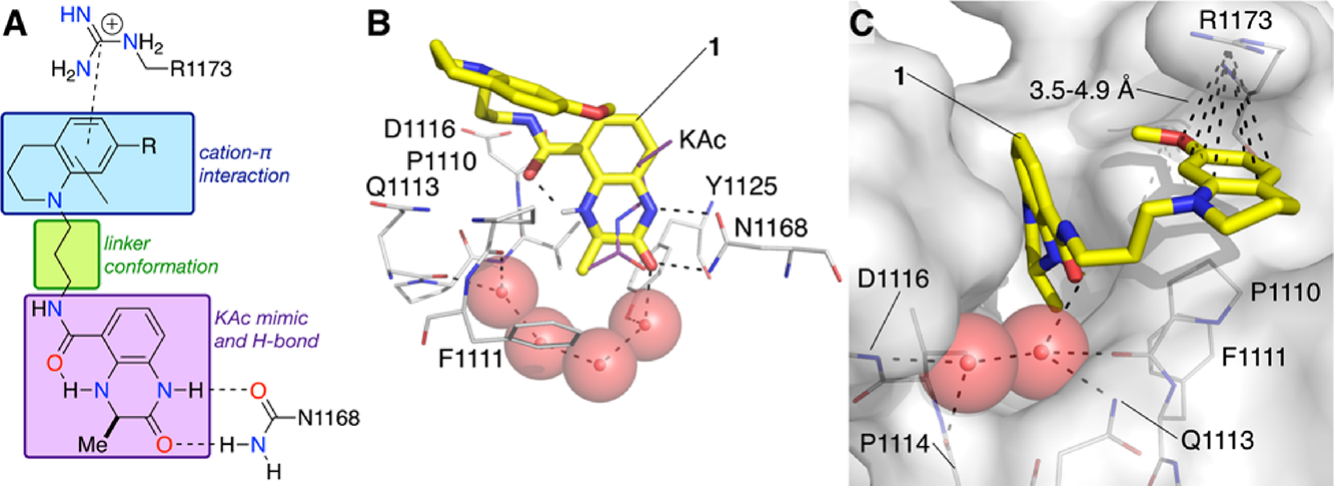
(A) The three regions of compounds **1** (R = OMe) and **4** (R = H). (B) X-ray crystal structure of compound **1** (carbon = yellow) bound to the CREBBP bromodomain (PDB code: 4NYX). The dihydroquinoxalinone headgroup binds to the KAc pocket forming hydrogen bonding interactions with N1168. (C) The electron-rich 7-(methoxy)tetrahydroquinoline group of **1** forms cation−π interactions with the positively charged R1173.^11^

Compound **1** proved to be a powerful tool for determining the structural requirements for small molecule binding to the CREBBP bromodomain; however, it retained low micromolar affinity for the BET bromodomains.^11^ The strong BET phenotype means that ligands for other bromodomains need to be highly selective for their target bromodomain over the BET family to be useful in cellular studies. Therefore, we wanted to develop compounds that show at least 100-fold selectivity for CREBBP/EP300 over other bromodomain targets, while retaining high CREBBP bromodomain affinity. To this end, we investigated the structure–activity relationships (SAR) of compound **1**. This compound (**1**) comprises three key sections, the KAc mimic, a tetrahydroquinoline (THQ) group that interacts with R1173 and the hydrophobic leucine, proline, and phenylalanine (LPF) shelf, and a linker that joins these two motifs (Figure 2). Here, we describe work to alter the 6-membered 3,4-dihydroquinoxalinone ring to the 4,5-dihydrobenzodiazepinone ring, understand the effects of this change on a key intramolecular hydrogen bond, and determine the structural features that affect the CREBBP/EP300 bromodomain selectivity for this class of ligands.

### Optimization of the Acetyl-Lysine Mimic

**Scheme 1:**
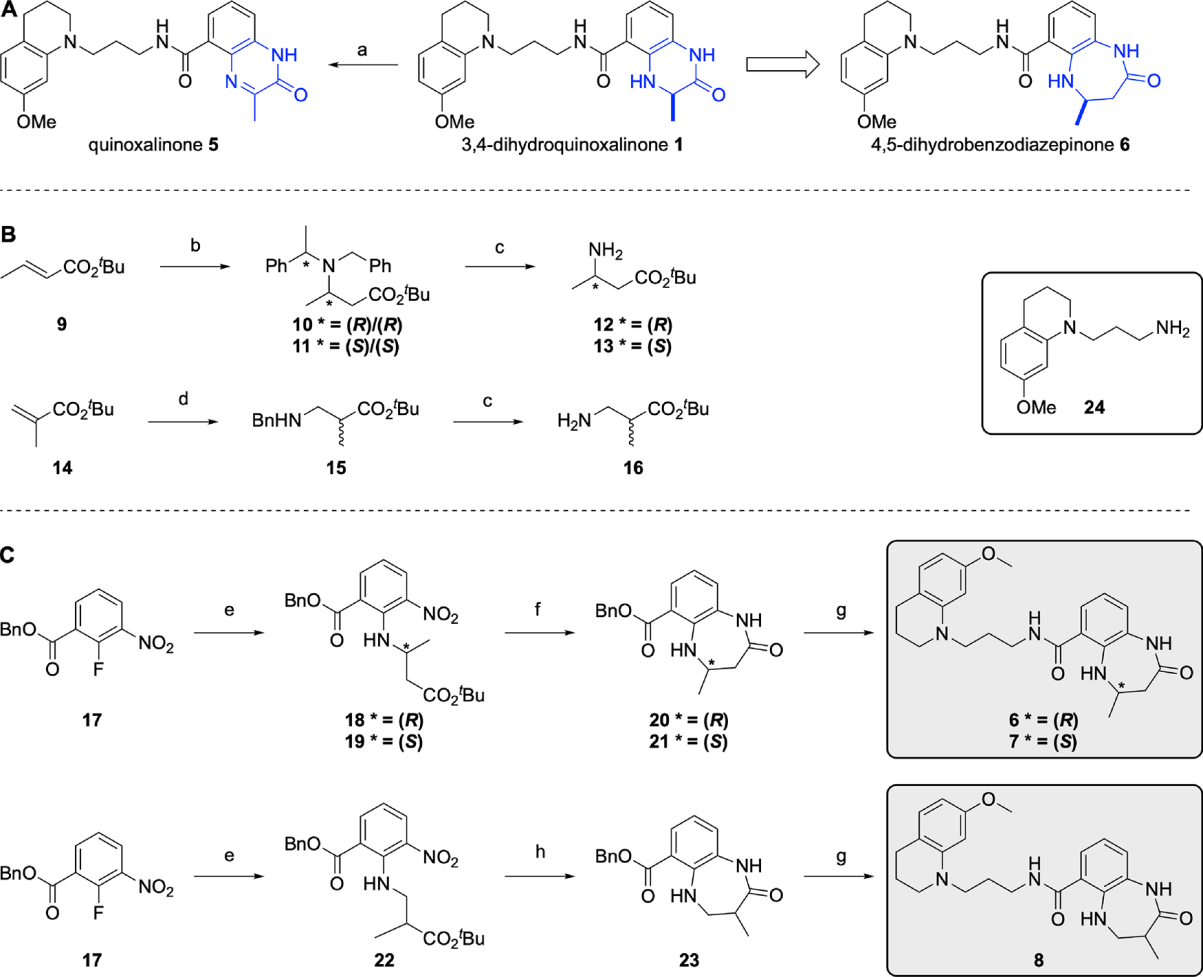
(A) Oxidation of the 3,4-Dihydroquinoxalinone Headgroup (**1**) and Ring Expansion to Give the 4,5-Dihydrobenzodiazepinone KAc Mimic (**6**); Reagents and Conditions: (a) DMSO, 50 °C, 5 days 14% conversion; (B) Synthesis of β-Amino Acid Ester Derivatives **12**, **13**, and **16**; Reagents and Conditions: (b) *N*-Benzyl-1-phenylethanamine, *^n^*BuLi, THF, −78 °C, then **9**, THF, −78 °C, 83%; (c) H_2_, Pd(OH)_2_/C, MeOH/H_2_O, AcOH, 85%; (d) BnNH_2_, DBU, 90 °C, 16 h, 51%; (C) Synthesis of the 4,5-Dihydrobenzodiazepinone-Based Compounds **6**–**8**; Reagents and Conditions: (e) **12**/**13**/**16**, Cs_2_CO_3_, Toluene, 85 °C, 14 h, 83–97%; (f) TFA, CH_2_Cl_2_, rt, 2 h, then Fe, AcOH, 100 °C, 4 h, 65–89% over Two Steps; (g) H_2_, 10% Pd/C, EtOAc, rt, 17 h, 99%, then **24**, PyBOP, NEt_3_, DMF, rt, 26–68% over Two Steps; (h) TFA, CH_2_Cl_2_, rt, 2 h, then Zn, NH_4_Cl, DMF, rt, 15 h, then PyBOP, NEt_3_, DMF, rt, 10% over Three Steps

During *in vitro* testing, we noticed that compound **1** underwent a small degree of oxidation when stored in DMSO. While stable for the duration of our assays, after 5 days stirring in deuterated DMSO at 50 °C, we observed that 14% of the material was oxidized from the 3,4-dihydroquinoxalinone to the corresponding quinoxalinone (Scheme 1A, Figure S2A), **5**, which has reduced affinity for the CREBBP bromodomain (data not shown). We reasoned that expansion of the 6-membered 3,4-dihydroquinoxalinone ring to give a 4,5-dihydrobenzodiazepinone ring (**6**) would reduce the propensity for oxidation. From a synthetic perspective, this ring system is accessible as it can be constructed by switching the starting material from an α-amino acid to a β-amino acid.^18^

**Scheme 2:**
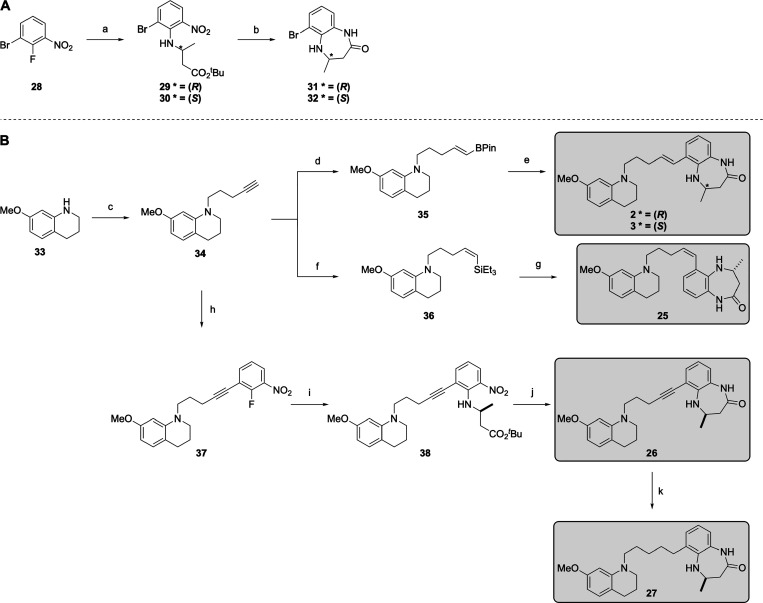
Synthesis of the 4,5-Dihydrobenzodiazepinone-Based Compounds **2**, **3**, and **25**–**27**: (A) Synthesis of the 4,5-Dihydrobenzodiazepinone Headgroup; Reagents and Conditions*:* (a) **12**/**13**, DIPEA, DMF, 85 °C, 14 h, 82–99%; (b) TFA, CH_2_Cl_2_, rt, 2 h, then Fe, AcOH, 100 °C, 4 h, 75–83% over Two Steps; (**B**) Synthesis of 4,5-Dihydrobenzodiazepinone-Based Compounds **2**, **3**, and **25**–**27**; (c) Pent-4-yn-1-yl Methanesulfonate, KI, DIPEA, DMF, μλ, 100 °C, 1 h, 62%; (d) HBPin, Cp_2_ZrClH, DCE, 60 °C, 20 h, 72%; (e) **31/32**, Pd(PPh_3_)_2_Cl_2_, K_2_CO_3_, 1,4-Dioxane, H_2_O, 100 °C, 89–99%; (f) Grubbs I Catalyst, HSiEt_3_, Tol, 40 °C, 2 h, 69%; (g) Vinylsilane, 1 M BCl_3_ in Heptane, CH_2_Cl_2_, 0 °C, 16 h, then **31**, Pd(Amphos)_2_Cl_2_, K_2_CO_3_, Toluene, THF, EtOH, H_2_O, 100 °C, 80% over Two Steps; (h) **29**, Pd(OAc)_2_, Cu(I)I, PPh_3_, NEt_3_, 100 °C, 44%; (i) **12**, DIPEA, DMF, 85 °C, 14 h, 66%; (j) TFA, CH_2_Cl_2_, rt, 2 h, then Fe, AcOH, 100 °C, 30 min, 44%; (k) H_2_, 10% Pd/C, EtOH, rt, 3 h, 78%

To establish whether the larger 4,5-dihydrobenzodiazepinone headgroup would still be accommodated by the CREBBP KAc-binding pocket, we performed docking studies, using AutoDock Vina, followed by 120 ns molecular dynamic simulations (Figure S3). These showed that the 4,5-dihydrobenzodiazepinone was accommodated in the KAc binding pocket and hydrogen bonds were predicted with N1168. Additional hydrophobic interactions were also predicted between the methylene unit and V1115 and I1122, which might lead to increased affinity for the CREBBP bromodomain. In addition, the previously observed cation−π interaction between R1173 and THQ^11^ was also predicted to occur by the MD simulations (Figure S3). These observations encouraged us to proceed with the synthesis of **6**. We also synthesized the enantiomeric compound **7** and the isomer **8**, in which the methyl group is moved from the 4-position to the 3-position of the 4,5-dihydrobenzodiazepinone ring (Scheme 2). The 4,5-dihydrobenzodiazepinone derivatives **6**–**8**, were synthesized as shown in Scheme 1B,C, and a full description of the synthesis is given in the Supporting Information (SI).

To establish whether compound **6** was less prone to oxidation than compound **1**, it was stirred in deuterated DMSO at 50 °C for 5 days. Pleasingly, we saw no evidence of an oxidized product, indicating that **6** is stable under these conditions (Figure S2B).

When tested using ITC and AlphaScreen, compound **6** showed significant CREBBP bromodomain affinity, albeit slightly reduced compared to **1** (Table 1). In the 3,4-dihydroquinoxalinone (**1**) series, the (*S*)-enantiomer showed an 8-fold reduction in affinity compared to the (*R*)-enantiomer.^11^ In the 4,5-dihydrobenzodiazepinone series (**6** and **7**), however, the (*S*)-enantiomer (**7**) showed no detectable affinity for the CREBBP bromodomain. This provides the opportunity to develop a useful inactive companion compound for use as a control in cellular experiments. The isomeric compound **8**, which has the methyl group in the 3-position, only showed low CREBBP bromodomain affinity when tested as a racemate and so was not explored further.

**Table 1:**
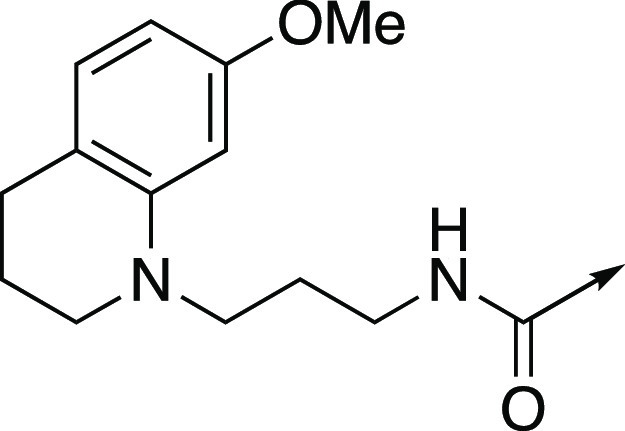

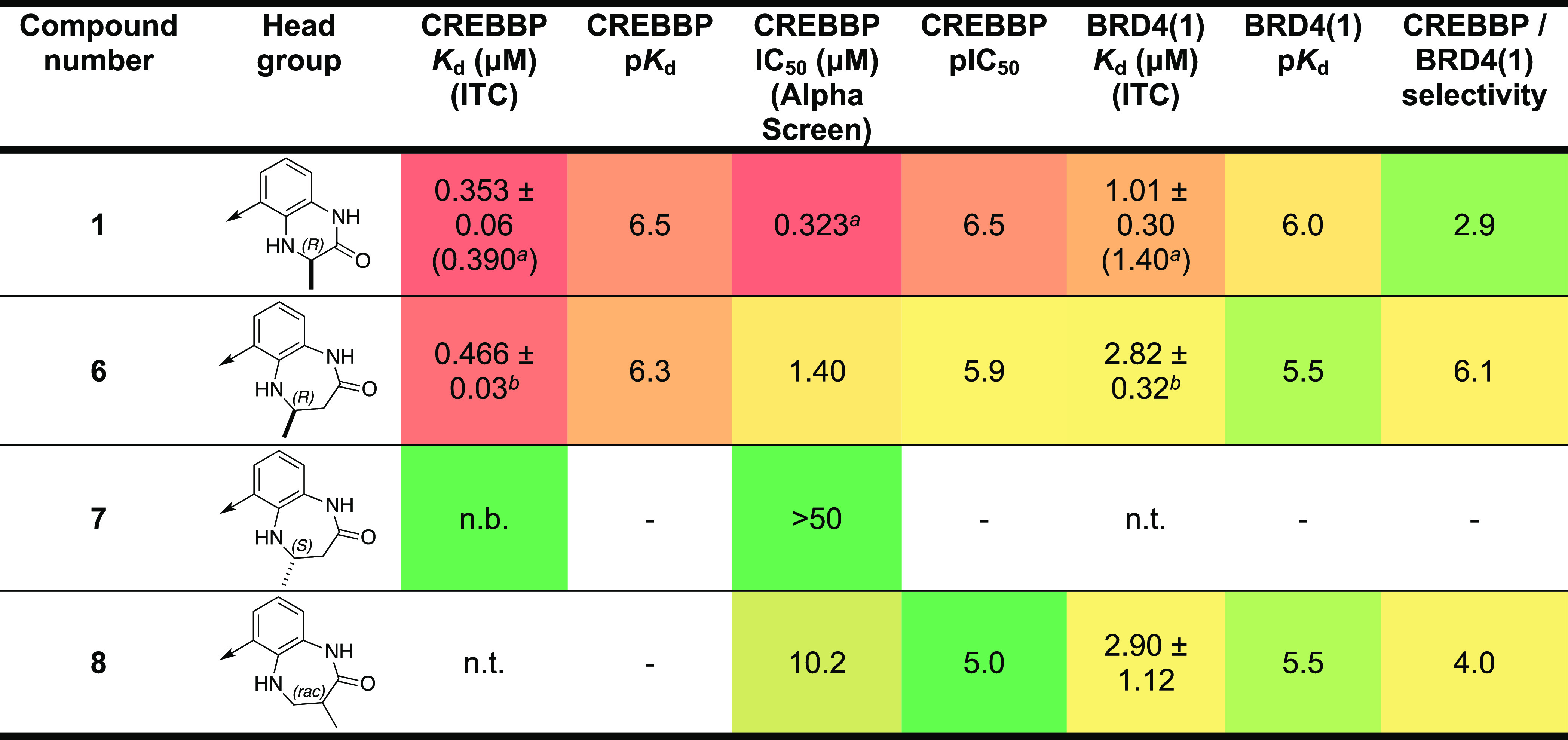
CREBBP and BRD4(1) *K*_d_ Values (ITC), p*K*_d_ Values, CREBBP IC_50_ Values (AlphaScreen), pIC_50_ Values and CREBBP *vs* BRD4(1) Selectivity for Compounds **1** and **6**–**8** (For ITC Plots and Binding Isotherms, See Figures S11 and S12)

a Previously published data.^11^ n.t.: not tested; n.b.: no binding. ± standard error of the fit unless stated. A heatmap representation is used; high affinity is indicated by hot colors.

b *n* = 2 ± s.d. from the mean.

**Figure 3:**
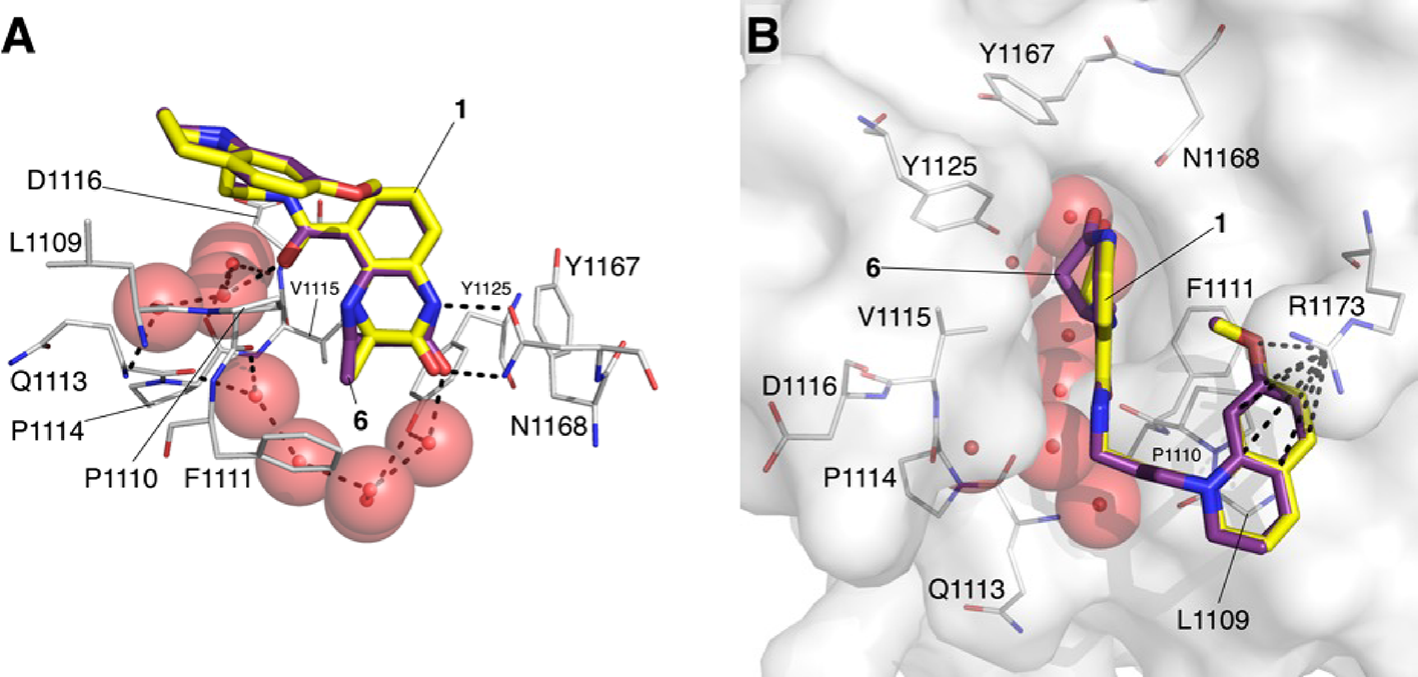
X-ray crystal structure of **6** bound to the CREBBP bromodomain (PDB code: 6YIM; carbon = purple; protein surface from this structure shown) overlaid with the X-ray crystal structure of **1** bound to the CREBBP bromodomain (PDB code: 4NYX; carbon = yellow).^11^ (A) The side orientation shows that the headgroups of each compound form the same hydrogen-bonding interactions with the bromodomain and that the KAc-mimicking methyl and carbonyl groups of both molecules overlay very closely. (B) The top orientation shows that the interaction with R1173 is present for both molecules. The kink in the 4,5-dihydrobenzodiazepinone ring of **6** (carbon = purple), which more fully occupies the KAc-binding pocket, is visible. See Figure S10 for the ligand electron density map.

We obtained an X-ray crystal structure of compound **6** bound to the CREBBP bromodomain (Figure 3; PDB code: 6YIM; carbon = purple). Overlaying this structure with that of **1** bound to the CREBBP bromodomain (PDB code: 4NYX; carbon = yellow) shows that these compounds have very similar binding modes (Figure 3). As with compound **1**, the 4,5-dihydrobenzodiazepinone of **6** forms two hydrogen bonds with N1168. The amide carbonyl oxygen forms hydrogen bonds with a ZA-channel water molecule, and the THQ ring interacts with R1173. The extra methylene unit of the 4,5-dihydrobenzodiazepinone is accommodated by the movement of V1115, relative to the 4NYX structure, and this region of the pocket is more fully occupied by **6** compared to **1** (Figure 3B).

**Figure 4:**
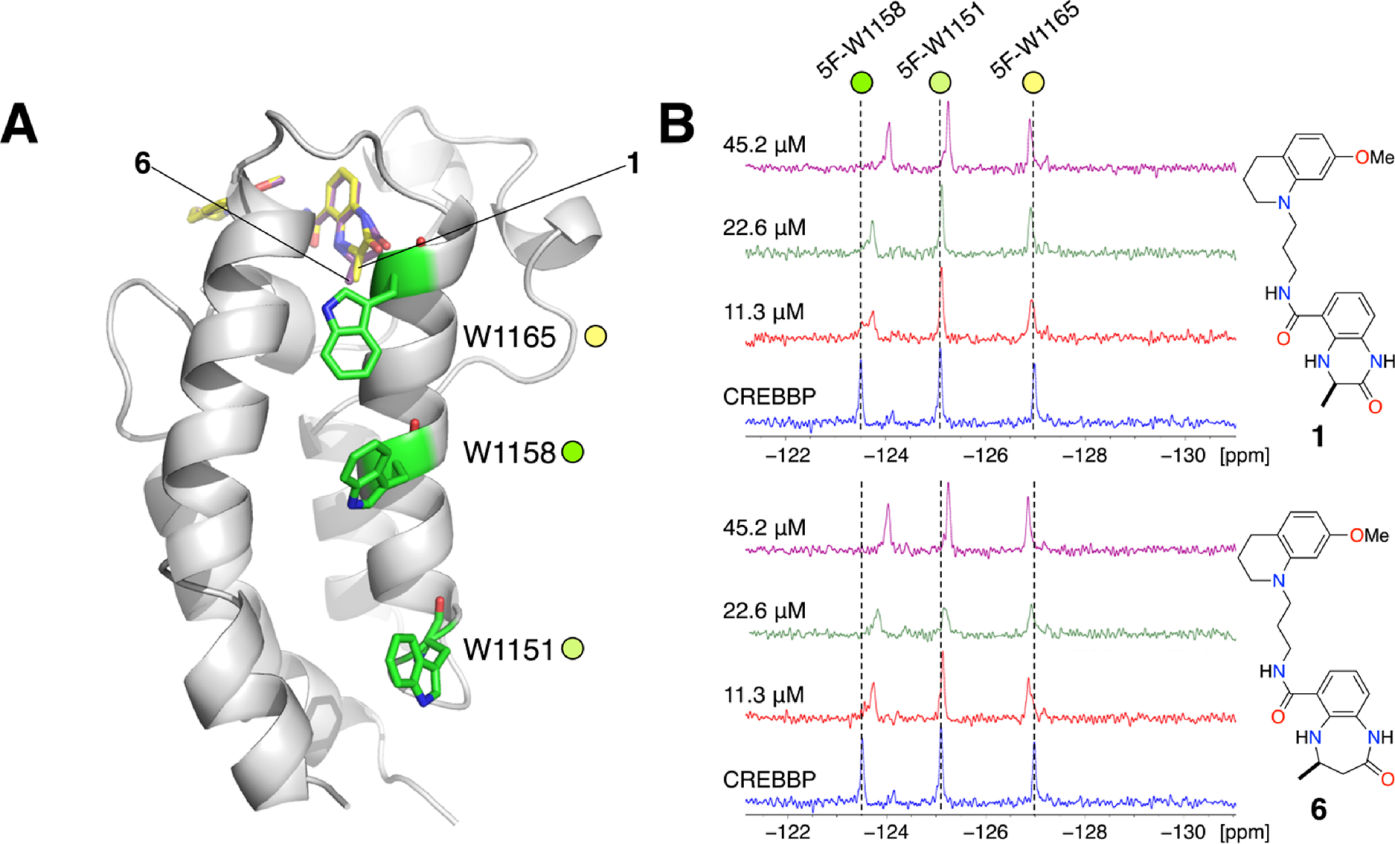
(A) X-ray crystal structure of **6** bound to the CREBBP bromodomain (PDB code: 6YIM; carbon = purple; cartoon from this structure shown) overlaid with the X-ray crystal structure of **1** bound to the CREBBP bromodomain (PDB code: 4NYX; carbon = yellow).^11^ The three tryptophan residues (W1151, W1158, and W1165) that were replaced by 5-fluorotryptophan are highlighted as green sticks. (B) Partial ^19^F NMR spectra showing the effect of adding 11.3, 22.6, or 45.2 μM of either **1** or **6** to the 5-fluorotryptophan-containing CREBBP bromodomain (45 μM). The resonances were assigned to specific 5-FW residues using point mutation studies (Figure S4C).

To gain an insight into the solution-state binding of compounds **1** and **6** to the CREBBP bromodomain, we employed protein-observed ^19^F (PrOF) NMR.^45,46^ To enable this approach, a fluorine-labeled CREBBP bromodomain was expressed in which the three naturally occurring tryptophan residues were replaced with 5-fluorotryptophan (5-FW, Figure 4A and Figure S4). Unlike our previous reports of fluorinated bromodomains employed in PrOF NMR analysis, these tryptophans reside outside the binding site. Consequently, a change in their chemical environment can result from allosteric, or large scale, structural changes.

The incorporated 5-fluorotryptophan residues give three distinct signals in the ^19^F NMR. Point mutation studies enabled assignment of the resonance at −123.5 ppm to 5F-W1158, the resonance at −125.0 ppm to 5F-W1151, and the resonance at −127.0 ppm to 5F-W1165 (Figure 4B and Figure S4C). Upon the addition of compound **1** (11.3, 22.6 or 45.2 μM), the ^19^F NMR signal at −123.5 ppm (5-FW1158) shifted upfield to −124.0 ppm and the resonance at −125.0 ppm (5F-W1151) showed slight shift at the highest concentration. The resonance at −127.0 ppm (5-FW1165) was unaffected (Figure 4B). The addition of compound **6** (11.3, 22.6 or 45.2 μM) also resulted in a shift of 0.5 ppm for the resonance at −123.5 ppm (5-FW1158). In addition, the other two resonances show small but discernible shifts, especially at the highest concentration. It is interesting that the resonance assigned to 5-FW1158 was most affected by ligand binding, as this residue superficially appears to be further removed from the ligand binding site than 5F-W1165. It is possible that the allosteric effects of ligand binding on 5-FW1158 are mediated by the water molecules found in the KAc-binding pocket of the bromodomain and residues M1160 and F1161. Rearrangement of the water molecules likely affects the environments of M1160 and F1161, which are in close proximity to W1158. F1161 appears to form edge-to-face interactions with the indole ring, and so a change in position for F1161 will substantially affect the environment of 5-FW1158, changing its chemical shift. The observation that all three resonances move upon the addition of **6** supports the idea that a structural rearrangement is required to accommodate the larger 4,5-dihydrobenzodiazepinone ring in the KAc-binding pocket, resulting in more London dispersion forces between **6** and V1115 than for compound **1**.

Interestingly, when the experiment was repeated with I-CBP112,^16^ a similar shift was observed for the resonance at −123.5 ppm (5-FW1158) but in the opposite direction (Figure S4A). The addition of bromosporine,^47^ which is a lower affinity ligand for the CREBBP bromodomain, did not affect the ^19^F chemical shifts (Figure S4B). While further investigation is needed, these data are consistent with the idea that **6** and I-CBP112 both bind to the bromodomain but cause different structural rearrangements in the protein, which might result in different allosteric effects in the full-length protein (*vide infra*). Although these results show that the 4,5-dihydrobenzodiazepinone motif was accommodated by the CREBBP bromodomain KAc-binding pocket, it did not result in the predicted increase in affinity, which was surprising. This observation led us to consider the effects of intramolecular hydrogens bonds in dictating the solution-state conformation of our CREBBP bromodomain ligands and whether this affects ligand affinity.

### Investigating the Intramolecular Hydrogen Bond

To investigate the presence of intramolecular hydrogen bonds in solution, we have previously employed a ^1^H NMR-based approach.^48,49^ When changing the NMR solvent from CDCl_3_ to DMSO-*d*_6_ the chemical shift of hydrogen atoms that are not involved in hydrogen bonds typically show a Δ_ppm_ CDCl_3_ → DMSO-*d*_6_ = 2–4 ppm. This shift results from the solvent having the greatest effect on the environment of these atoms. Hydrogen atoms that are participating in an internal hydrogen bond generally show Δ_ppm_ CDCl_3_ → DMSO-*d*_6_ < 1 ppm. The environment of these atoms is mainly affected by the intramolecular interaction and is less affected by the surrounding solvent. When combined with structural studies, this technique allows us to assess whether intramolecular hydrogen bonds formed in solution are also present when the ligand is bound to a protein.

**Figure 5:**
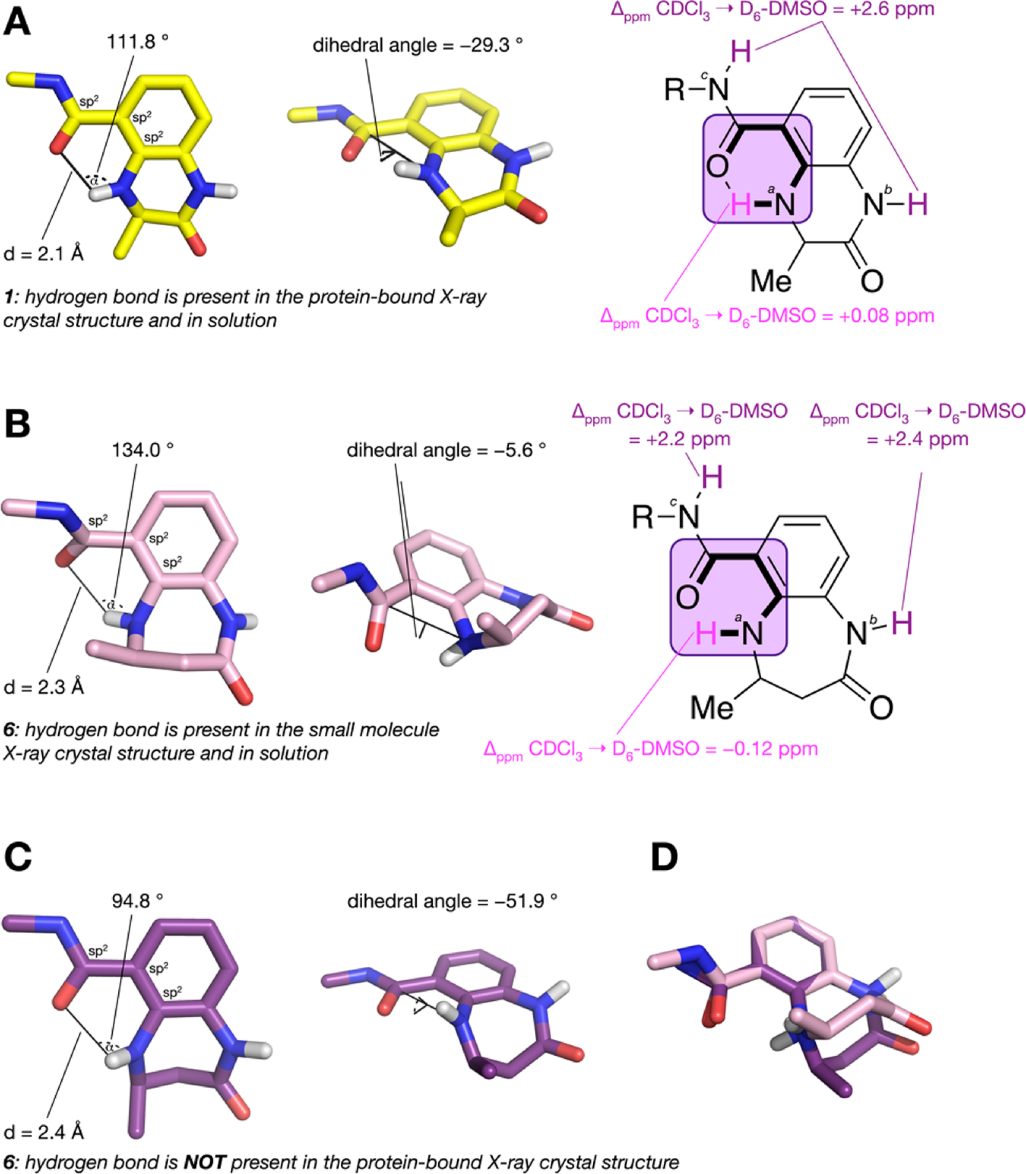
(A) A resonance-assisted hydrogen bond is observed when **1** binds to the CREBBP bromodomain as α > 100 °, *d* < 2.35 Å, and an O–C–N–H dihedral angle of −29.3 ° is observed (PDB code: 4NYX; carbon = yellow). A Δ_ppm_ CDCl_3_ → DMSO-*d*_6_ of 0.08 ppm for *^a^*NH indicates that the intramolecular hydrogen bond is present in solution. (B) In a small-molecule X-ray crystal structure of **6**, a resonance-assisted hydrogen bond is observed as α > 100 °, *d* < 2.35 Å, and an O-C-N-H dihedral angle of −5.6 ° is observed (CCDC code: 1993652-3; carbon = pink). A Δ_ppm_ CDCl_3_ → DMSO-*d*_6_ of −0.12 ppm for *^a^*NH indicates that the intramolecular hydrogen bond is also present in solution. (C) A resonance-assisted hydrogen bond is not observed when **6** binds to the CREBBP bromodomain as α < 100 °, *d* > 2.35 Å, and an O–C–N–H dihedral angle of −51.9 ° is observed (PDB code: 6YIM; carbon = purple). (D) Overlay of the small molecule X-ray crystal structure (CCDC code: 1993652-3; carbon = pink) with the conformation of **6** observed in the X-ray crystal structure of this compound bound to the CREBBP bromodomain CREBBP (PDB code: 6YIM; carbon = purple). Low-temperature single crystal X-ray diffraction data for **6** were collected using a Rigaku Oxford SuperNova diffractometer. Raw frame data were reduced using CrysAlisPro, and the structures were solved using “Superflip”^51^ before refinement with CRYSTALS^52,53^ as per the SI. Full refinement details are given in the Supporting Information; Crystallographic data have been deposited with the Cambridge Crystallographic Data Centre (CCDC 1993652-3).

During the development of compound **1**,^11^ we hypothesized that a resonance-assisted intramolecular hydrogen bond was present in the X-ray crystal structure of this compound bound to the CREBBP bromodomain.^50^ In addition, we suggest that the O–C–N–H dihedral angle (Figure 5) should be sufficiently small to allow the n → σ*_N-H_ overlap that is a key component of hydrogen bonding. These criteria are met by compound **1**, indicating that this hydrogen bond exists in the X-ray crystal structure. We previously hypothesized that this hydrogen bond is also present in solution, which reduces the entropic penalty of **1** binding to the CREBBP bromodomain and results in a high affinity for this protein. Using ^1^H NMR studies, we see that the chemical shifts for *^b^*NH and *^c^*NH of compound **1** (Figure 5A) have a Δ_ppm_ CDCl_3_ → DMSO-*d*_6_ of 2.6 ppm, showing that, as expected, these atoms are not involved in hydrogen bonds. For *^a^*NH, however, we see a Δ_ppm_ CDCl_3_ → DMSO-*d*_6_ of only 0.08 ppm. These data are consistent with the presence of an intramolecular hydrogen bond in solution. This observation supports our hypothesis that the intramolecular hydrogen bond helps to pre-organize the solution state conformation of **1** to one that is similar to the protein-bound conformation, resulting in a reduced entropy penalty for protein binding and high affinity for the CREBBP bromodomain.

For compound **6**, the chemical shifts for *^b^*NH and *^c^*NH (Figure 5B) have a Δ_ppm_ CDCl_3_ → DMSO-*d*_6_ of 2.4 and 2.2 ppm, respectively, indicating that, as expected, these atoms are not involved in hydrogen bonds. For *^a^*NH, we see a Δ_ppm_ CDCl_3_ → DMSO-*d*_6_ of −0.12 ppm, indicating the presence of an intramolecular hydrogen bond in solution (Figure 5B and Figure S5). This hydrogen bond is also observed in a small molecular X-ray crystal structure of **6** as α > 100 °, *d* < 2.35 Å, and an O–C–N–H dihedral angle of −5.6 ° is observed (CCDC code: 1993652-3; carbon = pink; Figure 5B). However, the analysis of the X-ray crystal structure of **6** bound to the CREBBP bromodomain shows an α of <100 °, *d* > 2.35 Å, and a dihedral angle of −51.9°, suggesting that there is no intramolecular hydrogen bond present when **6** is bound to the CREBBP bromodomain (Figure 5C). An overlay of the free and protein-bound X-ray crystal structures (Figure 5D) indicates that the 4,5-dihydrobenzodiazepinone ring has to undergo a ring flip to bind the CREBBP bromodomain. For this to occur, the intramolecular hydrogen bond must be broken, resulting in an enthalpic penalty and compound **6** having lower affinity than **1** for the CREBBP bromodomain. If this hypothesis is correct, then the replacement of the amide with a bioisostere that cannot hydrogen bond with *^a^*NH, but retains the amide bond rigidity, should lead to a higher affinity CREBBP bromodomain ligand. It is interesting to note that the 4,5-dihydrobenzodiazepinone ring adopts the same conformation as **6** in the X-ray crystal structure of CPI-637 bound to the CREBBP bromodomain (PDB code: 5I8G; Figure S13). This observation is consistent with the idea that the ring-flipped conformation is required for CREBBP bromodomain binding.

### Introducing Amide Bioisosteres

**Figure 6:**
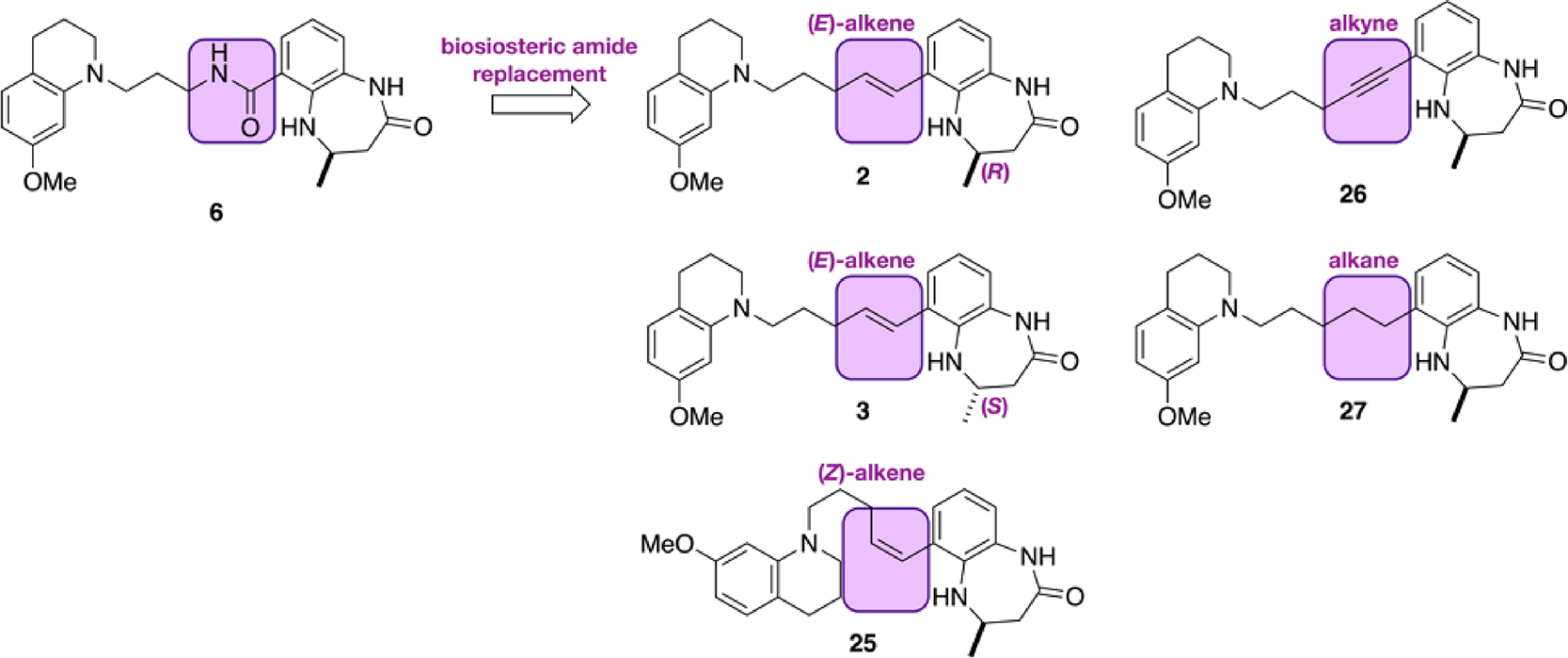
To investigate the effect of an intramolecular hydrogen bond on CREBBP bromodomain affinity, we designed compounds in which the amide bond is replaced with an (*E*)-alkene (**2**). For comparison, we also designed compounds containing a (*Z*)-alkene (**25**), an alkyne (**26**), and an alkane (**27**). We included the opposite enantiomer (**3**) of **2**, which we predicted would show no CREBBP bromodomain activity.

To investigate this idea, we sought to identify an amide bioisostere that mimics the geometric constrains of the peptide bond, but which could not act as a hydrogen bond donor or acceptor. We predicted that such a group would maintain some pre-organization of the compound but without the need to break the intramolecular hydrogen bond to allow protein binding, resulting in a higher affinity CREBBP bromodomain ligand. Based on these criteria, we selected an (*E*)-alkene as the simplest amide bioisostere (Figure 6).^54^ In addition, we decided to make the (*Z*)-alkene (**25**), the alkyne (**26**), and the alkane (**27**) derivatives for comparison. We predicted that the enantiomeric (*E*)-alkene (**3**) would show no affinity for the CREBBP bromodomain. The 4,5-dihydrobenzodiazepinone derivatives **2**, **3**, **25**, **26**, and **27** were synthesized as shown in Scheme 2, and a full description of the synthesis is given in the SI.

Replacement of the amide (**6**) for the (*E*)-alkene resulted in compound **2**, which has a *K*_d_ = 102 ± 10 nM (ITC) for the CREBBP bromodomain (Table 2). Comparison of the ITC signature plot for **6** and **2** shows that the Δ*H* component is smaller for **6** than for **2**, consistent with the hypothesis that the intramolecular hydrogen bond in **6** is being broken upon protein binding. Interestingly, the −*T*Δ*S* term is smaller for **2** than that for **6**, suggesting that removal of the hydrogen bond does reduce some solution-phase pre-organization of **2** (Figure S6A,B). As expected, the enantiomer, **3**, showed no CREBBP bromodomain affinity. The (*Z*)-alkene- and alkyne-containing compounds (**25** and **26**) retain significant affinity for the CREBBP bromodomain with *K*_d_ = 0.329 ± 0.08 nM and *K*_d_ = 0.154 ± 0.03 nM (ITC), respectively. The increased affinity of the alkene, *versus* the amide **6**, supports our hypothesis that the intramolecular hydrogen bond observed in **6** is detrimental to CREBBP bromodomain binding. It is also interesting that both the (*Z*)-alkene (**25**) and the alkyne (**26**) retain CREBBP bromodomain affinity. Molecular dynamics simulations (*vide infra*) predict that these compounds can be accommodated in the CREBBP KAc-binding site (Figure S7). Perhaps surprisingly, the alkane **27** shows no detectable affinity for the CREBBP bromodomain, indicating that some degree of structural pre-organization is required for CREBBP bromodomain affinity (Table 2).

**Table 2:**
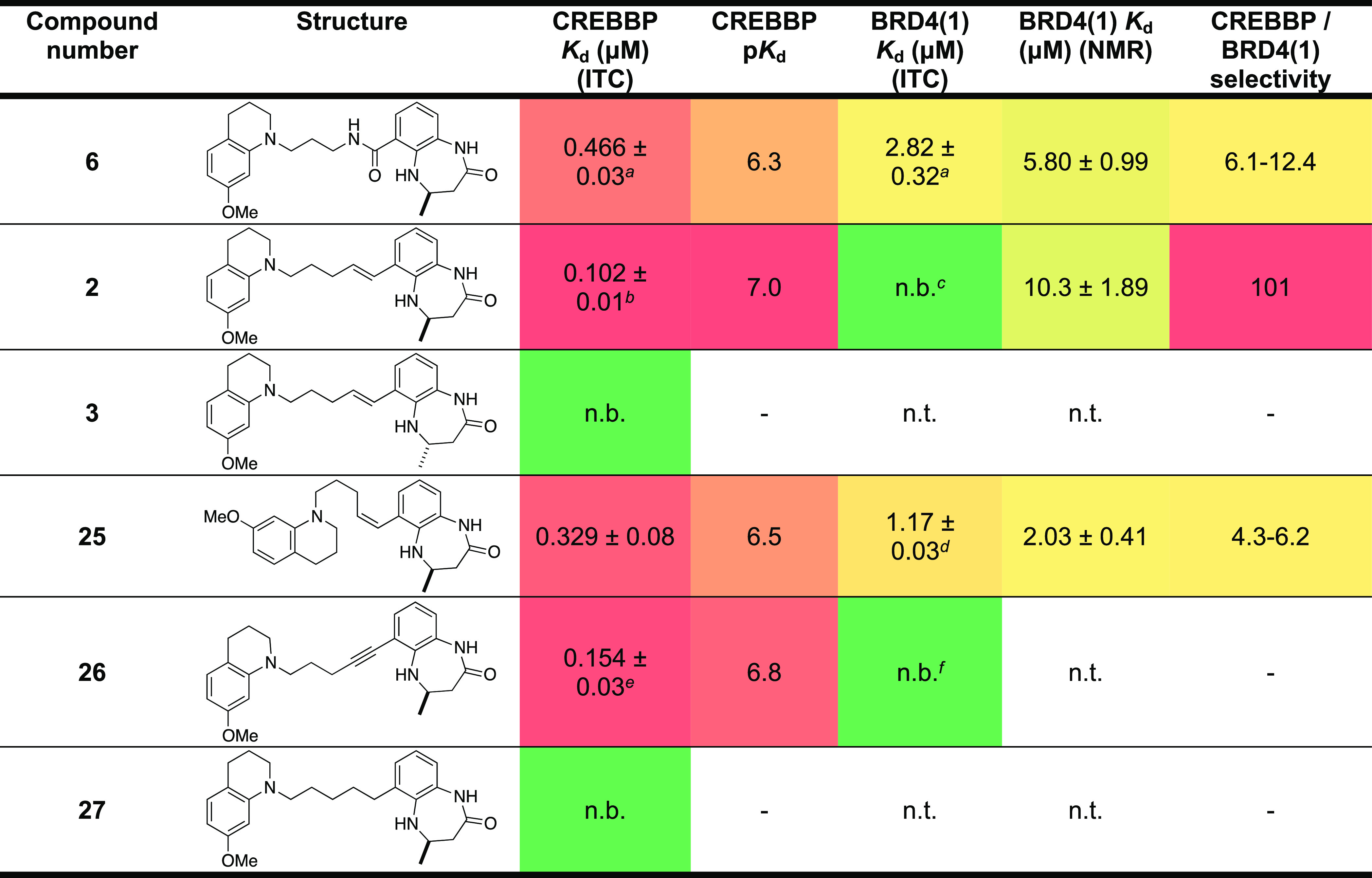
CREBBP and BRD4(1) *K*_d_ Values (ITC and NMR), p*K*_d_ Values, and CREBBP *vs* BRD4(1) Selectivity for Compounds **6**, **2**, **3**, and **25**–**27** (For ITC Plots and Binding Isotherms, See Figures S11 and S12)^g^

a *n* = 2 ± s.d. from the mean.

b *n* = 5 ± s.d. from the mean.

c *n* = 4.

d *n* = 2 ± s.d. from the mean.

e *n* = 5 ± s.d. from the mean.

f *n* = 2.

g n.t.: not tested; n.b.: no binding. ± Standard error of the fit unless stated. A heatmap representation is used; high affinity is indicated by hot colors.

We initially tried to assess the CREBBP/BRD4(1) selectivity of **2** using ITC. However, under the conditions used, we were unable to detect binding to BRD4(1). Instead, we used ligand-observed ^1^H NMR with protein titration (Figure S8), which gave estimated *K*_d_ values of 5.80 ± 0.99 μM for **6**, 2.03 ± 0.41 μM for the (*Z*)-alkene **25**, and 10.3 ± 1.89 μM for **2**. Pleasingly, these data indicate that **2** is >100-fold selective for CREBBP *versus* BRD4(1).

**Figure 7:**
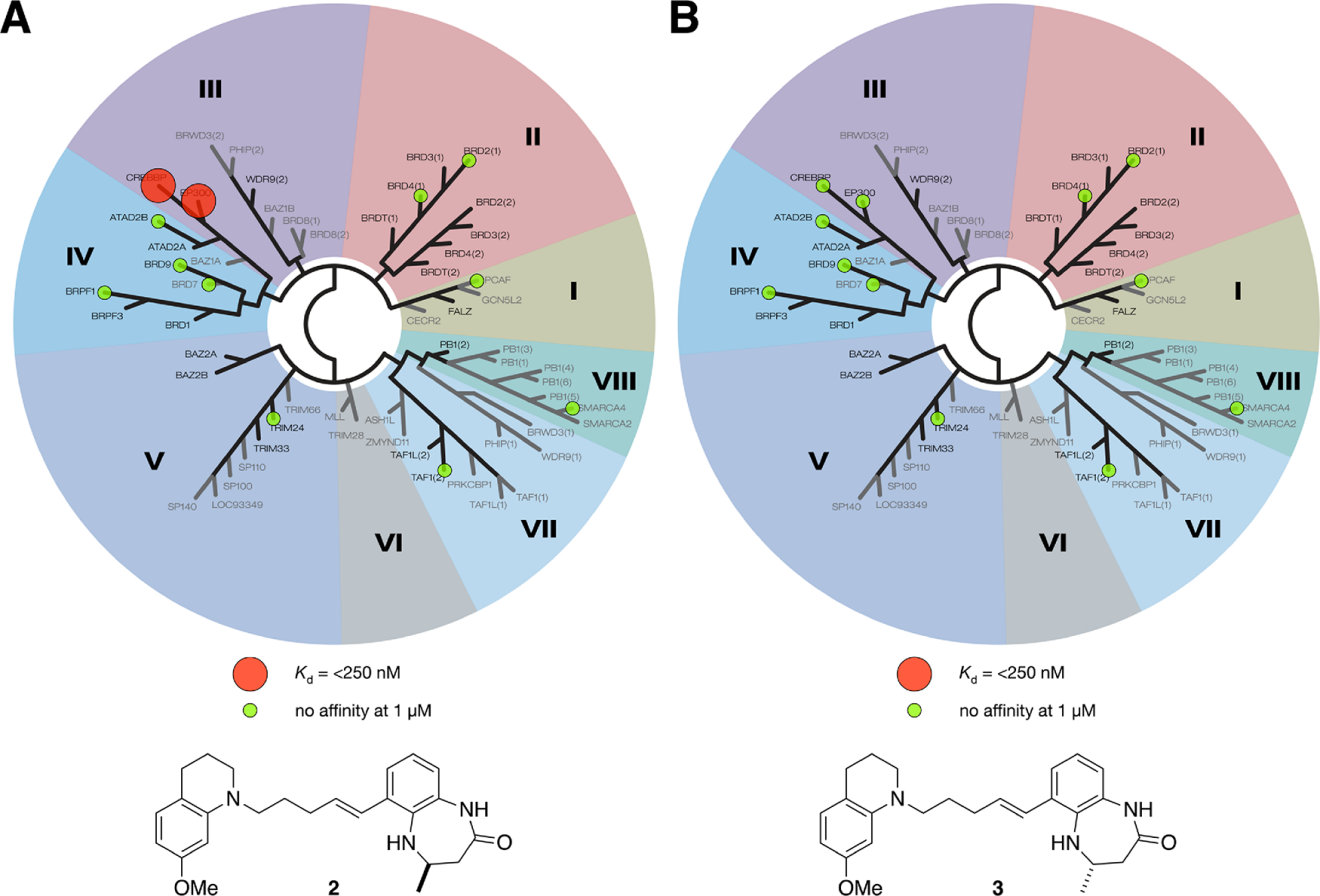
Representation of the bromoKdELECT data for (A) compound **2** and (B) compound **3**. Using the BROMOscan platform, *K*_d_ values were determined for 12 phylogenetically diverse bromodomains (ATAD2B, BRD2(1), BRD4(1), BRD7, BRD9, BRPF1, CREBBP, EP300, PCAF, SMARCA4, TAF1(2), and TRIM24). Compound **2** showed *K*_d_ values of 200 nM and 230 nM for the CREBBP and EP300 bromodomains, respectively. No binding was seen at any of the other bromodomains at concentrations of up to 1 μM. Compound **3** showed no binding to any of the other bromodomains at concentrations of up to 1 μM.

To further assess the selectivity of **2**, we subjected it to a BROMOscan to determine its *K*_d_ values against a phylogenetically diverse panel of 12 bromodomains (Figure 7). In this assay, **2** showed *K*_d_ values of 200 and 240 nM for the CREBBP and EP300 bromodomains, respectively. No binding was seen at any of the other bromodomains at concentrations of up to 1 μM. In the same assay, compound **3** showed no binding to any of the bromodomains tested at concentrations of up to 1 μM. These data show that compound **2** is suitable for use in a cellular setting to probe the effect of inhibiting CREBBP and EP300 bromodomain functions.

### Analysis of CREBBP Bromodomain Ligand Binding and Selectivity

**Figure 8:**
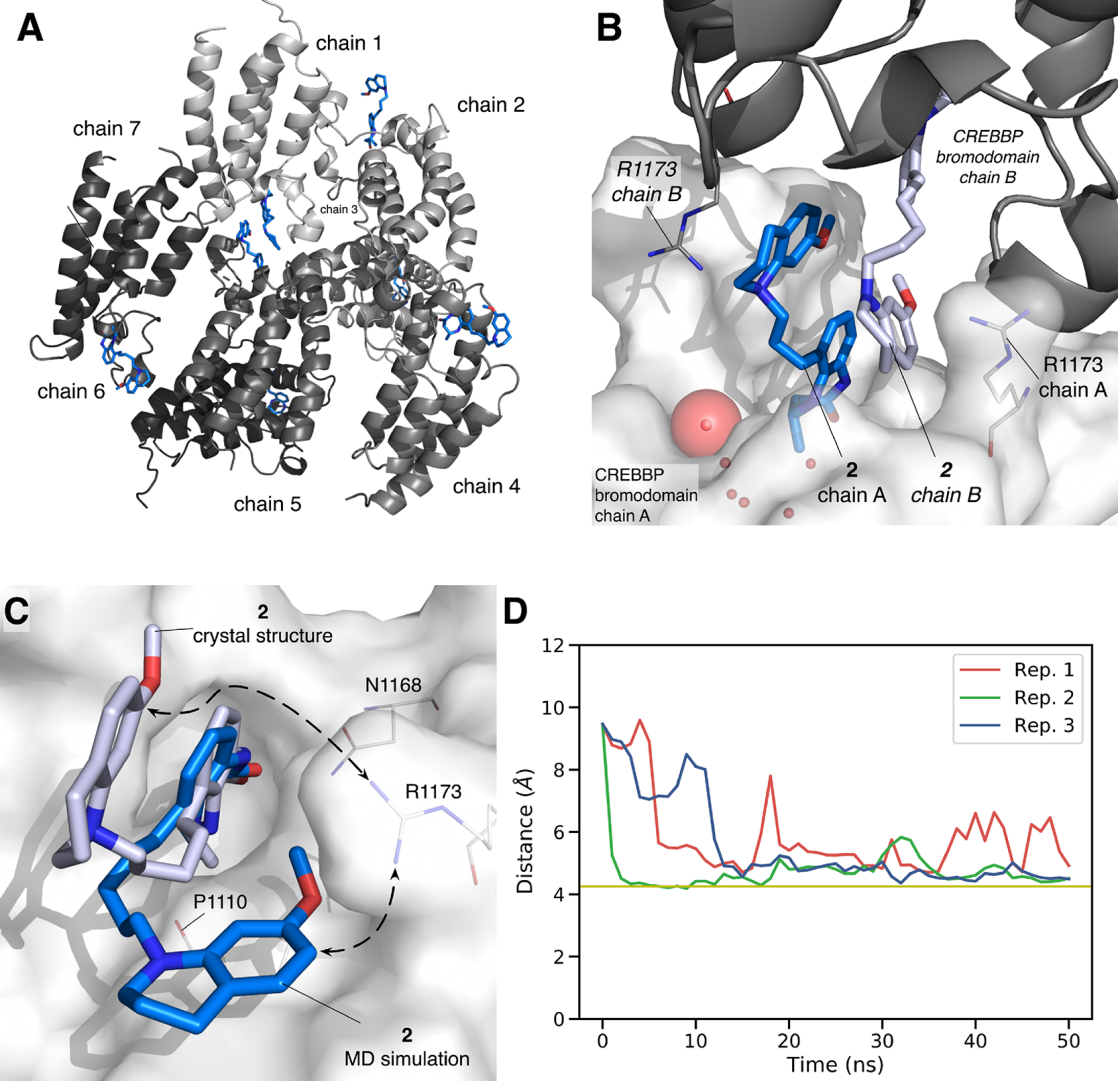
X-ray crystal structure of compound **2** bound to the CREBBP bromodomain (PDB code: 6YIJ). (A) Seven chains are observed in the unit cell. (B) The bromodomains form dimeric units in the crystal structure, with the ligand that is bound to chain A interacting with the R1173 residue of the cognate chain B. See Figure S10B for ligand electron density map. (C) MD simulations predict that the electrostatic interaction between **2** and R1173 is present in solution. (D) The distance between R1173 and the π system of **2** drops to below 7 Å during equilibrium MD simulations meaning that the pose observed in the X-ray crystal structure (PDB code: 6YIJ) relaxes to give a conformation that is similar to that observed for **6** bound to the CREBBP bromodomain (PDB code: 6YIM; Figure 3). Simulations were carried out in triplicate.

An X-ray crystal structure of **2** bound to the CREBBP bromodomain revealed that the 4,5-dihydrobenzodiazepinone headgroup bound as expected in the KAc-binding pocket. The full structure is, however, more complex, with 7 proteins observed in the asymmetric unit (Figure 8A). Surprisingly, the THQ group formed an interaction with the R1173 residue of an adjacent protein, resulting in a dimeric structure with two ligands bound to two bromodomains (Figure 8B). We used size-exclusion chromatography (SEC) to determine whether the dimeric structure occurred in solution or is a crystallographic artifact. This method has been used by others for the verification of bivalent ligands.^55,56^ These experiments indicated that dimerization is not occurring in solution, as an identical peak was observed in both the presence or absence of compound **2** (50 μM, Figure S9).

As the SEC results indicate that a dimeric structure is not present in solution, we used molecular dynamics simulation to predict the monomeric structure of **2** bound to a single CREBBP bromodomain. Starting from a single bromodomain, taken from the X-ray crystal structure (PDB code: 6YIJ), molecular dynamics simulations (50 ns run in triplicate, Figure S12) predict that the THQ group moves closer to P1110 settling at a distance of ∼4.2 Å. This residue is located below R1173, supporting the hypothesis that **2** has a similar binding mode to compounds **1** and **6** when bound to the CREBBP bromodomain.

Docking studies on the (*Z*)-alkene **25** and the alkyne **26** (Figure S7) predict that these compounds bind to the CREBBP bromodomain in a similar conformation to **6**. As with compound **2** (Figure 8C), molecular dynamics simulation (50 ns run in triplicate) were carried out starting from either the X-ray protein crystal structure for compound **6** (PDB code 6YIM), or the docked structures for compounds **25** and **26**. In all cases, these simulations predict that the THQ group resides close to P1110 and in the proximity of R1173 (Figure 8D). The fact that compounds **25** and **26** can form the same interaction as **2** is consistent with their measured binding affinity for the CREBBP bromodomain.

### Rationalizing the CREBBP *vs* BRD4(1) Selectivity

**Figure 9:**
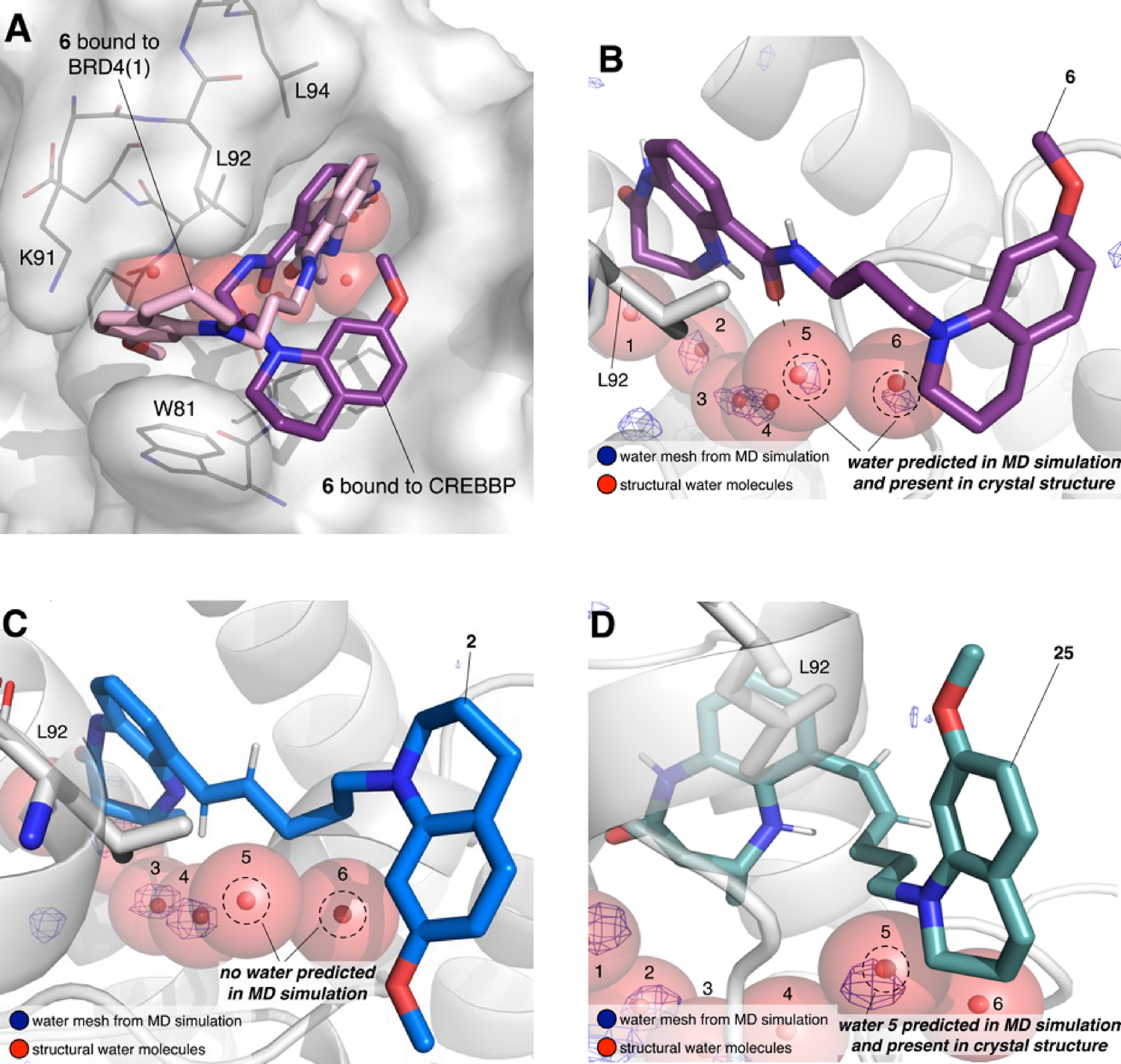
(A) Overlay of the X-ray crystal structure of compound **6** bound to BRD4(1) (PDB ID: 6YIN; carbon = pink) and the X-ray crystal structure of compound **6** bound to the CREBBP bromodomain (PDB ID: 6YIM; carbon = purple). The surface of BRD4(1) is shown in white. The conformation of **6** when bound to BRD4(1) is more extended than that when bound to the CREBBP bromodomain. This conformation allows **65** to interact with W81, which likely contributes to its affinity for BRD4(1). (B–D) Snapshots of molecular dynamics simulations (taken at 25 ns) starting from the (B) X-ray crystal structure of **6** (carbon = purple) bound to BRD4(1) (PDB code: 6YIN), (C) the structure of **2** (carbon = blue) docked to BRD4(1) following a 25 ns MD simulation, or (D) the structure of **25** (carbon = teal) docked to BRD4(1) following a 25 ns MD simulation. Crystallographic water molecules from BRD4(1) (PDB code: 6YIN) are shown as red spheres. Areas of high water density are shown as a blue mesh. In (B) and (D), the water densities overlay the crystallographic water molecules, indicating that this binding pose is stable. In (C), no water density is observed, meaning that the crystallographic water molecules would have to be ejected to accommodate the binding of **2**. As the displacement of the crystallographic water molecules is likely to be unfavorable, **2** does not bind to BRD4(1), resulting in the high selectivity of this compound for the CREBBP bromodomain over BRD4(1).

We were particularly intrigued as to how the modest change from an amide to an alkene resulted in such a substantial increase in selectivity for CREBBP *versus* BRD4(1). An X-ray crystal structure of the amide **6** bound to BRD4(1) was the key to our understanding of this observation. This structure revealed that **6** adopts a very different conformation when bound to BRD4(1) compared to when it is bound to the CREBBP bromodomain (Figure 9A). When bound to CREBBP, the ligand adopts a curved conformation so as to interact with P1110 and R1173; when bound to BRD4(1), it adopts an extended conformation resulting in London dispersion forces between the THQ ring, W81, and L92. This type of conformation has been observed with other BET bromodomain ligands, including I-BET762^57^ and BI-2536.^58^

We reasoned that the low affinity of **2** for BRD4(1) indicates that it cannot adopt the same conformation as **6** when bound to this bromodomain. Docking studies indicated that **2** can initially bind to BRD4(1) in a similar orientation to **6**, but 25 ns MD simulations show that the linker of **2** did not hold a stable orientation relative to the 4,5-dihydrobenzodiazepinone headgroup. The key to this observation lies in the interactions with the BRD4(1) ZA channel water molecules. A 25 ns MD simulation starting from the X-ray crystal structure of **6** bound to BRD4(1) (Figure 9B) predicts a high density of water in the ZA channel. This observation is consistent with the presence of a hydrogen bond between the amide carbonyl oxygen atom of **6** and the BRD4(1) ZA-channel water molecule, which is observed in the X-ray crystal structure (PDB code: 6YIN). In the simulation of **2** bound to BRD4(1), there is no water density present in the same region (Figure 9C). This indicates that this water molecule would have to be displaced for **2** to bind to BRD4(1), presumably because **2** cannot form a hydrogen bond. As this water molecule is very hard to displace, compound **2** is unable to bind to BRD4(1), which is reflected in its very low measured affinity.

Interestingly, docking of the (*Z*)-alkene **25**, which shows low μM affinity for BRD4(1), identified two poses where the THQ could reside against either L92 or W81. Both poses were subject to MD studies, which showed that both locations of the THQ group allowed it to form interactions with L92, W81, and P82. Both of these poses hold the (*Z*)-alkene bond perpendicular to the KAc mimic and therefore do not result in the displacement of both ZA channel water molecules (Figure 9D). It seems, therefore, that ligands that can interact with or accommodate this water molecule will retain affinity for BRD4(1), whereas those that cannot, including **2**, are not able to bind to BRD4(1), resulting in high selectivity over this bromodomain.

### The Effects of CREBBP and EP300 Bromodomain Inhibition in HCT116 Cells

**Figure 10:**
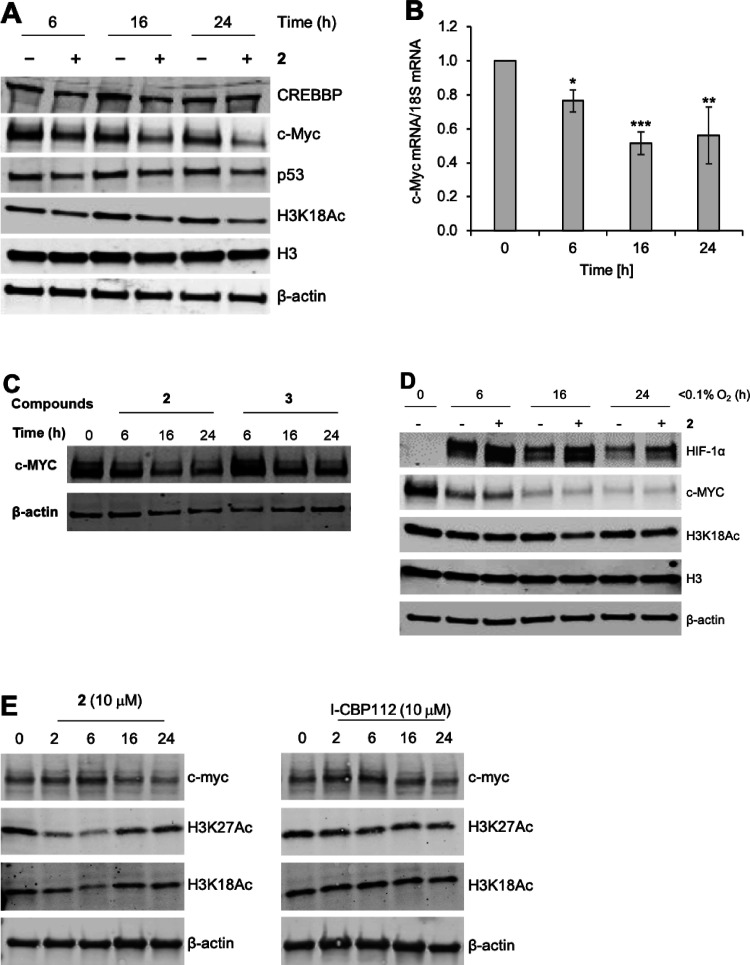
Inhibition of CREBBP/EP300 with **2** reduces c-Myc levels and acetylation of H3K18 in HCT116 cells (human colorectal cancer cell line) and stabilizes HIF-1α in hypoxia. (A) HCT116 cells were exposed to **2** (10 μM) for the times shown. Western blots were then carried out for the proteins indicated. β-Actin is included as a loading control. (B) HCT116 cells were exposed to **2** (10 μM) for the times indicated followed by mRNA preparation. Q-PCR was then carried out to determine c-Myc mRNA levels compared to the 18S. Data were analyzed using one-way ANOVA. *P < 0.05, **P < 0.01, ***P < 0.001. (C) The inactive compound **3** does not reduce levels of c-Myc. (D) HCT116 cells were exposed to compound **2** (10 μM) in hypoxia for the times shown. c-Myc was reduced in hypoxia, which is a known phenomenon. The stabilization of HIF-1α above the level observed in hypoxia alone was observed. (E) HCT116 cells were exposed to **2** (10 μM) or I-CBP112 for the times indicated. Treatment with both compounds resulted in a decrease in H3K27Ac and c-Myc. A decrease in H3K18Ac was observed upon treatment with **2** but not I-CBP112.

With a *K*_d_ value of 102 ± 10 nM, high selectivity over BRD4(1), and no effects on a phylogenetically-diverse panel of bromodomains, compound **2** is suitable for use as a probe of CREBBP/EP300 bromodomain function in cells. Treatment of human colorectal cancer cells (HCT116) with **2** (10 μM) reduces expression of c-Myc after 6 h, with more substantial effects observed after 16 and 24 h (Figure 10A–E). These effects were also observed on c-Myc mRNA (Figure 10B), confirming that the inhibition of the CREPPB/EP300 bromodomain affects c-Myc at the transcriptional level. Treatment of the same cells with the inactive enantiomer, **3** (10 μM), had no effect on c-Myc expression after 24 h (Figure 10C), supporting the hypothesis that these effects occur as a result of CREBBP/EP300 bromodomain inhibition. These data are consistent with previous reports that selective inhibition of the CREBBP bromodomain results in reduced levels of c-Myc.^17−19,59^

In addition to modulating c-Myc levels, we also observed that treatment of HCT116 cells with **2** (10 μM) caused a decrease in H3K18Ac and H3K27Ac (Figure 10A–E). Treatment of the same cell line with I-CPB112 (10 μM) also resulted in a modest decrease of H3K27Ac but not H3K18Ac to any substantial extent (Figure 10E). Interestingly, the reduction in both H3K18Ac and H3K27Ac caused by **2** was most pronounced at 6 h (∼30% of *t* = 0) but had recovered (to ∼70% of *t* = 0) by 24 h.

Modulation of H3K27Ac by CREBBP bromodomain inhibitors is well established,^60,61^ and the recovery of H3K18Ac and H3K27Ac levels is consistent with the observation that CREBBP-regulated sites display rapid acetylation and deacetylation kinetics.^8^ It is interesting that Zucconi *et al.*(62) observed an increase in H3K18Ac in an acute myeloid leukemia line (KG1a) and an androgen-dependent prostate cancer cell line (LNCaP) upon treatment with the CREBBP/EP300 bromodomain ligand I-CBP112 (10 or 20 μM). However, this effect was not observed upon treatment with CPB30. While Conery *et al.* did observe a locus specific reduction in H3K18Ac in multiple myeloma cell lines,^63^ they did not observe global changes in H3K18Ac upon treatment with CPB30.

The reasons for the differences in these data are not clear, and it should be noted that different cell lines are used but this could hint at some CREBBP/EP300 bromodomain ligands having an allosteric modulatory effect on the KAT activity of CREBBP/EP300. This is consistent with our PrOF NMR studies (*vide supra*), which showed shifts in the fluorine for **2** and I-CPB112, suggesting that allosteric effects could depend on the structure of the bromodomain ligand. CPB30, which possesses the smaller 3,5-dimethylisoxazole KAc mimic,^15,41,64,65^ might not have an allosteric effect on the KAT domain. We note that antibody-related variations in the visualization of the acetylation marks discussed could also affect the levels of histone lysine acetylation detected.

In hypoxia (<0.1% O_2_), levels of c-Myc were depleted in both the absence and presence of **2**. The depletion of c-Myc in hypoxia is a known phenomenon.^66^ We were, however, intrigued to observe that treatment of HCT116 cells with **2** (10 μM) led to the stabilization of HIF-1α above the level observed in hypoxia alone (Figure 10D). While it has long been known that CREBBP/EP300 bind to HIF-1α,^9^ the mechanism by which this stabilization occurs is currently unclear. It has previously been shown that the inhibition of the BET bromodomains with (+)-JQ1 does not affect HIF expression levels or activity,^67^ indicating that **2** selectively inhibits the CREBBP/EP300 bromodomains in cells, resulting in this effect. This observation potentially offers a novel route for the intervention in clinical conditions affected by HIF-1α levels.

## Conclusions

In conclusion, we report the SAR studies that led to the development of a high affinity ligand for the bromodomains of CREBBP and EP300. A key aspect was the understanding that the intramolecular hydrogen bond present in **6** predisposed the molecule to adopt a solution phase conformation that is unfavorable for binding to the CREBBP/EP300 bromodomains. Replacement of the amide with a (*E*)-alkene led to the high affinity ligand **2** [(−)-OXFBD05]. This ligand does not show appreciable affinity at any of the bromodomains we evaluated, including BRD2(1) and BRD4(1), making it suitable for use in cellular studies. Initial work shows that inhibition of the CREBBP/EP300 bromodomain results in the downregulation of c-Myc in HCT116 colon cancer cells. A reduction in H3K18 and H3K27 acetylation is also observed, demonstrating that the bromodomain plays a role in the KAT function of CREBBP/EP300. In addition, stabilization of HIF-1α above the level observed in hypoxia alone was seen. (−)-OXFBD05 (**2**) and its inactive enantiomer companion compound (+)-OXFBD05 (**3**) are useful to the tools that exist for studying CREBBP and EP300. Beyond its specific focus, our study provides wider insight into the use of intramolecular hydrogen bonds to manipulate the solution state conformation of molecules. We have paid particular attention to comparing the presence of intramolecular hydrogen bonds in solution and when bound to the CREBBP bromodomain. This general approach potentially offers the ability to design compounds that have favorable entropic properties for protein binding while retaining useful physicochemical properties. Consequently, this work will be of broad interest to those engaged in the design of probe and drug molecules.

## Experimental Section

### Synthetic Chemistry

^1^H NMR spectra were recorded on a Bruker AVIIIHD 400 nanobay (400 MHz), Bruker AVII 500 (500 MHz) with dual ^13^C(^1^H) cryoprobe, or Bruker AVIIIHD 500 (500 MHz) spectrometer in the stated solvents as a reference for the internal deuterium lock. The chemical shift data for each signal are given as δ_H_ in units of parts per million (ppm) relative to tetramethylsilane (TMS) where δ_H_ (TMS) = 0.00 ppm. The spectra are calibrated using the solvent peak with the data provided by Fulmer *et al*.^68^ The multiplicity of each signal is indicated by s (singlet); br s (broad singlet); d (doublet); dd (doublet of doublets), ddd (doublet of doublet of doublets), t (triplet), q (quartet), qn (quintet), dq (double of quartet), or m (multiplet). The number of protons (n) for a given resonance signal is indicated by nH. Where appropriate, coupling constants (*J*) are quoted in Hz and are recorded to the nearest 0.1 Hz. Identical proton coupling constants (*J*) are averaged in each spectrum and reported to the nearest 0.1 Hz. The coupling constants are determined by analysis using Bruker TopSpin software. ^13^C NMR spectra were recorded on a Bruker AVIIIHD 400 nanobay (101 MHz) or Bruker AVII 500 (126 MHz) spectrometer, with a dual ^13^C(^1^H) cryoprobe in the stated solvents, with broadband proton decoupling and an internal deuterium lock. The chemical shift data for each signal are given as δ_C_ in units of parts per million (ppm) relative to tetramethylsilane (TMS) where δ_C_ (TMS) = 0.00 ppm. The spectra are calibrated using the solvent peak with the data reported by Fulmer *et al*.^68^ The shift values of resonances are quoted to 1 decimal place unless peaks have similar chemical shifts, in which case 2 decimal places are used. Coupling constants (*J*) are quoted in Hz and are recorded to the nearest 0.1 Hz. The coupling constants are determined by analysis using Bruker TopSpin software. ^19^F NMR spectra were recorded on a Bruker AVIIIHD 400 nanobay (376 MHz) using a deuterium internal lock. The chemical shift data for each signal are given as δ_F_ in units of parts per million (ppm). The multiplicity of each signal is indicated by s (singlet); d (doublet); t (triplet); q (quartet), or m (multiplet). Coupling constants (*J*) are quoted in Hz and are recorded to the nearest 0.1 Hz. The coupling constants are determined by analysis using Bruker TopSpin software. ^11^B NMR spectra were recorded on a Bruker AVIIIHD 500 (160 MHz). The chemical shift data for each signal are given as δ_B_ in units of parts per million (ppm). Coupling constants (*J*) are quoted in Hz and are recorded to the nearest 1 Hz. Identical coupling constants (*J*) are averaged in each spectrum and reported to the nearest 1 Hz. The coupling constants are determined by analysis using Bruker TopSpin software. Low-resolution mass spectra (LRMS) were recorded on a Waters LCT Premier spectrometer or Agilent 6120 Quadrupole LC/MS spectrometer (ESI). *m*/*z* values are reported in Daltons and followed by their percentage abundance in parentheses. High-resolution mass spectra (HRMS) were recorded on a Bruker MicroTOF spectrometer, operating in positive or negative mode, as indicated, from solutions of MeOH, MeCN, or H_2_O. *m*/*z* values are reported in Daltons and followed by their percentage abundance in parentheses. Electron ionization/field ionization (EI/FI) was carried out on a Waters GCT with a temperature-programmed solid probe inlet. When a compound was not observed by LRMS, only HRMS is quoted. Melting points on crystallized samples were determined using either (a) a Leica Galen III hot stage microscope or (b) a Griffin capillary tube melting point apparatus and are uncorrected. The solvents of crystallization are shown in parentheses. Infrared spectra were obtained from thin films using a diamond attenuated total reflectance module. The spectra were recorded on a Bruker Tensor 27 spectrometer. Absorption maxima (*ν̅*_max_) are reported in wavenumbers (cm^–1^) and are classified as broad (br), strong (s), medium (m), or weak (w). Analytical HPLC was carried out on a PerkinElmer Flexar system with a binary LC pump and UV/vis LC detector. For the determination of compound purity, the following methods were applied. Method 1 (M1): a Dionex Acclaim 120 column (C18, 5 μm, 120 Å, 4.6 × 150 mm) was used, and the solvents employed were A = 0.1% (*v*/*v*) solution of TFA in 95% H_2_O/5% MeCN; B = 0.1% (*v*/*v*) solution of TFA in 95% MeCN/5% H_2_O and the gradient (A:B). A 10 min linear gradient of 0–100% B was run with a flow rate of 1 mL/min and detection at 254 nm. Samples were injected in DMSO, MeOH, DMSO/MeOH, or DMSO/CHCl_3_. Method 2 (M2): a Dionex Acclaim 120 column (C18, 5 μm, 120 Å, 4.6 × 150 mm) was used, and the solvents employed were A = H_2_O and B = MeCN. Linear gradient conditions (0–10 min, linear increase from 5 to 95% of B; 10–15 min, B = 95%) with a flow rate of 1.5 mL/min and detection at 254 nm. Samples were injected in DMSO, MeOH, DMSO/MeOH, or DMSO/CHCl_3_. For the determination of enantio-purity, method 3 (M3), Daicel ChiralPak (AD-H, 5 μm, 120 Å, 4.6 × 250 mm), was used with gradient elution of isopropanol and hexane (50:50). Samples were injected in isopropanol. Chromera software was used to determine purity and enantiomeric excess from relative peak areas of UV/vis absorbance at 254 nm. All biologically tested samples had a purity of ≥95% as determined by HPLC analysis (HPLC traces are included in the SI).

Anhydrous solvents were obtained under the following conditions: Et_2_O, toluene, and CH_2_Cl_2_ were dried by passing through a column of activated basic alumina according to Grubbs’ procedure.^69^ Anhydrous 1,2-dichloroethane, DMF, DMSO, MeOH, and MeCN were purchased from Sigma Aldrich UK in SureSeal bottles and used without further purification. All other solvents were used as supplied (analytical or HPLC grade) without purification. Solvents used for cross coupling reactions were degassed with nitrogen for 30 min. Chemicals were purchased from Acros UK, Apollo Scientific, Enamine, Sigma Aldrich UK, Alfa Aesar UK, Fisher Scientific UK, Fluka UK, Fluorochem, Merck, Argo International Limited, and TCI-Europe. All reagents were purified, when necessary, by standard techniques.^70^ Zinc and iron metals were activated according to the Kishi’s procedure.^71^ Where appropriate and if not otherwise stated, all non-aqueous reactions were performed in a flame dried flask under an inert atmosphere.

#### Benzyl 2-Fluoro-3-nitrobenzoate (**17**)

To a suspension of 2-fluoro-3-nitrobenzoic acid (1.00 g, 5.40 mmol, 1.0 equiv), K_2_CO_3_ (1.12 g, 8.10 mmol, 1.5 equiv) in DMF (12.5 mL) was added benzyl bromide (1.00 mL, 8.10 mmol, 1.5 equiv). The suspension was stirred for 3 h at 60 °C, after which time, TLC analysis indicated that the reaction was complete. After cooling to rt, the yellow suspension was diluted with EtOAc (60 mL), washed with 2 M aq K_2_CO_3_ (3 × 60 mL) and brine (60 mL), dried (MgSO_4_), filtered, and concentrated *in vacuo*. The yellow oil was purified by silica gel column chromatography eluting with a gradient of 10 to 20% EtOAc in petroleum ether to give a yellow oil **17** (1.37 g, 93%): R_f_ 0.35 (petroleum ether:EtOAc 6:1); ^1^H NMR (400 MHz, CDCl_3_): δ 8.30–8.15 (2H, m), 7.50–7.34 (6H, m), 5.43 (2H, s); ^19^F NMR (377 MHz, CDCl_3_): δ −116.2 to −116.3 (1F, m); LRMS *m*/*z* (ES^+^) 298 ([M + Na]^+^, 100%). These data are in good agreement with the literature.^11^

### General Procedure for the Preparation of *N*-Benzyl-Protected Amines **10** and **11**

The appropriate *N*-benzyl-1-phenylethanamine (16.8 mmol, 1.5 equiv) in dry THF (20 mL) was cooled to −78 °C. To the colorless solution, *^n^*BuLi in hexane (2.5 M, 17.5 mmol, 1.6 equiv) was added slowly, over a period of 15 min at −78 °C. After the addition was complete, the pink solution was stirred for another 30 min at −78 °C. After this time, *tert*-butyl crotonate **9** (11.0 mmol, 1.0 equiv) in dry THF (15 mL) was added dropwise over a period of 45 min at −78 °C and stirred for a further 3 h. After this time, the reaction was judged to be complete by TLC analysis. The reaction was quenched with sat. aq NH_4_Cl (10 mL) and warmed to ambient temperature. The solvent was removed *in vacuo*, and the residue was taken up in 10% *v*/*v* aq citric acid (50 mL) and extracted with CH_2_Cl_2_ (3 × 50 mL). The combined organic extracts were washed with sat. aq NaHCO_3_ (100 mL) and brine (100 mL), dried (MgSO_4_), filtered, and concentrated *in vacuo*. The crude was purified using silica gel chromatography (petroleum ether:EtOAc gradient).

#### (*R*)-*tert*-Butyl 3-(benzyl((R)-1-phenylethyl)amino)butanoate (**10**)

From (*R*)-*N*-benzyl-1-phenylethanamine, to yield a pale yellow oil (3.27 g, 84%): R_f_ 0.61 (petroleum ether:EtOAc 10:1); [α]_*D*_^25^ = −4.9 (*c* 1.1, CHCl_3_) [Lit:^72^ [α]_*D*_^25^ = −5.2 (*c* 1.1, CHCl_3_)]; ^1^H NMR (400 MHz, CDCl_3_): δ 7.43–7.16 (10H, m), 3.88 (1H, q, *J* 6.9), 3.75 (1H, d, *J* 15.0), 3.60 (1H, d, *J* 15.0), 3.46–3.36 (1H, m), 2.24 (1H, dd, *J* 14.1, 4.7), 2.24 (1H, dd, *J* 14.1, 9.2), 1.38 (9H, s), 1.32 (3H, d, *J* 6.9), 1.10 (3H, d, *J* 6.7); LRMS *m*/*z* (ES^+^) 354 ([M + H]^+^, 100%). These data are in accordance with the literature.^72^

#### (*S*)-*tert*-Butyl 3-(benzyl((*S*)-1-phenylethyl)amino)butanoate (**11**)

From (*S*)-*N*-benzyl-1-phenylethanamine, a colorless oil (2.67 g, 69%): R_f_ 0.68 (petroleum ether:EtOAc 10:1); [α]_*D*_^25^ = +3.9 (*c* 1.1, CHCl_3_), [Lit:^72^ [α]_*D*_^25^ = +5.1 (*c* 1.1, CHCl_3_)]; other physical and spectroscopic properties are identical to those of **10**. These data are in agreement with the literature.^72^

#### *tert*-Butyl 3-(benzylamino)-2-methylpropanoate (**15**)

Benzylamine (1.26 mL, 11.6 mmol, 1.1 equiv) and *tert*-butyl methacrylate **14** (1.50 g, 10.5 mmol, 1.0 equiv) were dissolved in 1,8-diazabicyclo[5.4.0]undec-7-ene (2.41 g, 15.8 mmol, 1.5 equiv) and stirred at 90 °C for 16 h, after which time, TLC analysis indicated that the reaction was complete. After cooling to ambient temperature, the DBU was evaporated at 40 °C at 120 mbar. The crude material was purified using silica gel chromatography, eluting with a gradient of 10 to 20% EtOAc:petroleum ether, to obtain **15** as a colorless oil (1.34 g, 5.37 mmol, 51%). R_f_ 0.37 (petroleum ether:EtOAc 5:1); ^1^H NMR (500 MHz, CDCl_3_): δ 7.35–7.28 (4H, m), 7.27–7.21 (1H, m), 3.80 (1H, d, *J* 16.5), 3.77 (1H, d, *J* 16.5), 2.85 (1H, dd, *J* 11.5, 7.9) 2.65–2.53 (2H, m), 1.44 (9H, s), 1.13 (3H, d, *J* 7.0), ^13^C NMR (126 MHz, CDCl_3_): δ 175.3, 140.4, 128.4, 128.1, 126.9, 80.3, 53.8, 52.4, 40.9, 28.1, 15.4; LRMS *m*/*z* (ES^+^) 250 ([M + H]^+^, 100%); HRMS *m*/*z* (ES^+^) [found: (M + H)^+^ 250.1798, C_24_H_31_O_3_N_4_^+^ requires 250.1802].

### General Procedure for the Deprotection of *N*-Benzyl Amines to Give **12** and **13**

To a solution of the appropriate *N*-benzyl-protected amine (5.60 mmol, 1.0 equiv) in H_2_O (3.2 mL), glacial acetic acid (2.0 mL), and MeOH (80 mL) was added 20% Pd(OH)_2_/C (1.14 mmol, 0.2 equiv). The system was purged with nitrogen, and then hydrogen gas was added. The suspension was stirred for 15 h under an atmosphere of hydrogen at ambient temperature. The black suspension was filtered through Celite, dried (MgSO_4_), filtered, and concentrated *in vacuo*.

#### (*R*)-*tert*-Butyl 3-Aminobutanoate (**12**)

From **10**, to yield a yellow oil containing 1.9 equiv acetic acid (1.40 g, 91%), an aliquot was purified for further characterization: R_f_ 0.22 (EtOAc:Et_3_N 10:1); [α]_*D*_^25^ = −18.6 (*c* 0.5, CHCl_3_), [Lit:^72^ [α]_*D*_^25^ = −22.2 (*c* 0.5, CHCl_3_)]; ^1^H NMR (400 MHz, CDCl_3_) δ 3.38–3.27 (1H, m), 2.32 (1H, dd, *J* 15.4, 4.7), 2.20 (1H, dd, *J* 15.4, 8.5), 1.45 (9H, s), 1.10 (3H, d, *J* 6.5); LRMS *m*/*z* (ES^+^) 160 ([M + H]^+^, 100%). These data are in agreement with the literature.^72^

#### (*S*)-*tert*-Butyl 3-Aminobutanoate (**13**)

From **11**, to yield a yellow oil (706 mg, 79%): R_f_ 0.22 (EtOAc:Et_3_N 10:1); [α]_*D*_^25^ = +20.2 (*c* 0.5, CHCl_3_), [Lit:^72^ [α]_*D*_^25^ = +21.4 (*c* 0.6 CHCl_3_)]; other physical and spectroscopic properties are identical to those of **12**. These data are in agreement with the literature.^72^

### General Procedure for the Preparation of anilines **18**, **19**, and **22**

For **22**, *tert*-butyl 3-(benzylamino)-2-methylpropanoate **15** (5.90 mmol, 1.0 equiv) was dissolved in H_2_O (3.2 mL), glacial acetic acid (2.0 mL), and MeOH (80 mL) and was added 20% Pd(OH)_2_/C (1.14 mmol, 0.2 equiv). The system was purged with nitrogen, and then hydrogen gas was added. The suspension was stirred for 15 h under an atmosphere of hydrogen at ambient temperature. The black suspension was filtered over Celite, dried (MgSO_4_), filtered, and concentrated *in vacuo* to yield **16** as a yellow oil, which was used without further purification. Benzyl 2-fluoro-3-nitrobenzoate **17** (4.66 mmol, 1.0 equiv) was added to the appropriate amine (5.12 mmol, 1.1 equiv), Cs_2_CO_3_ (18.6 mmol, 4.0 equiv) suspended in toluene (80 mL). The yellow suspension was stirred at 85 °C for 14 h at which time TLC analysis indicated the reaction to be complete. After cooling to ambient temperature, the reaction mixture was diluted with EtOAc (200 mL), washed with 2 M aq K_2_CO_3_ (3 × 150 mL), dried (Na_2_SO_4_), filtered, and concentrated *in vacuo*. The crude was purified by silica gel chromatography (petroleum ether:EtOAc gradient).

#### (*R*)-Benzyl 2-(4-*tert*-Butoxy-4-oxobutan-2-ylamino)-3-nitrobenzoate (**18**)

From **12**, to yield a yellow oil (1.87 g, 97%): R_f_ 0.34 (petroleum ether:EtOAc 10:1); [α]_*D*_^25^ = +64.4 (*c* 1.0, CHCl_3_); *ν̅*_max_ (thin film)/cm^–1^ 3308 (w), 2978 (m), 1724 (s), 1691 (s), 1604 (m), 1586 (m), 1530 (m), 1499 (m), 1455 (m), 1367 (m), 1348 (m), 1248 (s); ^1^H NMR (500 MHz, CDCl_3_) δ 8.19 (1H, d, *J* 9.6), 8.04 (1H, dd, *J* 8.0, 1.6), 7.94 (1H, dd, *J* 8.0, 1.6), 7.46–7.33 (5H, m), 6.70 (1H, dd, *J* 8.0, 8.0), 5.35 (2H, s), 3.68–3.59 (1H, m), 2.41 (1H, dd, *J* 15.0, 6.2), 2.39 (1H, dd, *J* 15.0, 6.2), 1.33 (9H, s), 1.25 (3H, d, *J* 6.2); ^13^C NMR (126 MHz, CDCl_3_) δ 170.0, 167.1, 144.6, 138.4, 136.8, 135.4, 131.2, 128.7, 128.6, 128.4, 118.3, 115.5, 80.9, 67.2, 50.0, 43.7, 27.9, 20.9; LRMS *m*/*z* (ES^+^) 415 ([M + H]^+^, 100%); HRMS *m*/*z* (ES^+^) [found: (M + H)^+^ 415.1851, C_22_H_27_O_6_N_2_^+^, requires 415.1864]. RP-HPLC: method A: retention time 15.49 min, purity 97.8%.

#### (*S*)-Benzyl 2-(4-*tert*-Butoxy-4-oxobutan-2-ylamino)-3-nitrobenzoate (**19**)

From **13**, to yield a yellow oil (790 mg, 83%): [α]_*D*_^25^ = −65.6 (*c* 0.5, CHCl_3_), other physical and spectroscopic properties are identical to those of **18**. RP-HPLC: method A: retention time 15.49 min, purity 99.4%.

#### Benzyl 2-(3-*tert*-Butoxy-2-methyl-3-oxopropylamino)-3-nitrobenzoate (**22**)

From **15**, to yield a yellow oil (1.36 g, 96% over 2 steps): R_f_ 0.35 (EtOAc:petroleum ether 1:10); *ν̅*_max_ (thin film)/cm^–1^ 3212 (w), 2977 (w), 2936 (w), 1724 (m), 1690 (m), 1583 (s), 1530 (s), 1500 (s), 1367 (m), 1153 (m), 1107 (m); ^1^H NMR (500 MHz, CDCl_3_) δ 8.55 (1H, t, *J* 4.7), 8.08 (1H, dd, *J* 8.0, 1.7), 7.99 (1H, dd, *J* 8.0, 1.7), 7.45–7.32 (5H, m), 6.67 (1H, dd, *J* 8.0, 8.0), 5.34 (2H, s), 3.11–2.99 (2H, m), 2.67–2.58 (1H, m), 1.36 (9H, s), 1.13 (3H, d, *J* 7.2), ^13^C NMR (126 MHz, CDCl_3_) δ 173.5, 166.9, 145.9, 137.14, 137.05, 135.4, 131.6, 128.7, 128.6, 128.4, 117.0, 114.6, 81.1, 67.1, 50.3, 41.3, 27.9, 14.9; LRMS *m*/*z* (ES^+^) 415 ([M + H]^+^, 100%), 437 ([M + Na]^+^, 93%); HRMS *m*/*z* (ES^+^) [found: (M + Na)^+^ 437.1681, C_22_H_26_O_6_N_2_Na^+^ requires 437.1683]. HPLC: method: A: retention time 15.67 min, purity 99.6%.

### General Procedure for the Preparation of 4,5-Dihydrobenzodiazepinones **20** and **21**

To a solution of appropriate aniline (3.11 mmol 1.0 equiv) in CH_2_Cl_2_ (10 mL) was added trifluoroacetic acid (10 mL), and the orange solution was stirred at rt for 1.5 h. After this time, the solvent was evaporated *in vacuo* and the resulting residue was dissolved in glacial acetic acid (30 mL). Iron powder (15.6 mmol, 5.0 equiv) was added, and the brown suspension was heated under reflux for 4 h. After this time, 1 M aq K_2_CO_3_ (300 mL) was added to the cooled suspension and the resulting mixture was extracted with EtOAc (3 × 200 mL). The combined organic components were washed with 0.5 M aq LiCl (3 × 100 mL) and brine (150 mL), dried (MgSO_4_), filtered, and evaporated *in vacuo*. The crude was purified using silica gel chromatography (petroleum ether:EtOAc gradient).

#### (*R*)-Benzyl 4-Methyl-2-oxo-2,3,4,5-tetrahydro-1*H*-benzo[b][1,4]diazepine-6-carboxylate (**20**)

From **18**, to yield a pale yellow solid (632 mg, 65%): R_f_ 0.44 (petroleum ether:EtOAc 11:1); mp 133–134 °C (CHCl_3_), [α]_*D*_^25^ = −44.2 (*c* 1.1, CHCl_3_); *ν̅*_max_ (thin film)/cm^–1^ 3309 (w), 3219 (w), 2961 (w), 1665 (s), 1531 (m), 1497 (m), 1471 (m), 1449 (m), 1265 (s), 1233 (s); ^1^H NMR (500 MHz, CDCl_3_) δ 8.42 (1H, br s), 8.00 (1H, br s), 7.83 (1H, dd, *J* 7.9, 1.6), 7.45–7.32 (5H, m), 7.04 (1H, dd, *J* 7.9, 1.6), 6.70 (1H, t, *J* 7.9), 5.32 (2H, s), 4.11–4.03 (1H, m), 2.68 (1H, dd, *J* 14.1, 2.6), 2.58 (1H, dd, *J* 14.1, 8.6), 1.39 (3H, d, *J* 6.4); ^13^C NMR (126 MHz, CDCl_3_) δ 173.0, 168.3, 142.9, 135.8, 128.61, 128.57, 128.3, 128.0, 127.2, 126.1, 116.9, 114.8, 66.7, 51.4, 42.5, 23.9; LRMS *m*/*z* (ES^+^) 311 ([M + H]^+^, 100%); HRMS *m*/*z* (ES^+^) [found: (M + H)^+^ 311.1387, C_18_H_19_O_3_N_2_^+^ requires 311.1390]. RP-HPLC: method A: retention time 12.47 min, purity 99.1%.

#### (*S*)-Benzyl 4-Methyl-2-oxo-2,3,4,5-tetrahydro-1*H*-benzo[b][1,4]diazepine-6-carboxylate (**21**)

From **19**, to yield a pale yellow solid (327 mg, 89%): [α]_*D*_^25^ = +38.2 (*c* 0.5, CHCl_3_); other physical and spectroscopic properties are identical to those of **20**. RP-HPLC: method A: retention time 12.45 min, purity 99.5%.

#### Benzyl 3-Methyl-2-oxo-2,3,4,5-tetrahydro-1*H*-benzo[b][1,4]diazepine-6-carboxylate (**23**)

Benzyl 2-(3-*tert*-butoxy-2-methyl-3-oxopropylamino)-3-nitrobenzoate **22** (1.00 g, 2.41 mmol, 1.0 equiv) was dissolved in trifluoroacetic acid (10 mL) and dichloromethane (10 mL) and stirred for 2 h, at ambient temperature. The solvent was concentrated *in vacuo* to afford an orange oil. Zn (3.96 g, 60.6 mmol, 25 equiv), NH_4_Cl (3.24 g, 60.6 mmol, 25 equiv), and the residue were suspended in DMF (48 mL) and stirred for 16 h at ambient temperature. After this time, the reaction was judged to be complete by TLC analysis. The suspension was filtered through Celite and washed with DMF (4 mL). The pH of the filtrate was adjusted to pH 9 with Et_3_N (2.5 mL). To the filtrate, PyBOP (1.25 g, 2.41 mmol, 1.0 equiv) was added and stirred for 20 h at ambient temperature. After this time, the reaction was judged to be complete by TLC analysis. The reaction mixture was diluted with EtOAc (150 mL), washed with brine (3 × 200 mL), dried (MgSO_4_), filtered, and concentrated *in vacuo*. The crude material was purified using silica gel chromatography, eluting with a gradient of 30 to 50% EtOAc in petroleum ether, to give **23** as a yellow solid. This compound was crystallized from EtOH to give a yellow solid (72 mg, 10% over 3 steps): R_f_ 0.46 (EtOAc:petroleum ether 1:1); *ν̅*_max_ (thin film)/cm^–1^ 1673 (s), 1538 (w), 1422 (w), 1257 (s), 1234 (s), 1102 (s); ^1^H NMR (500 MHz, CDCl_3_) δ 8.25 (1H, t, *J* 3.7), 7.82 (1H, dd, *J* 7.9, 1.5), 7.73 (1H, br s), 7.46–7.32 (5H, m), 6.97 (1H, dd, *J* 7.9, 1.5), 6.62 (1H, dd, *J* 7.9, 7.9), 5.32 (2H, s), 3.60–3.50 (2H, m), 2.93–2.83 (1H, m), 1.21 (3H, d, *J* 7.0); ^13^C NMR (126 MHz, CDCl_3_): δ 176.0, 168.4, 143.7, 136.0, 128.7, 128.5, 128.3, 128.1, 127.1, 125.5, 116.2, 113.8, 66.6, 50.5, 39.0, 13.7; LRMS *m*/*z* (ES^+^) 311 ([M + H]^+^, 100%); HRMS *m*/*z* (ES^+^) [found: (M + H)^+^ 311.1390, C_18_H_19_O_3_N_2_^+^ requires 311.1390]. RP-HPLC: method A: retention time 12.86 min, purity 97.8%.

### General Procedure for the Coupling of Carboxylic Acids to Amines to Give Amides **6**–**8**

To the appropriate 4,5-dihydrobenzodiazepinone (0.290 mmol, 1.0 equiv) in MeOH (10 mL) was added 10% Pd/C (15.4 mg, 0.029 mmol, 0.1 equiv). The system was purged with nitrogen, and then hydrogen gas was added. The suspension was stirred for 16 h under an atmosphere of hydrogen at ambient temperature. The black suspension was filtered through Celite and washed with MeOH (2 mL). The filtrate was dried (MgSO_4_), filtered, and concentrated *in vacuo* to give a crude solid. To a solution of the appropriate carboxylic acid (0.290 mmol, 1.0 equiv) and PyBOP (0.319 mmol, 1.1 equiv) in DMF (1.5 mL) was added triethylamine (80 μL, 0.58 mmol, 2.0 equiv). The reaction mixture was stirred for 10 min, then a solution of 3-(7-methoxy-3,4-dihydroquinolin-1(2*H*)-yl)propan-1-amine **24** (0.29 mmol, 1.1 equiv) in DMF (1.5 mL) was added, and stirring continued for 19 h. The reaction mixture was diluted with EtOAc (50 mL), washed with H_2_O (3 × 50 mL) and brine (50 mL), dried (MgSO_4_), filtered, and concentrated *in vacuo*. The crude was purified using silica gel chromatography (petroleum ether:EtOAc gradient).

#### (*R*)-3-(7-Methoxy-3,4-dihydroquinolin-1(2*H*)-yl)propyl-4-methyl-2-oxo-2,3,4,5-tetrahydro-1*H*-benzo[b][1,4]diazepine-6-carboxylate (**6**)

From **20**, to yield a colorless solid (82 mg, 68%): R_f_ 0.41 (EtOAc:petroleum ether 3:1); mp 157–158 °C (CHCl_3_), [α]_*D*_^25^ = −21.0 (*c* 0.5, CHCl_3_); *ν̅*_max_ (thin film)/cm^–1^ 3310 (w), 3213 (w), 3069 (w), 2941 (m), 1738 (s), 1692 (s), 1613 (s), 1542 (m), 1508 (s), 1239 (s), 1167 (s); ^1^H NMR (500 MHz, CDCl_3_) δ 7.77 (1H, br s), 7.30 (1H, br s), 7.04 (1H, dd, *J* 7.8, 1.4), 6.91 (1H, d, *J* 7.8), 6.86 (1H, d, *J* 7.8), 6.68 (1H, dd, *J* 7.8, 7.8), 6.41 (1H, t, *J* 5.5), 6.20–6.13 (2H, m), 4.07–3.96 (1H, m), 3.74 (3H, s), 3.56–3.44 (2H, m) 3.36 (2H, t, *J* 6.9), 3.25 (2H, dd, *J* 5.6, 5.6), 2.70–2.60 (3H, m), 2.48 (1H, dd, *J* 14.0, 8.0), 1.98–1.85 (4H, m), 1.33 (3H, d, *J* 6.3); ^13^C NMR (126 MHz, CDCl_3_) δ 172.7, 169.5, 159.2, 145.9, 140.4, 129.7, 128.0, 124.8, 124.1, 122.3, 118.3, 115.7, 99.9, 97.7, 55.2, 52.5, 49.6, 49.5, 41.8, 38.3, 27.2, 26.4, 23.7, 22.4; LRMS *m*/*z* (ES^+^) 423 ([M + H]^+^, 100%); HRMS *m*/*z* (ES^+^) [found: (M + H)^+^ 423.2385. C_24_H_31_O_3_N_4_^+^ requires 423.2391]. RP-HPLC: method A: retention time 9.88 min, purity 96.0%; chiral HPLC: retention time 14.47 min, ee >99%.

#### (*S*)-3-(7-Methoxy-3,4-dihydroquinolin-1(2*H*)-yl)propyl-4-methyl-2-oxo-2,3,4,5-tetrahydro-1*H*-benzo[b][1,4]diazepine-6-carboxylate (**7**)

From **21**, to yield a pale yellow solid (19 mg, 26%): R_f_ 0.41 (EtOAc:petroleum ether 3:1); [α]_*D*_^25^ = +21.4 (*c* 0.5, CHCl_3_); other physical and spectroscopic properties identical to **6**, RP-HPLC: method A: retention time 9.80 min, purity 97.1%; chiral HPLC: retention time 11.97 min, ee >90%.

#### *N*-(3-(7-Methoxy-3,4-dihydroquinolin-1(2*H*)-yl)propyl)-3-methyl-2-oxo-2,3,4,5-tetrahydro-1*H*-benzo[b][1,4]diazepine-6-carboxamide (**8**)

From **23**, to yield a pale yellow solid (23 mg, 34%): R_f_ 0.37 (EtOAc:petroleum ether 3:1); *ν̅*_max_ (thin film)/cm^–1^ 3307 (w, br), 2934 (m), 1738 (s), 1669 (s), 1613 (s), 1508 (s), 1282 (s), 1167 (m); ^1^H NMR (500 MHz, CDCl_3_) δ 7.55 (1H, br s), 7.38 (1H, s), 7.03, (1H, dd, *J* 7.8, 1.4), 6.88–6.85 (1H, m), 6.86 (1H, dd, *J* 7.7, 1.4), 6.62 (1H, dd, *J* 7.7, 7.7), 6.45 (1H, t, *J* 4.7), 6.20–6.15 (2H, m), 3.74 (3H, s), 3.55–3.44 (4H, m), 3.36 (2H, t, *J* 6.9), 3.28–3.24 (2H, m), 2.89–2.80 (1H, m), 2.67 (2H, t, *J* 6.4), 1.97–1.86 (4H, m), 1.18 (3H, d, *J* 5.9) ^13^C NMR (126 MHz, CDCl_3_) δ 175.9, 169.5, 159.3, 145.9, 141.2, 129.8, 127.0, 125.0, 124.0, 120.8, 117.4, 115.8, 100.1, 97.8, 55.2, 51.6, 49.6, 38.5, 38.3, 27.3, 26.4, 22.4, 13.7; LRMS *m*/*z* (ES^+^) 423 ([M + H]^+^, 100%); HRMS *m*/*z* (ES^+^) [found: (M + H)^+^ 423.2382, C_24_H_31_O_3_N_4_^+^ requires 423.2391]. RP-HPLC: method A: retention time 11.74 min, purity 98.3%.

### General Procedure for the Preparation of Anilines **29** and **30**

3-Bromo-2-fluoro-3-nitrobenzoate **28** (13.6 mmol, 1.0 equiv), the appropriate amine (18.4 mmol, 1.3 equiv), and DIPEA (78.9 mmol, 5.8 equiv) dissolved in DMF (50 mL) were stirred at 70 °C for 16 h. After this time, the reaction was judged to be complete by TLC analysis. Upon cooling to ambient temperature, the solution was diluted with EtOAc (150 mL), washed with 0.5 M aq LiCl (3 × 100 mL) and brine (100 mL), dried (Na_2_SO_4_), filtered, and concentrated *in vacuo*.

#### *tert*-Butyl (*R*)-3-((2-Bromo-6-nitrophenyl)amino)butanoate (**29**)

From **12**, to yield a yellow oil (4.70 g, 96%), which was used without further purification: R_f_ 0.62 (petroleum ether:EtOAc 4:1); [α]_*D*_^25^ −74.3 (*c* 1.0 in CHCl_3_); *ν̅*_max_ (thin film)/cm^–1^ 3355 (w), 2379 (m), 2932 (w), 1725 (s), 1530 (m), 1480 (m), 1367 (m), 1259 (m), 1155 (s); ^1^H NMR (400 MHz, (CDCl_3_) δ 7.87 (1H, dd, *J* 8.3, 1.6), 7.66 (1H, dd, *J* 7.8, 1.6), 6.73 (1H, dd, *J* 8.3, 7.8), 6.30 (1H, d, *J* 10.2), 4.19 (1H, dq, *J* 10.2, 6.4), 2.40 (2H, d, *J* 6.1), 1.33 (9H, s), 1.22 (3H, d, *J* 6.4); ^13^C NMR (126 MHz, CDCl_3_): δ 170.3, 142.5, 141.0, 139.6, 125.7, 120.1, 116.5, 81.1, 50.1, 43.6, 28.0, 21.3; GC-HRMS *m*/*z* (CI^+^) [found: (M + H)^+^ 359.0603 C_14_H_19_^79^BrN_2_O_4_ requires 359.0601], RP-HPLC: method A: retention time 15.2 min, purity 92.6%.

#### *tert*-Butyl (*S*)-3-((2-Bromo-6-nitrophenyl)amino)butanoate (**30**)

From **13**, to yield a yellow oil (0.63 g, 82%), which was used without further purification: R_f_ 0.62 (EtOAc:petroleum ether 4:1); [α]_*D*_^25^ = +83.6 (*c* 1.0, CHCl_3_); other physical and spectroscopic properties identical to **29**.

### General Procedure for the Preparation of 4,5-Dihydrobenzodiazepinones **31** and **32**

To a solution of the appropriate aniline (3.11 mmol 1.0 equiv) in CH_2_Cl_2_ (10 mL) was added trifluoroacetic acid (10 mL), and the resulting orange solution was stirred at rt for 1.5 h. The reaction mixture was evaporated *in vacuo*, and the resulting residue was dissolved in glacial acetic acid (30 mL). Iron powder (15.6 mmol, 5.0 equiv) was added, and the brown suspension was heated under reflux for 4 h. After this time, 1 M aq K_2_CO_3_ (300 mL) was added to the cooled suspension and the resulting mixture was extracted with EtOAc (3 × 200 mL). The combined organic components were washed with 0.5 M aq LiCl (3 × 100 mL) and brine (150 mL), dried (MgSO_4_), filtered, and evaporated *in vacuo*. The crude was purified using silica gel chromatography (petroleum ether:EtOAc gradient).

#### (*R*)-6-Bromo-4-methyl-1,3,4,5-tetrahydro-2*H*-benzo[b][1,4]diazepin-2-one (**31**)

From **29**, to yield a pale yellow solid (2.38 g, 83%): R_f_ 0.63 (petroleum ether:EtOAc 2:1); mp: 164–166 °C (IPA); [α]_*D*_^25^ −6.3 (*c* 1.0 in CHCl_3_); *ν̅*_max_ (thin film)/cm^–1^ 3314 (w), 3181 (w), 2987 (w), 1661 (s), 1435 (m), 1393 (m), 1377 (m), 764 (m), ^1^H NMR (500 MHz, CDCl_3_): δ 8.59 (1H, s), 7.31 (1H, dd, *J* 8.0, 1.4), 6.90 (1H, dd, *J* 8.0, 1.4), 6.72 (1H, dd, *J* 8.0, 8.0), 4.19–4.08 (2H, m) 2.61 (1H, dd, *J* 13.7, 3.8), 2.45 (1H, dd, *J* 13.7, 8.2), 1.38 (3H, d, *J* 6.3); ^13^C NMR (126 MHz, CDCl_3_): δ 173.4, 136.7, 129.2, 121.8, 121.4, 116.0, 54.7, 41.5, 24.1; LRMS *m*/*z* (ES^+^) 277 ([M^79^Br + Na]^+^, 91%), 279 ([M^81^Br + Na]^+^, 100%), HRMS *m*/*z* (EI^+^) [found: (M^79^Br + H)^+^ 255.0129 C_10_H_12_^79^BrN_2_O requires 255.0129], RP-HPLC: method A: retention time 10.52 min, purity 96.7%, chiral HPLC: retention time 10.3 min, ee 94%.

#### (*S*)-6-Bromo-4-methyl-1,3,4,5-tetrahydro-2*H*-benzo[b][1,4]diazepin-2-one (**32**)

From **30**, to yield a pale yellow solid (0.33 g, 75%): R_f_ 0.35 (EtOAc:petroleum ether 2:1); [α]_*D*_^25^ = +4.8 (*c* 1.0, CHCl_3_); other physical and spectroscopic properties are identical to **31**. Chiral HPLC: retention time 5.4 min, ee 96%.

#### 7-Methoxy-1-(pent-4-yn-1-yl)-1,2,3,4-tetrahydroquinoline (**34**)

Pent-4-yn-1-yl methanesulfonate (738 mg, 4.56 mmol, 1.5 equiv) was added to a solution of methoxy-1,2,3,4-tetrahydroquinoline **33** (500 mg, 1.84 mmol, 1.0 equiv), potassium iodide (252 mg, 1.52 mmol, 0.5 equiv), and *N*,*N*-diisopropylethylamine (1.05 mL, 6.07 mmol, 2.0 equiv) in DMF (11 mL). The reaction mixture was sealed and heated to 100 °C in a microwave for 1 h. The cooled orange solution was diluted with Et_2_O (75 mL) and washed with H_2_O (3 × 75 mL) and brine (50 mL), dried (MgSO_4_), filtered, and evaporated *in vacuo* to afford an orange oil. The crude material was purified by silica gel chromatography, eluting with a gradient of 0 to 20% EtOAc:petroleum ether, to obtain **34** as a colorless oil (545 mg, 78%): R_f_ 0.68 (petroleum ether:EtOAc 1:1) *ν̅*_max_ (thin film)/cm^–1^ 3284 (w), 2933 (w), 1610 (s), 1507 (m), 1242 (m), 1162 (m), 819 (m), ^1^H NMR (400 MHz, CDCl_3_): δ 6.84 (1H, d, *J* 8.2), 6.20–6.10 (2H, m), 3.76 (3H, s), 3.39–3.31 (2H, m), 3.31–3.24 (2H, m), 2.69 (2H, t, *J* 8.2), 2.26 (2H, td, *J* 6.9, 2.6), 2.00 (1H, t, *J* 2.6), 1.97–1.87 (2H, m), 1.82 (2H, m); ^13^C NMR (101 MHz, CDCl_3_): δ 159.3, 146.0, 129.6, 115.2, 100.1, 97.4, 83.9, 69.0, 55.2, 50.3, 49.7, 27.4, 25.1, 22.5, 16.1; HRMS *m*/*z* (ES^+^) [found: (M + H)^+^ 230.15382, C_10_H_12_NO_2_^+^ requires 230.15394]; RP-HPLC: method B: retention time 17.11 min, purity 98.5%.

#### *(E*)-7-Methoxy-1-(5-(4,4,5,5-tetramethyl-1,3,2-dioxaborolan-2-yl)pent-4-en-1-yl)-1,2,3,4-tetrahydroquinoline (**35**)

7-Methoxy-1-(pent-4-yn-1-yl)-1,2,3,4-tetrahydroquinoline **34** (300 mg, 1.38 mmol, 1.0 equiv) and bis(cyclopentadienyl)zirconium(IV) chloride hydride (33 mg, 0.013 mmol, 0.1 equiv) were added to a tapered microwave vial and sealed under an argon atmosphere (evacuated and backfilled 3 × argon). Triethylamine (18 μL, 0.013 mmol, 0.1 equiv), 1,2-dichloroethane (0.6 mL), and 4,4,5,5-tetramethyl-1,3,2-dioxaborolane (208 μL, 1.44 mmol, 1.1 equiv) was added, and the reaction mixture was stirred at 60 °C under argon for 20 h in the absence of light. The reaction mixture was purified by silica gel chromatography (elution with 0% to 100% CH_2_Cl_2_ in petroleum ether) to yield **35** as a colorless oil (340 mg, 72%) and was used without further purification: R_f_ 0.63 (CH_2_Cl_2_); *ν̅*_max_ (thin film)/cm^–1^ 2975 (w br), 2930 (w), 1608 (s), 1509 (m), 1141 (m), 863 (m), ^1^H NMR (400 MHz, CDCl_3_): δ 6.82 (1H, d, *J* 8.7), 6.65 (1H, dt, *J* 18.0, 6.4), 6.16–6.02 (2H, m), 5.47 (1H, dt, *J* 18.0, 1.6), 3.28–3.18 (4H, m), 2.67 (2H, t, *J* 6.3), 2.20 (2H, m), 1.90 (2H, m), 1.78–1.69 (2H, m), 1.26 (12H, s); ^11^B NMR (160 MHz; CDCl_3_) δ 29.7 (B-1″); ^13^C NMR (101 MHz, CDCl_3_): δ 159.2, 153.6, 146.1, 129.5, 115.1, 99.8, 97.2, 83.1, 55.2, 51.0, 49.4, 33.3, 27.5, 24.8, 22.5; LRMS *m*/*z* (ES^+^) 358.2 ([M + H]^+^, 100%); HRMS *m*/*z* (ES^+^) [found: (M + H)^+^ 358.25479, C_21_H_33_NO_3_ requires 358.25480].

### General Procedure for Suzuki Cross-Coupling to Give 4,5-Dihydrobenzodiazepinones **2** and **3**

The appropriate 4,5-dihydrobenzodiazepinone (0.290 mmol, 1.0 equiv), bis(triphenylphosphine)palladium(II) dichloride (0.014 mmol, 0.05 equiv) and K_2_CO_3_ (0.88 mmol, 3.0 equiv) were added to a Schlenk flask under a nitrogen atmosphere (evacuated and backfilled 3 × nitrogen). (*E*)-1-(5-(4,4,5,5-Tetramethyl-1,3,2-dioxaborolan-2-yl)pent-4-en-1-yl)-7-((triisopropylsilyl)oxy)-1,2,3,4-tetrahydroquinoline **35** (0.615 mmol, 1.5 equiv) in 1,4-dioxane (7 mL) was added, followed by H_2_O (1.75 mL), and the reaction mixture was stirred at 100 °C for 24 h. After this time, the reaction mixture was evaporated *in vacuo* and diluted with EtOAc (50 mL). The organic component was washed with brine (3 × 20 mL), dried (MgSO_4_), filtered, and evaporated *in vacuo*. The crude material was purified using silica gel chromatography (petroleum ether:EtOAc gradient).

#### (*R*,*E*)-6-(5-(7-Methoxy-3,4-dihydroquinolin-1(2*H*)-yl)pent-1-en-1-yl)-4-methyl-1,3,4,5-tetrahydro-2*H*-benzo[b][1,4]diazepin-2-one (**2**)

From **31**, to yield a colorless oil (105 mg, 89%): R_f_ 0.15 (petroleum ether:EtOAc 1:1); [α]_*D*_^25^ = −3.7 (*c* 1.0, CHCl_3_); *ν̅*_max_ (thin film)/cm^–1^ 3650 (w), 3382 (w), 2980 (s), 2360 (w), 1669 (s), 1508 (m), 1158 (m), 733 (s); ^1^H NMR (400 MHz, CDCl_3_): δ 8.65 (1H, s), 7.15–7.09 (1H, m), 6.90–6.84 (3H, m), 6.49 (1H, d, *J* 15.6), 6.18–6.04 (3H, m), 4.17–4.06 (1H, m), 3.76 (3H, s), 3.55 (1H, s), 3.33–3.23 (4H, m), 2.70 (2H, t, *J* 6.3), 2.62 (1H, dd, *J* 13.3, 4.7), 2.40 (1H, dd, *J* 13.3, 7.5), 2.33–2.26 (2H, m), 1.97–1.89 (2H, m), 1.84–1.74 (2H, m), 1.36 (3H, d, *J* 6.2); ^13^C NMR (101 MHz, CDCl_3_): δ 173.4, 159.2, 146.1, 135.8, 134.7, 130.8, 130.2, 129.5, 125.7, 124.2, 121.5, 121.1, 115.3, 99.4, 97.5, 55.6, 55.2, 51.0, 49.5, 40.8, 31.1, 27.5, 26.0, 24.1, 22.5; LRMS *m*/*z* (ES^+^) 406.2 ([M + H]^+^, 100%); HRMS *m*/*z* (ES^+^) [found: (M + H)^+^ 406.2498. C_25_H_32_N_3_O_2_ requires 406.2495]; RP-HPLC: method B: retention time 11.34 min, purity 99.7%; chiral HPLC: retention time 20.87 min, ee 94%.

#### (*S*,*E*)-6-(5-(7-Methoxy-3,4-dihydroquinolin-1(2*H*)-yl)pent-1-en-1-yl)-4-methyl-1,3,4,5-tetrahydro-2*H*-benzo[b][1,4]diazepin-2-one (**3**)

From **32**, to yield a colorless oil (77 mg, 99%): [α]_*D*_^25^ = +4.0 (*c* 1.0, CHCl_3_); other physical and spectroscopic properties are identical to those of **2**. Chiral HPLC: retention time 22.7 min, ee 98%.

#### (*Z*)-7-Methoxy-1-(5-(triethylsilyl)pent-4-en-1-yl)-1,2,3,4-tetrahydroquinoline (**36**)

Grubbs’ I catalyst (17.5 mg, 0.02 mmol, 0.025 equiv) was added to a solution of 7-methoxy-1-(pent-4-yn-1-yl)-1,2,3,4-tetrahydroquinoline **34** (200 mg, 0.82 mmol, 1.0 equiv) and triethylsilane (0.167 mL, 1.04 mmol, 1.2 equiv) in toluene (4 mL), and the reaction mixture was stirred at 40 °C for 2 h. After this time, TLC analysis indicated that the reaction was complete. The reaction mixture was concentrated *in vacuo*. The residue was purified using flash column chromatography (0 to 30% EtOAc in petroleum ether) to give the title compound (**36**) (167 mg, 69%) as a colorless oil: R_f_ 0.56 (petroleum ether:EtOAc 4:1); *ν̅*_max_ (thin film)/cm^–1^ 2950 (w), 2873 (w), 1610 (s), 1574 (m), 1163 (m), 727 (m), ^1^H NMR (400 MHz, CDCl_3_): δ 6.85 (1H, d, *J* 7.3), 6.42 (1H, dt, *J* 14.3, 7.2), 6.15 (1H, s), 6.14 (1H, d, 7.3), 5.47 (1H, d, *J* 14.1), 3.78 (3H, s), 3.33–3.17 (4H, m), 2.70 (2H, t, *J* 6.2), 2.17 (2H, td, *J* 7.4, 1.4), 1.98–1.89 (2H, m), 1.74–1.63 (2H, m), 0.97 (9H, t, *J* 7.9), 0.63 (6H, q, *J* 7.9); ^13^C NMR (101 MHz, CDCl_3_): δ 159.4, 149.2, 146.1, 129.5, 126.1, 115.3, 99.8, 97.4, 55.2, 51.3, 49.5, 31.8, 27.6, 26.3, 22.6, 7.7, 4.8; LRMS *m*/*z* (ES^+^) 346.2 ([M + H]^+^, 100%); HRMS *m*/*z* (ES^+^) [found: (M + H)^+^ 346.25592, C_21_H_35_NOSi requires 346.25607].

#### (*R*,*Z*)-6-(5-(7-Methoxy-3,4-dihydroquinolin-1(2*H*)-yl)pent-1-en-1-yl)-4-methyl-1,3,4,5-tetrahydro-2*H*-benzo[b][1,4]diazepin-2-one (**25**)

Boron trichloride solution (1 M in heptane 0.71 mL, 0.71 mmol, 1.75 equiv) was added to a solution of (*Z*)-7-methoxy-1-(5-(triethylsilyl)pent-4-en-1-yl)-1,2,3,4-tetrahydroquinoline **36** (0.21 g, 0.60 mmol, 1.5 equiv) in CH_2_Cl_2_ (5 mL) at 0 °C. The resulting deep-green mixture was stirred at rt for 16 h. After this time, the mixture had turned deep-blue and was cooled to 0 °C. Additional boron trichloride solution (1 M in heptane 0.71 mL, 0.71 mmol, 1.75 equiv) was added. The resulting deep-green reaction mixture was stirred at ambient temperature for a further 2 h. Methanol and toluene (30 mL, 1:1) were added, and the solution was stirred for 5 min. The solution was concentrated *in vacuo* and placed under high vacuum for 1 h. The deep blue residue was dissolved in degassed toluene (1 mL), THF (1 mL), and ethanol (0.5 mL) and added to (*R*)-6-bromo-4-methyl-4,5-dihydro-1*H*-benzo[*b*][1,4]diazepin-2(3*H*)-one (91 mg, 1.0 equiv, 0.29 mmol), (Amphos)_2_PdCl_2_ (28 mg, 10 mol %, 0.040 mmol) and potassium carbonate (279 mg, 5 equiv, 0.88 mmol) in a Schlenk flask under a nitrogen atmosphere. Degassed water (0.5 mL) was added, and the reaction mixture was stirred at 100 °C for 30 min. The reaction mixture was cooled to room temperature, and water (5 mL) was added. The mixture was extracted with EtOAc (10 mL × 3). The combined organic layers were washed with brine (15 mL), dried over anhydrous Na_2_SO_4_, filtered, and concentrated *in vacuo*. The residue was purified using flash column chromatography (0 to 100% EtOAc in petroleum ether) to yield **25** as an opaque oil (131 mg, 80%); R_f_ 0.14 (petroleum ether:EtOAc 1:1); [α]_*D*_^25^ = −12.6 (*c* 1.0, CHCl_3_); *ν̅*_max_ (thin film)/cm^–1^ 2930 (s), 2360 (w), 1669 (s), 1508 (m), 1191 (m), 732 (s); ^1^H NMR (400 MHz, CDCl_3_): δ 8.71 (1H, s), 6.92–6.87 (2H, m), 6.85–6.80 (2H, m), 6.36 (1H, d, *J* 11.2), 6.15–6.07 (2H, m), 5.90 (1H, dt, *J* 11.2, 7.4), 4.09–4.00 (1H, m), 3.75 (3H, s), 3.65 (1H, s), 3.21–3.13 (4H, m), 2.68–2.58 (3H, m), 2.44 (1H, dd, *J* 13.6, 8.0), 2.18–2.10 (2H, m), 1.89–1.79 (2H, m), 1.72–1.63 (2H, m), 1.33 (3H, d, *J* 6.3); ^13^C NMR (101 MHz, CDCl_3_): δ 173.3, 159.2, 146.0, 136.3, 135.9, 129.5, 128.5, 128.4, 126.4, 125.5, 121.3, 120.3, 115.3, 99.5, 97.4, 55.2, 54.4, 51.0, 49.5, 41.4, 27.4, 26.2, 26.1, 24.3, 22.4; HRMS *m*/*z* (ES^+^) [found: (M + H)^+^ 406.24817. C_25_H_32_N_3_O_2_ requires 406.24890]; RP-HPLC: method B: retention time 11.35 min, purity 99.3%.

#### 1-(5-(2-Fluoro-3-nitrophenyl)pent-4-yn-1-yl)-7-methoxy-1,2,3,4-tetrahydroquinoline (**37**)

Palladium(II) acetate (11 mg, 0.048 mmol, 10 mol %), triphenylphosphine (0.033 g, 0.127 mmol), copper(I) iodide (0.0024 mg, 0.0127 mmol), and 1-bromo-2-fluoro-3-nitro-benzene (**85**) (0.28 g, 1.27 mmol, 1 equiv) were added to a Schlenk tube. A solution of 7-methoxy-1-(pent-4-yn-1-yl)-1,2,3,4-tetrahydroquinoline **34** (0.35 g, 1.52 mmol, 1.2 equiv) in triethylamine was added, and the mixture was stirred at 100 °C for 1 h. After this time, the cooled reaction mixture was diluted with EtOAc (40 mL) and washed with water (50 mL) and then with saturated aqueous NaHCO_3_ (50 mL). The organic component was concentrated *in vacuo*. The residue was purified using flash column chromatography (0 to 35% EtOAc in petroleum ether) to give the title compound (**37**) as an orange oil (204 mg, 44%): R_f_ 0.38 (petroleum ether:EtOAc 4:1); *ν̅*_max_ (thin film)/cm^–1^ 2934 (w), 2835 (w), 1610 (s), 1536 (w), 1507 (s), 1308 (m), 1127 (m), 734 (s); ^1^H NMR (400 MHz, CDCl_3_): δ 7.95 (1H, ddd, *J* 8.5, 6.9, 1.8), 7.71 (1H, ddd, *J* 7.8, 6.0, 1.8), 7.23 (1H, td, *J* 8.0, 1.2), 6.87 (1H, d, *J* 8.2), 6.24 (1H, d, *J* 2.4), 6.16 (1H, dd, *J* 8.1, 2.4), 3.76 (3H, s), 3.44 (2H, t, *J* 7.2), 3.38–3.28 (2H, m), 2.72 (2H, t, *J* 6.3), 2.57 (2H, t, *J* 6.8), 2.05–1.92 (4H, m).^19^F NMR (376 MHz, CDCl_3_): δ −115.8 (s); ^13^C NMR (101 MHz, CDCl_3_): δ 159.3, 157.2, 154.6, 146.0, 138.7 (d, *J* 2.3), 129.6, 124.9 (d, *J* 3.0), 123.9 (d, *J* 5.3), 115.9 (d, *J* 15.7), 115.4, 99.7, 98.4 (d, *J* 4.0), 97.6, 72.6, 55.2, 50.3, 49.8, 27.4, 25.1, 22.5, 17.3; LRMS *m*/*z* (ES^+^) 369.2 ([M + H]^+^, 100%); HRMS *m*/*z* (ES^+^) [found: (M + H)^+^ 369.16090. C_21_H_22_N_2_O_3_F requires 369.16078]; RP-HPLC: method B: retention time 11.68 min, purity 98.4%.

#### *tert*-Butyl (*R*)-3-((2-(5-(7-methoxy-3,4-dihydroquinolin-1(2*H*)-yl)pent-1-yn-1-yl)-6-nitrophenyl)amino)butanoate (**38**)

1-(5-(2-Fluoro-3-nitrophenyl)pent-4-yn-1-yl)-7-methoxy-1,2,3,4-tetrahydroquinoline **37** (790 mg, 2.17 mmol, 1.0 equiv), (*R*)-*tert*-butyl 3-aminobutanoate (449 mg, 2.84 mmol, 1.3 equiv), and DIPEA (1.87 mL, 10.8 mmol, 5.0 equiv) dissolved in DMF (10 mL) were stirred at 90 °C for 24 h. After this time, the reaction was judged to be complete by TLC analysis. After cooling to ambient temperature, the solution was diluted with Et_2_O (20 mL), water (3 × 10 mL), and brine (20 mL), dried (Na_2_SO_4_), filtered, and concentrated *in vacuo*. The residue was purified using flash chromatography (0 to 35% EtOAc in petroleum ether) to yield **38** as an orange oil (0.76, 66%): R_f_ 0.27 (petroleum ether:EtOAc 4:1); [α]_*D*_^25^ = −97.1 (*c* 1.0, MeOH); *ν̅*_max_ (thin film)/cm^–1^ 3343 (w), 2932 (w), 1724 (m), 1506 (s), 1192 (s), 741 (m); ^1^H NMR (400 MHz, CDCl_3_): δ 8.05 (1H, dd, *J* 8.5, 1.7), 7.82 (1H, d, *J* 9.3), 7.56 (1H, dd, *J* 7.4, 1.7), 6.85 (1 H, dd, *J* 8.0, 1.1), 6.66 (1H, dd, *J* 8.5, 7.4), 6.20 (1H, d, *J* 2.4), 6.14 (1H, dd, *J* 8.1, 2.4), 5.10 (1H, dq, *J* 9.3, 6.4), 3.73 (3H, s), 3.39 (2H, t, *J* 7.2), 3.32–3.27 (2H, m), 2.69 (2H, t, *J* 6.3), 2.61–2.49 (3H, m), 2.44 (1H, dd, *J* 15.2, 6.7), 1.93 (4H, ddt, *J* 11.3, 6.1, 3.6), 1.39 (9H, s), 1.34 (3H, d, *J* 6.5); ^13^C NMR (101 MHz, CDCl_3_): δ 170.4, 159.3, 146.1, 145.8, 142.1, 136.4, 129.7, 126.8, 116.8, 115.4, 113.8, 99.8, 97.7, 95.8, 81.0, 79.0, 55.3, 50.7, 49.8, 48.2, 43.9, 28.1, 27.5, 25.4, 22.6, 22.1, 17.6; LRMS *m*/*z* (ES^+^) 508.2 ([M + H]^+^, 100%); HRMS *m*/*z* (ES^+^) [found: (M + H)^+^ 508.28027. C_29_H_38_N_3_O_5_ requires 508.28060].

#### (*R*)-6-(5-(7-Methoxy-3,4-dihydroquinolin-1(2*H*)-yl)pent-1-yn-1-yl)-4-methyl-1,3,4,5-tetrahydro-2*H*-benzo[b][1,4]diazepin-2-one (**26**)

To a solution of *tert*-butyl (*R*)-3-((2-(5-(7-methoxy-3,4-dihydroquinolin-1(2*H*)-yl)pent-1-yn-1-yl)-6-nitrophenyl)amino)butanoate **38** (350 mg, 0.68 mmol 1.0 equiv) in CH_2_Cl_2_ (8 mL) was added trifluoroacetic acid (2 mL), and the orange solution was stirred at rt for 16 h. The reaction mixture was evaporated *in vacuo*, and the resulting residue was dissolved in glacial acetic acid (5 mL). Iron powder (200 mg, 3.58 mmol, 5.0 equiv) was added, and the brown suspension was heated under reflux for 30 min. After this time, 1 M aq K_2_CO_3_ (30 mL) was added to the cooled suspension, and the resulting mixture was extracted with EtOAc (3 × 20 mL). The combined organic components were washed with 0.5 M aq LiCl (3 × 20 mL) and brine (50 mL), dried (MgSO_4_), filtered, and evaporated *in vacuo*. The residue was purified using flash chromatography (0 to 50% EtOAc in petroleum ether) to yield **26** as a colorless oil (177 mg, 44%): R_f_ 0.40 (petroleum ether:EtOAc 1:1); [α]_*D*_^25^ = −3.37 (*c* 1.0, MeOH); *ν̅*_max_ (thin film)/cm^–1^ 2937 (w), 2360 (w), 1670 (m), 1611 (s), 1163 (s), 825 (m); ^1^H NMR (400 MHz, CDCl_3_): δ 8.45 (1H, s), 7.15 (1H, dd, *J* 7.6, 1.5), 6.88–6.82 (2H, m), 6.73 (1H, dd, *J* 7.8, 7.8), 6.20 (1H, d, *J* 2.4), 6.15 (1H, dd, *J* 8.1, 2.4), 4.55 (1H, s), 4.11–4.02 (1H, m), 3.73 (3H, s), 3.41 (2H, t, *J* 7.2), 3.32–3.25 (2H, m), 2.70 (2H, t, *J* 6.3), 2.66–2.61 (1H, m), 2.57–2.47 (3H, m), 1.97–1.87 (4H, m), 1.36 (3H, d, *J* 6.4); ^13^C NMR (101 MHz, CDCl_3_): δ 173.0, 159.3, 146.1, 139.5, 129.7, 128.9, 126.3, 122.1, 119.5, 115.4, 113.9, 99.9, 97.6, 95.5, 77.7, 55.2, 53.2, 50.6, 49.9, 42.1, 27.5, 25.8, 24.2, 22.6, 17.4; HRMS *m*/*z* (ES^+^) [found: (M + H)^+^ 404.23196. C_25_H_30_N_3_O_2_ requires 404.23325]; RP-HPLC: method B: retention time 11.42 min, purity 98.9%.

#### (*R*)-6-(5-(7-Methoxy-3,4-dihydroquinolin-1(2*H*)-yl)pentyl)-4-methyl-1,3,4,5-tetrahydro-2*H*-benzo[b][1,4]diazepin-2-one (**27**)

To a solution of (*R*)-6-(5-(7-methoxy-3,4-dihydroquinolin-1(*2H*)-yl)pent-1-yn-1-yl)-4-methyl-1,3,4,5-tetrahydro-*2H*-benzo[*b*][1,4]diazepin-2-one **26** (45 mg, 0.11 mmol, 1.0 equiv) in EtOH (3 mL) was added 10% Pd/C (10 mg, 0.011 mmol, 0.1 equiv). The system was purged with nitrogen, and then hydrogen gas was added. The suspension was stirred for 3 h under an atmosphere of hydrogen at ambient temperature. The black suspension was filtered through Celite, dried (MgSO_4_), filtered, and concentrated *in vacuo*. The residue was purified using flash column chromatography (0 to 60% EtOAc in petroleum ether) to yield **27** as a colorless oil (35 mg, 78%): R_f_ 0.20 (petroleum ether:EtOAc 1:1); [α]_*D*_^25^ = −12.7 (*c* 1.0, MeOH); *ν̅*_max_ (thin film)/cm^–1^ 2932 (w), 2360 (m), 1670 (s), 1199 (s), 748 (m); ^1^H NMR (400 MHz, CDCl_3_): δ 8.18 (1H, s), 6.95 (1H, dd, *J* 7.2, 2.0), 6.89–6.80 (3H, m), 6.16–6.09 (2H, m), 4.12–4.00 (1H, m), 3.76 (3H, s), 3.55–3.35 (1H, m), 3.29–3.19 (4H, m), 2.68 (2H, t, *J* 6.4), 2.64–2.51 (3H, m), 2.37 (1H, dd, *J* 13.4, 7.1), 1.96–1.84 (2H, m), 1.71–1.56 (4H, m), 1.47–1.38 (2H, m), 1.36 (3H, d, *J* 6.2); ^13^C NMR (101 MHz, CDCl_3_): δ 173.1, 159.2, 146.1, 136.8, 132.9, 130.0, 129.5, 126.4, 121.4, 120.3, 115.2, 99.2, 97.5, 55.3, 55.2, 51.4, 49.5, 40.8, 31.7, 30.1, 27.4, 27.2, 26.1, 24.0, 22.4; HRMS *m*/*z* (ES^+^) [found: (M + H)^+^ 408.26297. C_25_H_34_N_3_O_2_ requires 408.260455]; RP-HPLC: method B: retention time 11.57 min, purity 100.0%.

## Supporting Information Available

Supplementary figures and schemes, additional biological experimental details, NMR and HPLC traces of novel compounds (PDF)
Molecular formula strings for biologically tested compounds (CSV)
PDB files for docking sessions (ZIP)

## Abbreviations

BRD4: bromodomain-containing protein 4
BRD4(1): the first bromodomain of BRD4
BRD4(2): the second bromodomain of BRD4
CBP: cAMP response element binding protein binding protein
CREBBP: cAMP response element binding protein binding protein
E2F: transcription factor
GATA1: erythroid transcription factor
HAT: histone acetyl transferase
HIF: hypoxia inducible factor
IBiD: interferon binding domain
KAT: lysine acetyl transferase
KIX: kinase inducible
NRID: N-terminal nuclear receptor interaction domain
p53: tumor suppressor p53
p73: tumor suppressor p73
PHD: plant homeodomain
PrOF: protein observed fluorine (NMR)
RING: really interesting new gene
RTS: Rubinstein-Taybi syndrome
TAZ: transcription adaptor zinc finger
ZZ: zinc finger domain that binds two zinc ions
