# Supplementary material for: Controlling Intramolecular Interactions in the Design of Selective, High-Affinity Ligands for the CREBBP Bromodomain
Source: J Med Chem. 2021 Jul 13;64(14):10102–23. doi: 10.1021/acs.jmedchem.1c00348 (PMC8311651; doi:10.1021/acs.jmedchem.1c00348)
Supplement: Supplementary file 1 — jm1c00348_si_001.pdf [file jm1c00348_si_001.pdf]

Supporting Information: Controlling Intramolecular Interactions in the Design of Selective, High-Affinity, Ligands for the CREBBP Bromodomain

Michael Brand,[†] *James Clayton,*[†] *Mustafa Moroglu,*[†] *Matthias Schiedel,*[†] *Sarah Picaud,*[‡] *Joseph P. Bluck,*[†,§] *Anna Skwarska,*[‖] *Hannah Bolland,*[‖] *Anthony K. N. Chan,*[†] *Corentine M. C. Laurin,*[†] *Amy R. Scorah,*[†] *Larissa See,*[†] *Timothy P. C. Rooney,*[†] *Katrina H. Andrews,*[†] *Oleg Fedorov,*[‡] *Gabriella Perell,*[#] *Prakriti Kalra,*[#] *Kayla B. Vinh,*[#] *Wilian A. Cortopassi,*[†] *Pascal Heitel,*[†] *Kirsten E. Christensen,*[†] *Richard I. Cooper,*[†] *Robert S. Paton,*[†,▿] *William C. K. Pomerantz,*[#] *Philip C. Biggin,*[§] *Ester M. Hammond,*[‖] *Panagis Filippakopoulos,*[‡] *Stuart J. Conway*[†,*]

[†]Department of Chemistry, Chemistry Research Laboratory, University of Oxford, Mansfield Road, Oxford OX1 3TA, U.K.

[‡]Nuffield Department of Clinical Medicine, Structural Genomics Consortium, University of Oxford, Old Road Campus Research Building, Roosevelt Drive, Oxford OX3 3TA, United Kingdom.

[§]Department of Biochemistry, University of Oxford, South Parks Road, Oxford OX1 3QU, U.K.

[‖]Oxford Institute for Radiation Oncology, Department of Oncology, University of Oxford, Oxford, OX3 7DQ, United Kingdom.

[#]Department of Chemistry, University of Minnesota, 207 Pleasant Street SE, Minneapolis, Minnesota 55455, U.S.A.

[▿]Department of Chemistry, Colorado State University, 1301 Center Ave, Ft. Collins, CO 80523-1872, U.S.A.

*To whom correspondence should be addressed: stuart.conway@chem.ox.ac.uk

Table of Contents

**Content**	**Pages**
Supplementary Figures	S3-S17
Additional description of Schemes 1 & 2	S18-S19
Experimental section	S20-S31
Tables S1 & S2	S32-S34
NMR spectra of novel compounds	S35-S72
HPLC traces of biologically tested compounds	S73-S81
References	S82-S84

Figure S1. Structures of representative CREBBP bromodomain ligands **1**,[1] SGC-CBP30,[2] I-CBP112,[3] compound **19**,[4] XDM-CBP,[5] CPI-637,[6] GNE-272,[7] GNE-781,[8] and compound **32h**.[9]

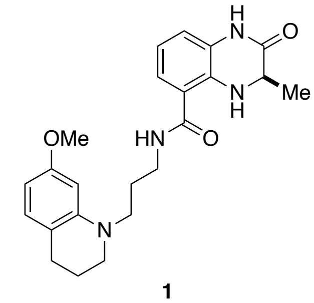

1
CREBBP K_d = 390 nM (ITC)

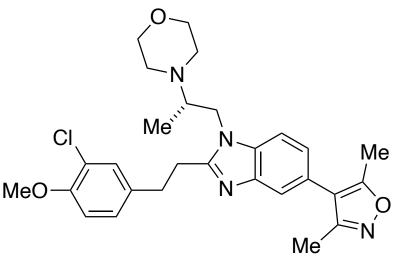

SGC-CBP30
CREBBP K_d = 21 nM (ITC)

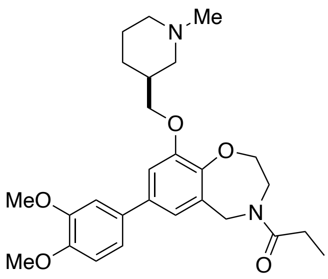

I-CBP112
CREBBP K_d = 151 nM (ITC)

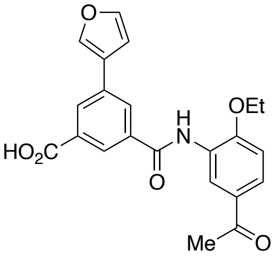

Compound 19
CREBBP $K_d \approx$ 300 nM (ITC)

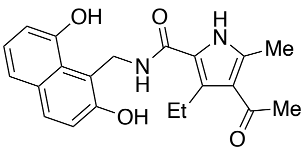

XDM-CBP
CREBBP K_d = 230 nM (ITC)

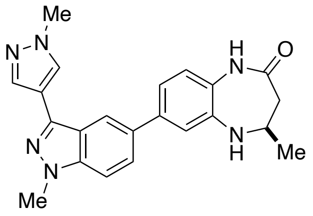

CPI-637
CREBBP $IC_{50} \approx$ 30 nM (TR-FRET)

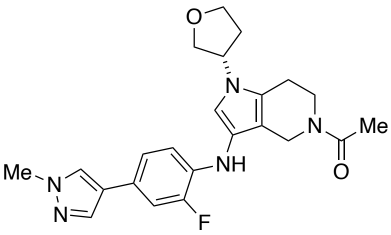

GNE-272
CREBBP $IC_{50} \approx$ 20 nM (TR-FRET)

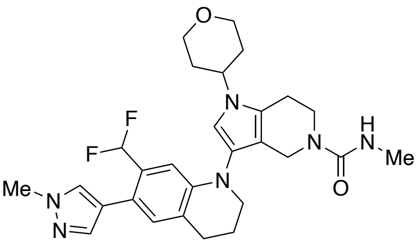

GNE-781
CREBBP IC_{50} = 0.94 nM (TR-FRET)

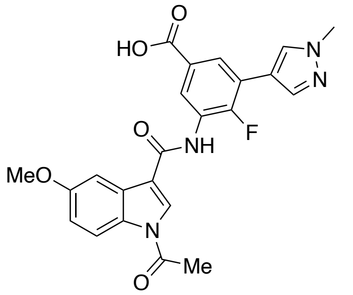

Compound 32h
CREBBP IC_{50} = 37 nM (AlphaScreen)

Figure S2. Stability monitoring of (**A**) the 3,4-dihydroquinoxalinone **1** and (**B**) the 4,5-dihydrobenzodiazepinone **6** using ^{1}H NMR.

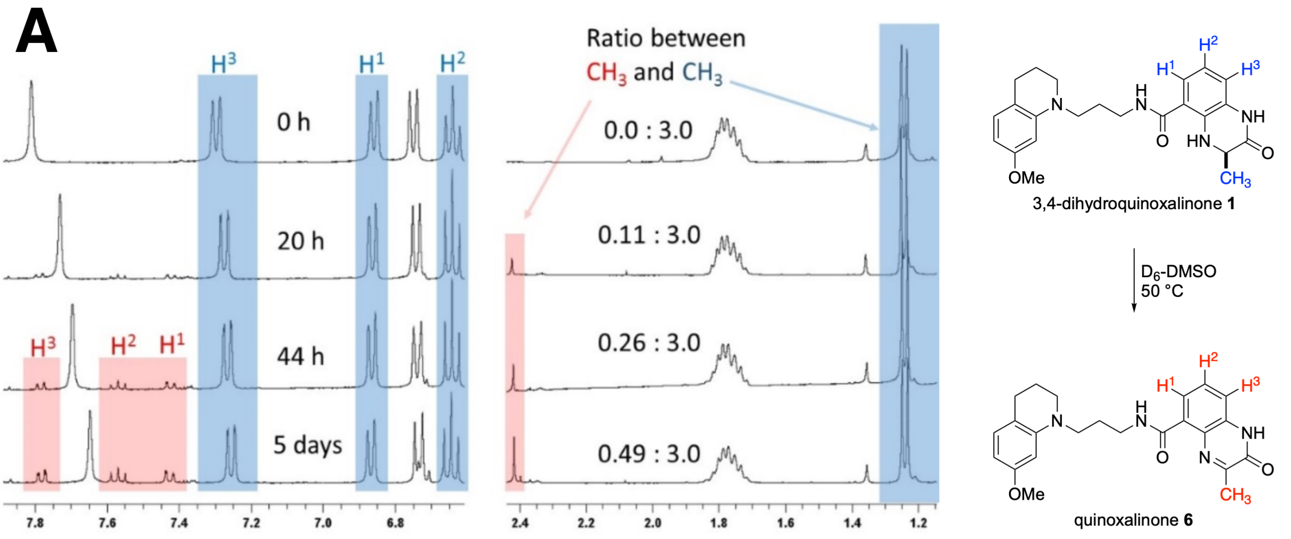

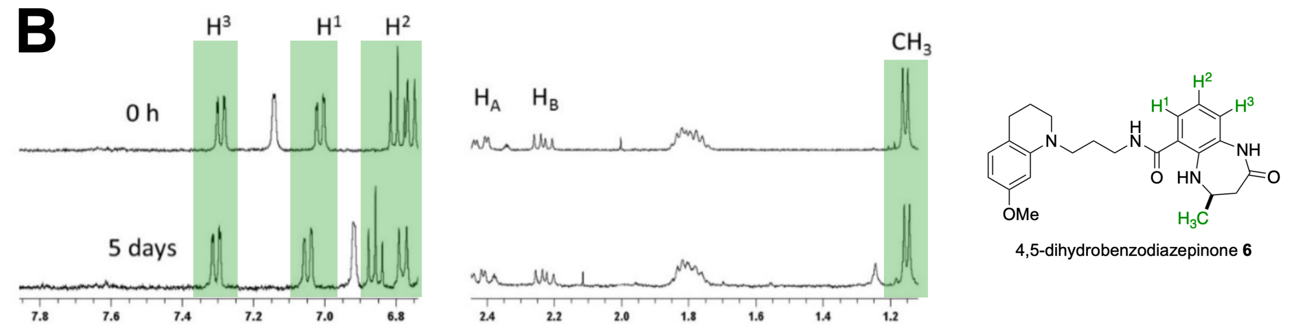

Figure S3. A snapshot of the MD simulation of **6** (carbon = purple) bound to the CREBBP bromodomain overlaid with the X-ray crystal structure of **1** (carbon = yellow) bound to the CREBBP bromodomain (PDB code 4NYX).[1] **A**. The predicted interactions of **6** (carbon = purple) with N1168 are shown. **B**. The predicted hydrophobic interactions with V1115 and I1122 can be seen. **C**. Root mean square deviation (RMSD) of the Cα atoms from the initial structure over 120 ns of the MD simulation for **6**.

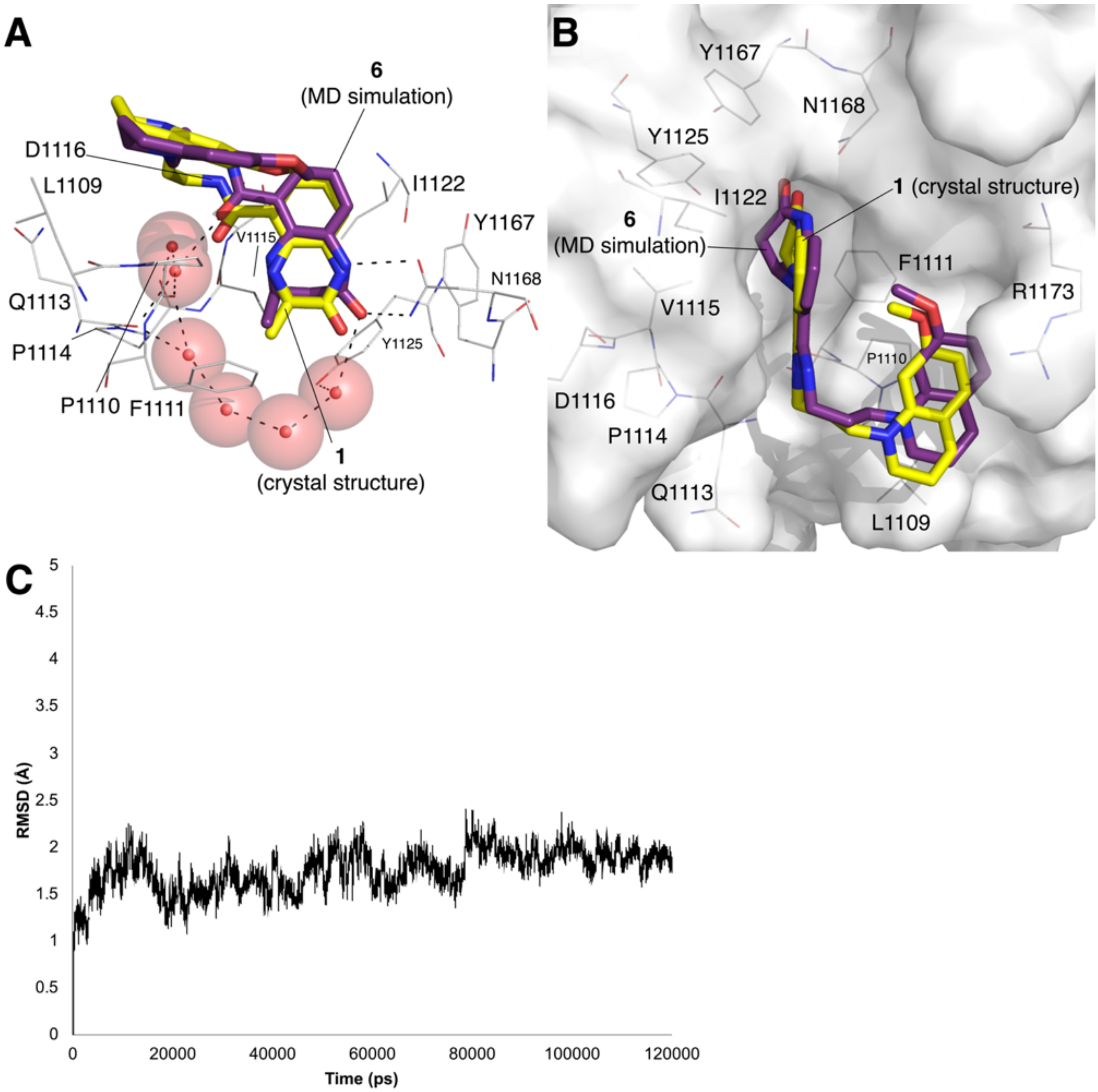

We initially validated this computational approach by replicating the binding pose observed in the X-ray crystal structure of **1** bound to the CREBBP bromodomain (PDB code 4NYX).[1] We then replicated this procedure for the 4,5-dihydrobenzodiazepinone **65** using the *apo*-structure of the CREBBP bromodomain (PDB code 3P1C). The resulting predicted structure of **65** matched well with the X-ray crystal structure of **1** bound to the CREBBP bromodomain.

Figure S4. Partial ^{19}F NMR spectra showing the effect of adding 17.5, 35 or 70 μM of either (**A**) I-CPB112 or (**B**) bromosporine to the 5-fluorotryptophan-labeled CREBBP bromodomain (35 μM). **C.** 1D-^{19}F NMR spectra of 5FW-labeled wild type and W to F point mutated CREBBP bromodomain. Asterisks represent the position of missing peaks corresponding to the mutated tryptophan.

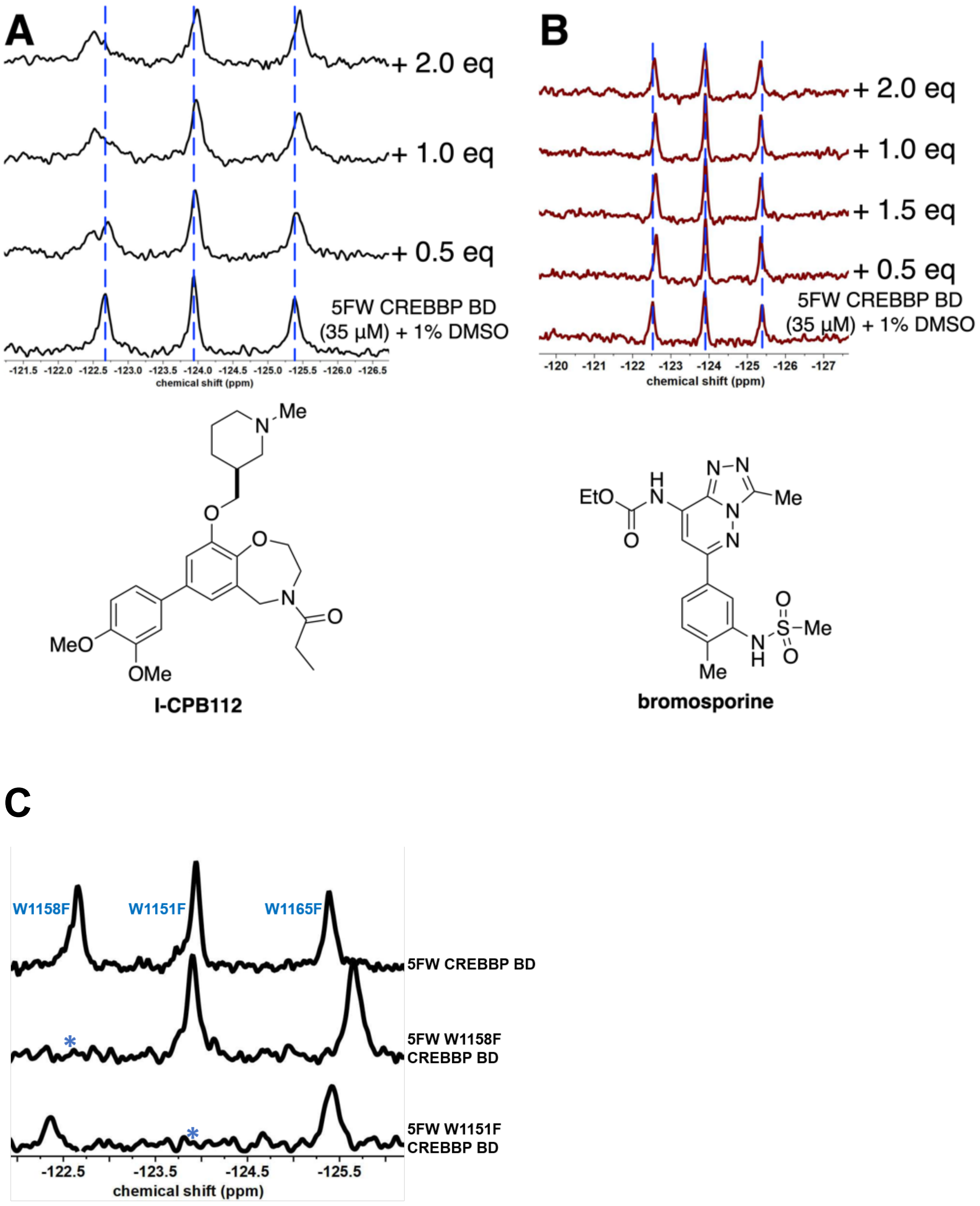

Figure S5. Study to observe the internal hydrogen bond (proton shown in magenta) in solution. ^{1}H NMR spectra of **6** dissolved in different ratios of $CDCl_3$ and D_6-DMSO. All experiments were performed at a compound concentration of 2 mg/mL.

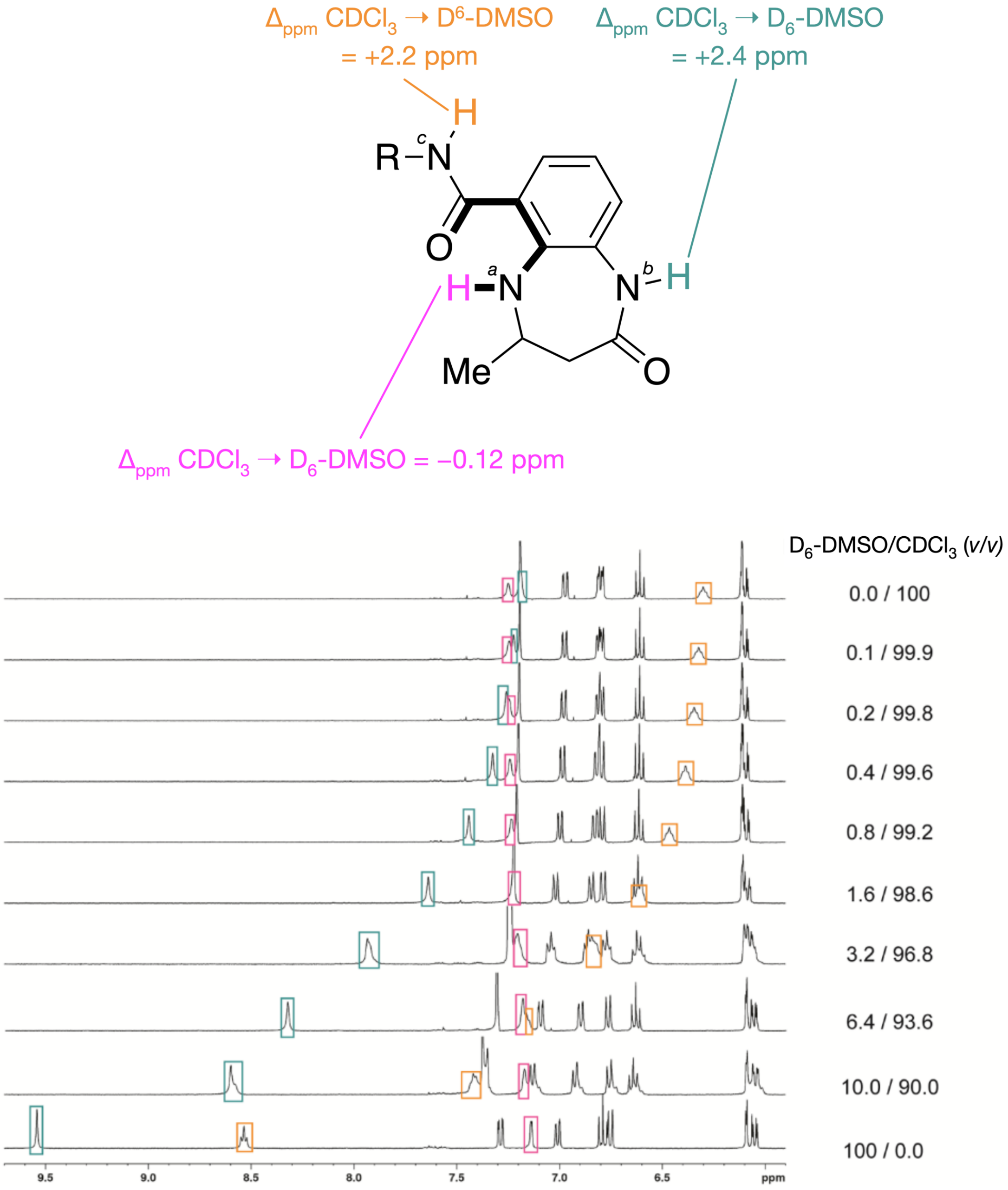

Figure S6. A. The ITC signature plot for compound **6**. **B.** The ITC signature plot for compound **2**. The ΔG value (blue) is the sum of the ΔH (green) and −TΔS (red) contributions.

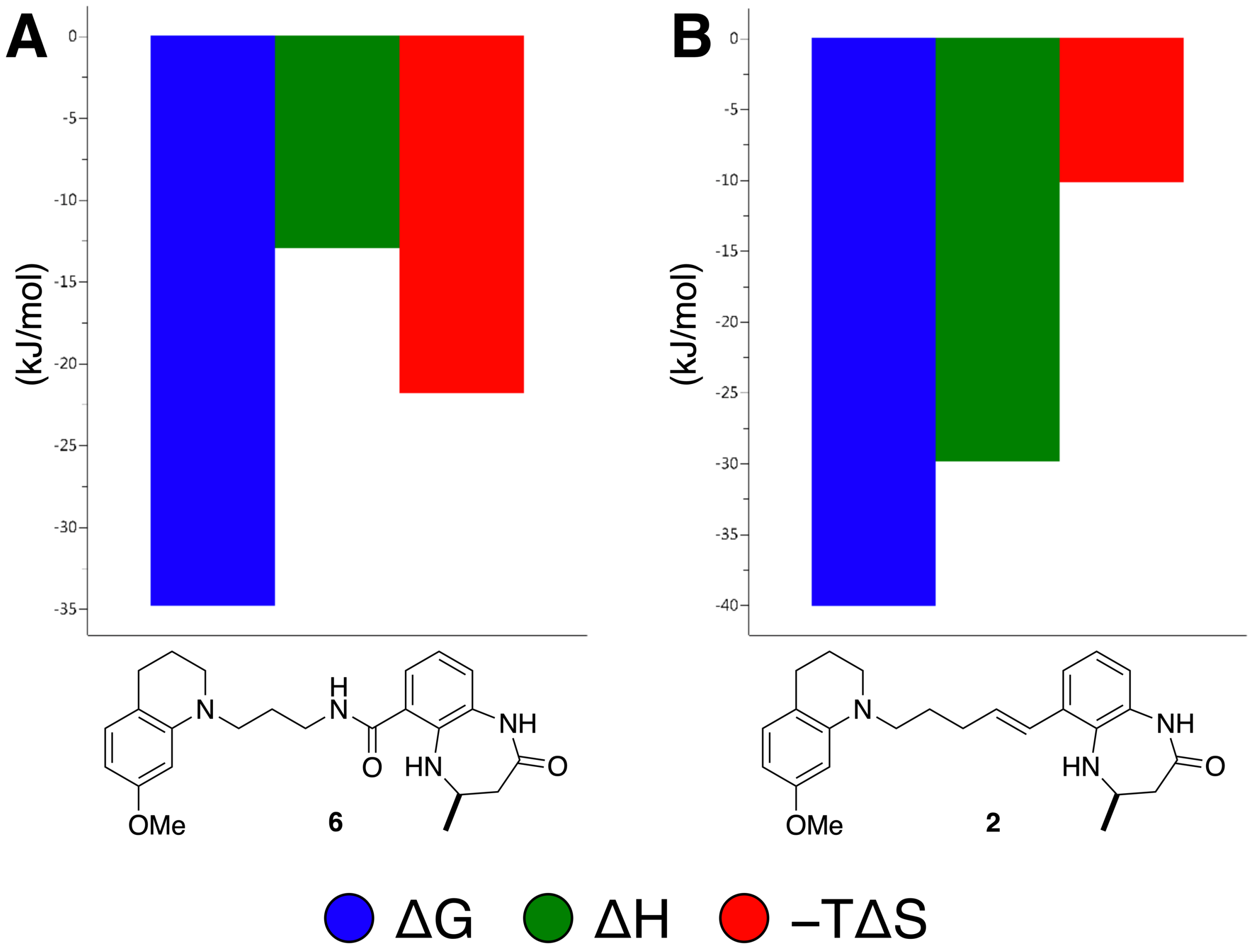

Figure S7. Ligands **6**, **2**, **25** and **26** retain their crystallographic or docked poses during equilibrium MD. The crystallographic structure of **6** (**A**) and docked structures of **2** (**B**), **25** (**C**) and **26** (**D**) were subjected to MD (50 ns) in triplicate. The respective ligand RMSD plots (**E-H**) show that the ligands retain these poses for the majority of the simulations.

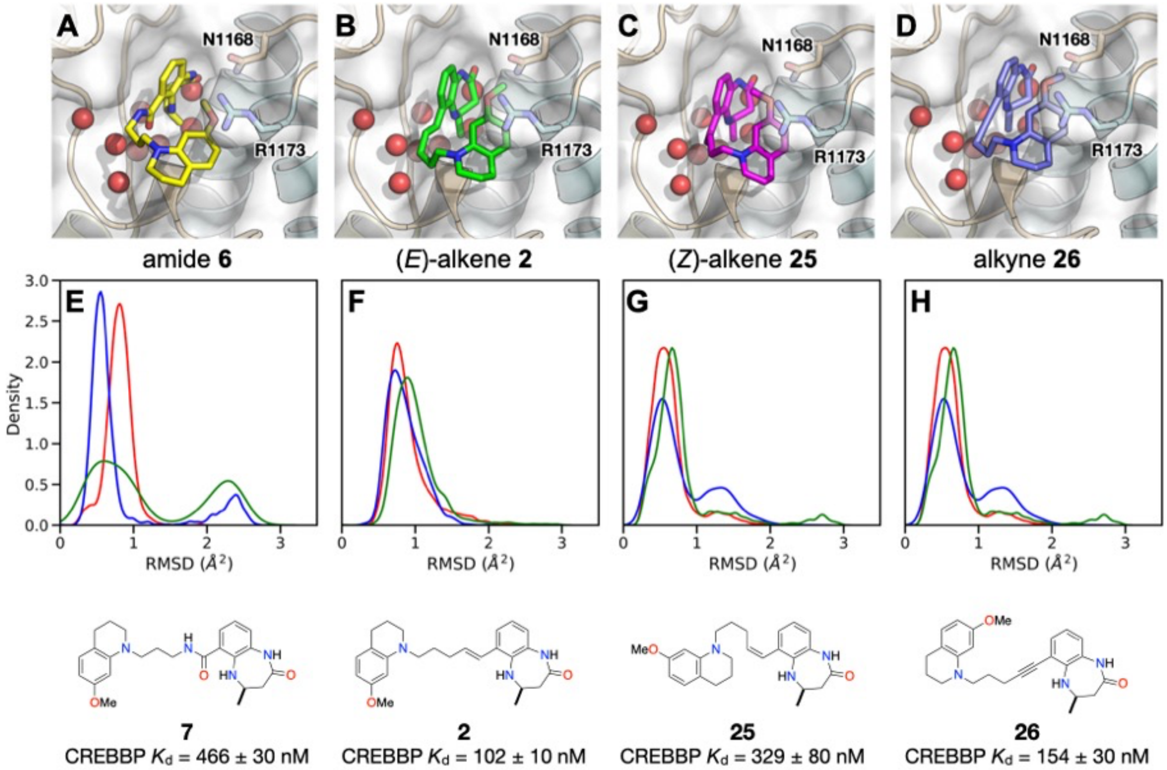

Figure S8. Analysis compounds **2**, **6**, or **25** binding to BRD4(1) using ligand-observed ^{1}H NMR. The reduction of the signal for the 4,5-dihydrobenzodiazepinone methyl group upon BRD4(1) addition was used to monitor BRD4(1) binding. **A.** Overlay of spectra of the free ligand **6** (blue), and the ligand in the presence of BRD4(1) at 1 µM (red), 2 µM (green), 3 µM (purple), 5 µM (yellow) and 10 µM (orange). **B**. Overlay of spectra of the free ligand **2** (blue), and the ligand in the presence of BRD4(1) at 1 µM (red), 2 µM (green), 2.5 µM (purple), 5 µM (yellow), 10 µM (orange) and 25 µM (light green). **C**) Overlay of spectra of the free ligand **25** (blue), and the ligand in the presence of BRD4(1) at 1 µM (red), 2 µM (green) and 5 µM (yellow). **D.** Binding curves for the interaction between BRD4(1) and **6**, **2**, and **25**, respectively. The curves were derived from the integral reduction for the signal of the methyl group located at 4,5-dihydrobenzodiazepinone headgroup upon BRD4(1) addition, detected by means of Ligand-observed ^{1}H NMR. **E** K_d values were determined using the one site-specific binding model least square fit non-linear regression from Graphpad Prism®.

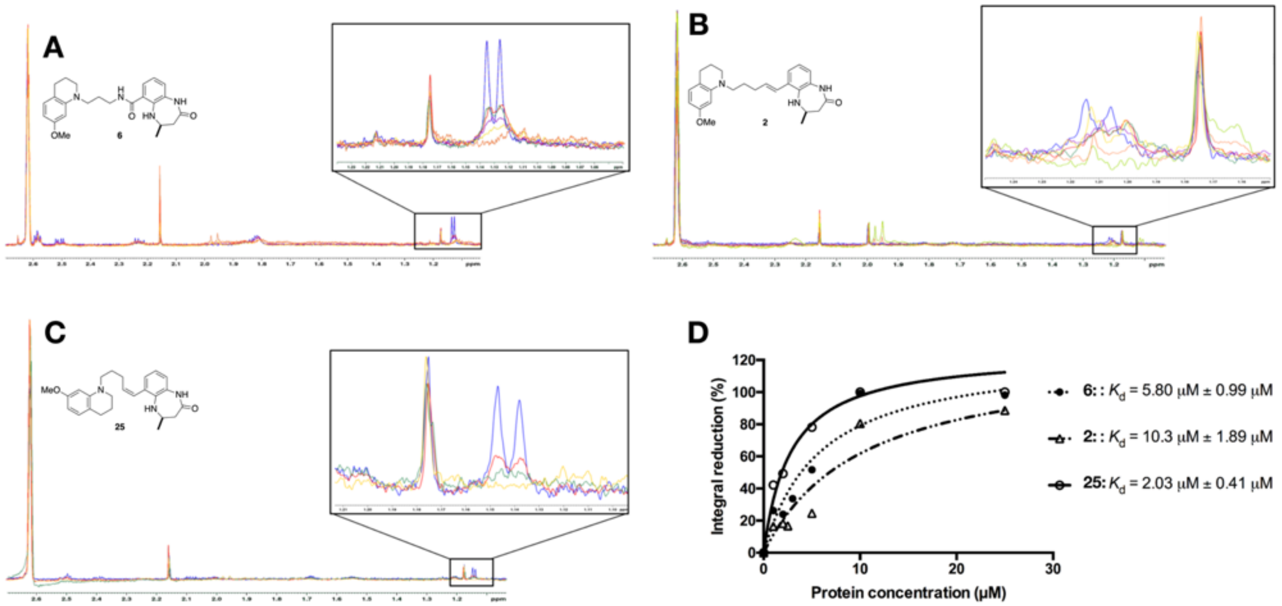

E

$$Y = \frac{B_{max} \times X}{K_d + X}$$

X = concentration of the ligand

Y = specific binding

B_{max} = maximum binding in the same units as Y

K_d = dissociation constant in the same units as X

Figure S9. Size Exclusion Chromatography (SEC) to investigate CREBBP bromodomain dimerization in solution. The similarity in the SEC traces in the presence of (**A**) DMSO and (**B**) compound **2** (50 μM) is consistent with no CREBBP bromodomain dimer formation in solution, in the presence of compound **2**.

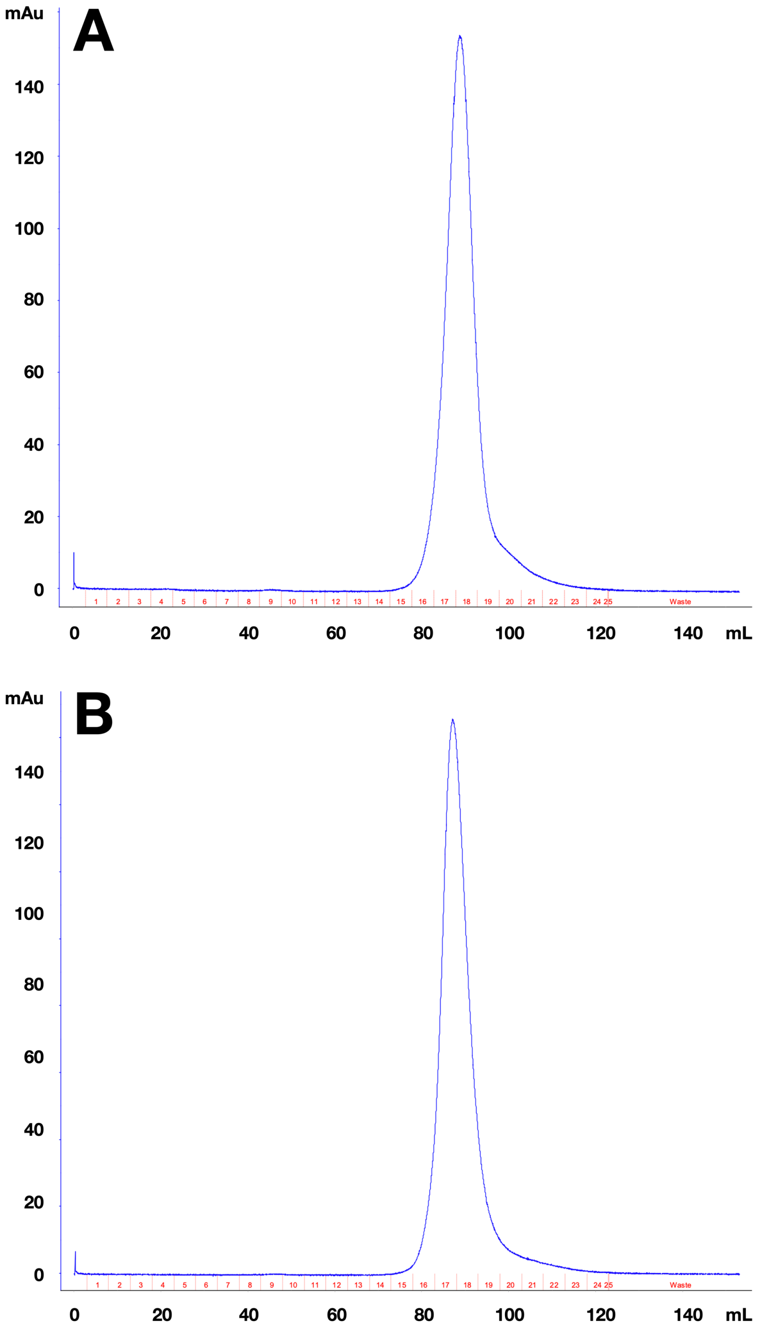

Figure S10. Stick representation of CREBBP ligands shown within the experimental electron density (FcFo map contoured at 2 sigma) for compound (**A**) **6** (PDB: 6YIM) and (**B**) **2** (PDB: 6YIJ).

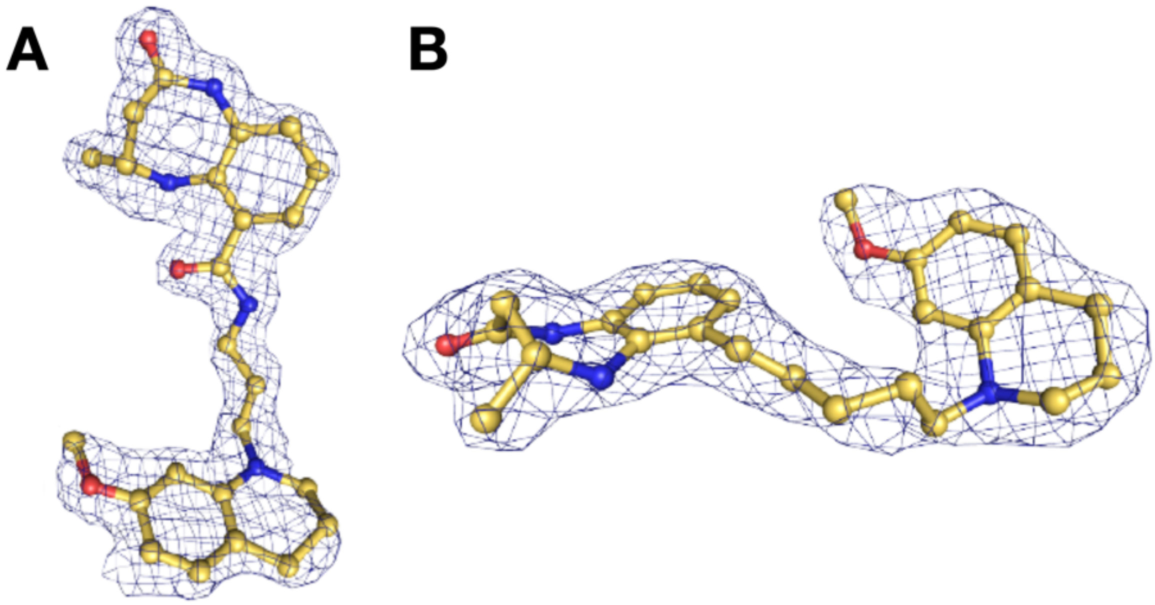

Figure S11. The isothermal titration calorimetry (ITC) data for the compounds shown, measured for the CREBBP bromodomain. **CPI-637**[6] was used as a positive control. Shown are heat effects for each injection (above) and the normalized binding isotherms (below) including the fitted function for compounds that showed binding (solid line).

6

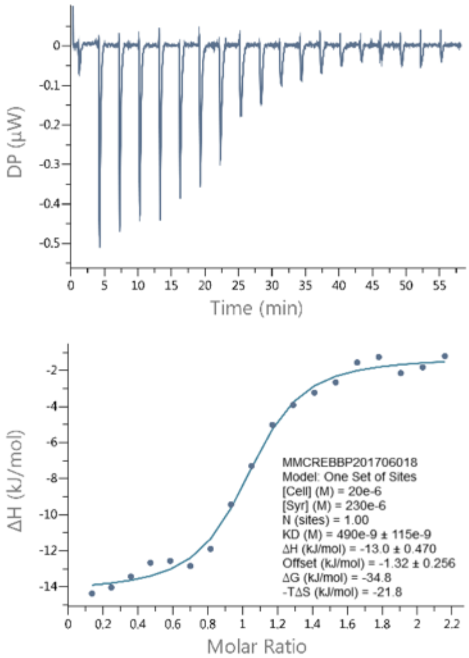

7

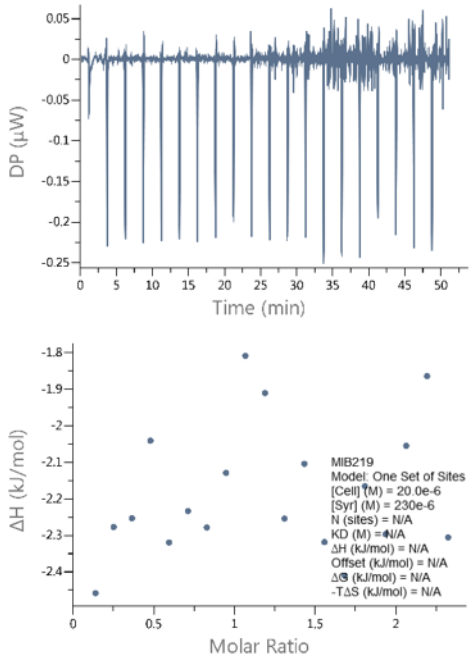

2

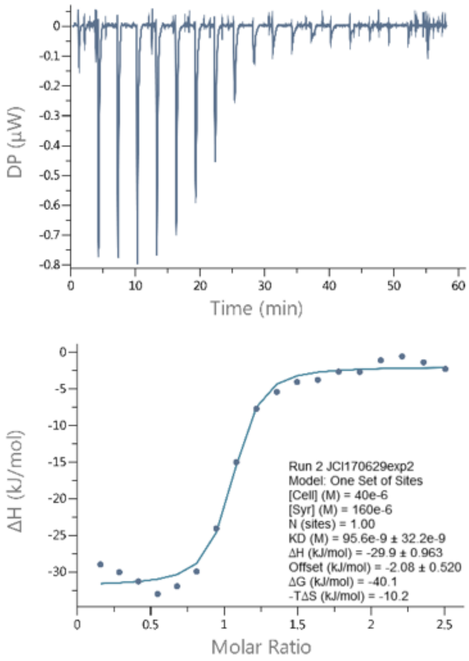

3

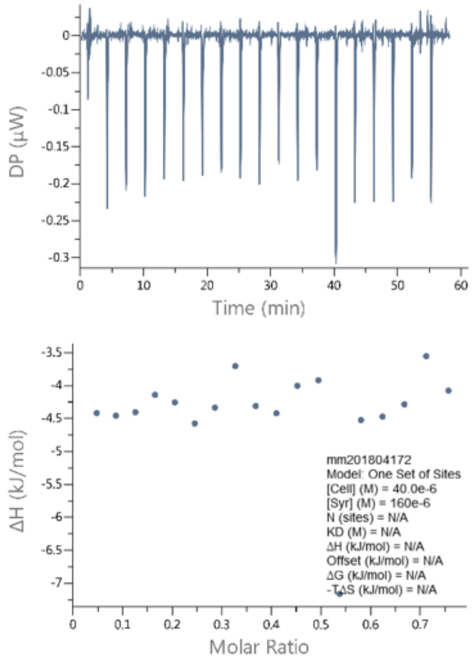

25

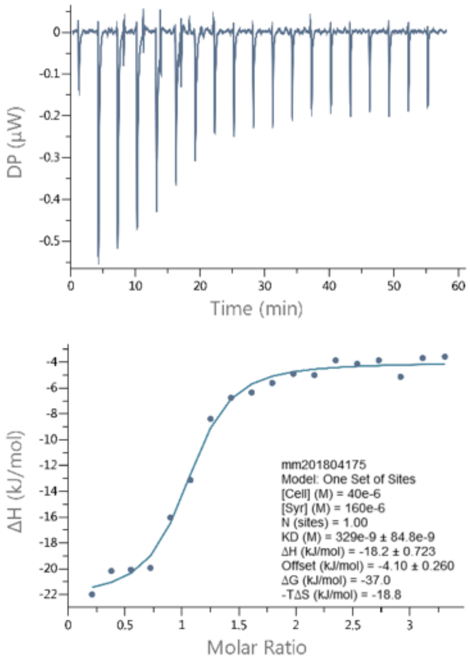

26

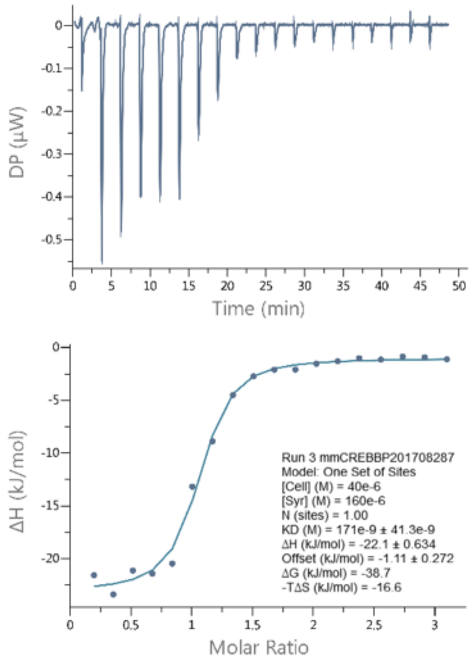

27

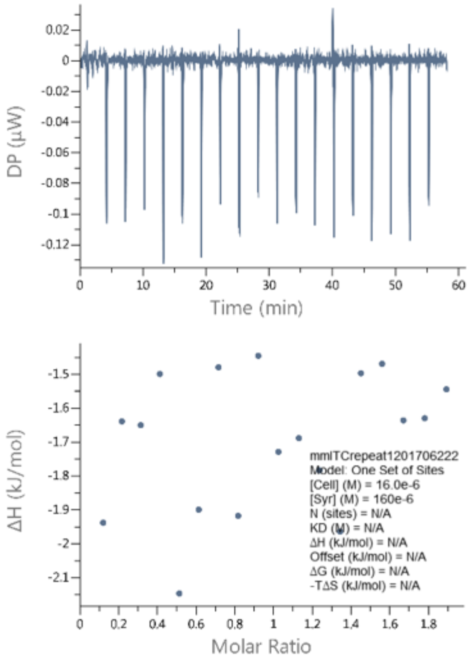

CPI-637

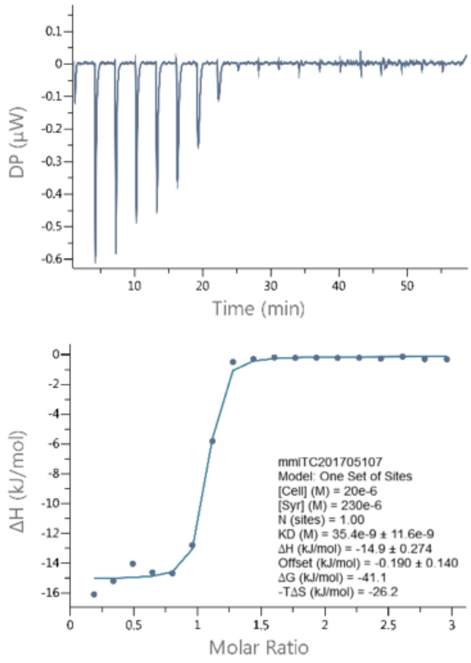

Figure S12. Isothermal titration calorimetry (ITC) data for the compounds shown, measured for BRD4(1). **OXFBD4**[10] was used as a positive control. Shown are heat effects for each injection (above) and the normalized binding isotherms (below) including the fitted function for compounds that showed binding (solid line).

6

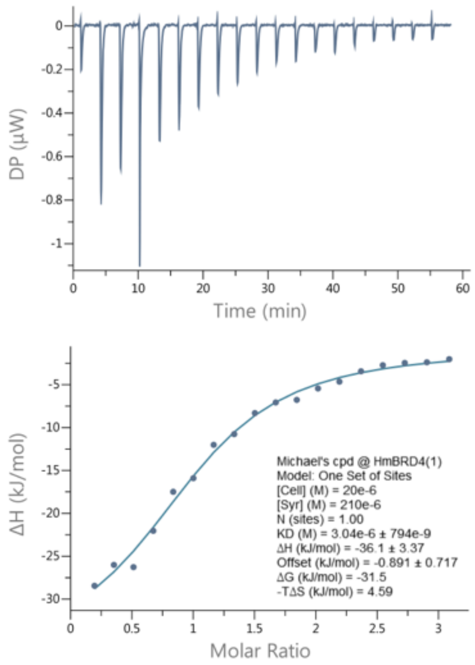

2

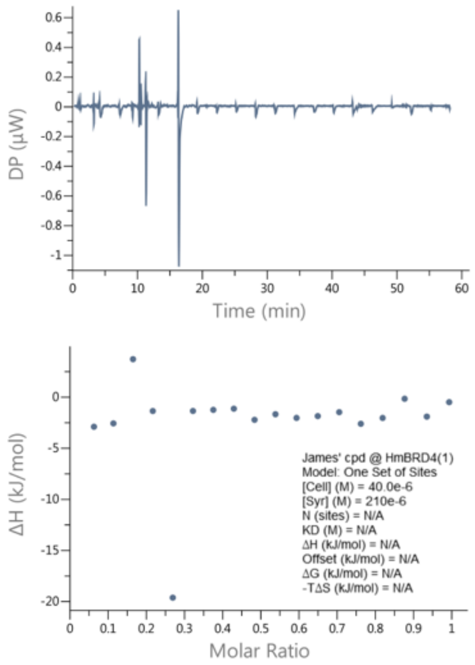

25

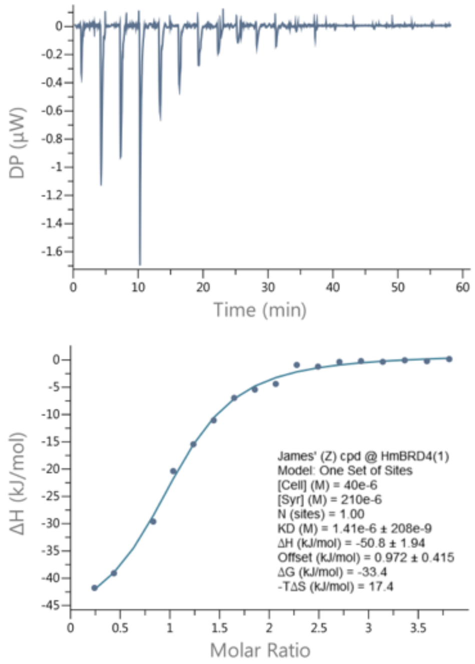

26

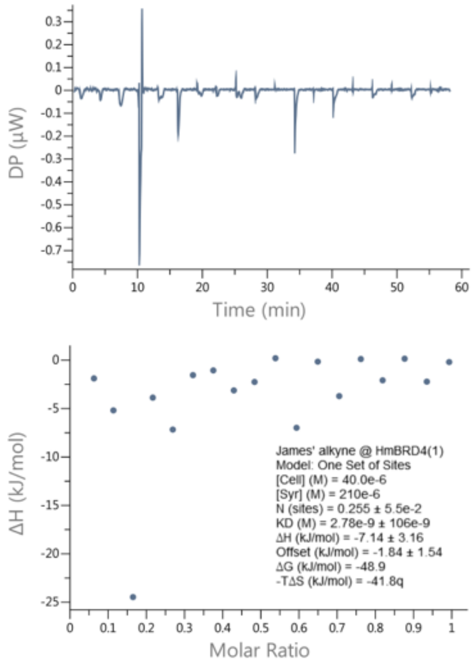

OXF-BD04

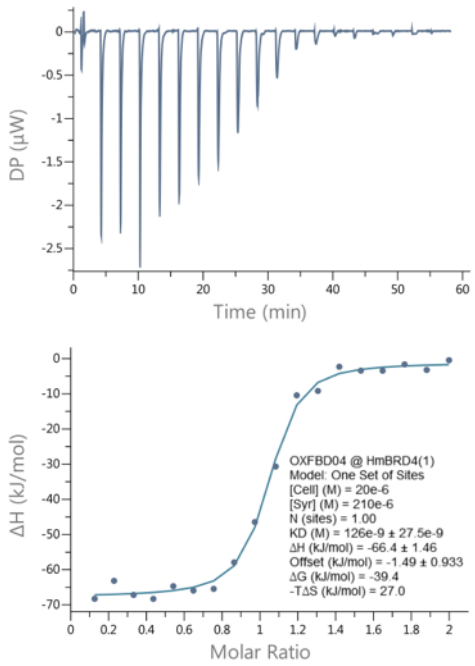

DP (µW)
Time (min)
ΔH (kJ/mol)
Molar Ratio
OXFBD04 @ HmBRD4(1)
Model: One Set of Sites
[Cell] (M) = 20e-6
[Syr] (M) = 210e-6
N (sites) = 1.00
KD (M) = 126e-9 ± 27.5e-9
ΔH (kJ/mol) = -66.4 ± 1.46
Offset (kJ/mol) = -1.49 ± 0.933
ΔG (kJ/mol) = -39.4
-TΔS (kJ/mol) = 27.0

Figure 13. X-Ray crystal structure of **6** bound to the CREBBP bromodomain (PDB code 6YIM, carbon = purple, protein surface from this structure shown) overlaid with the X-ray crystal structure of CPI-637 bound to the CREBBP bromodomain (PDB code 5I8G, carbon = orange).[6] **A**. This orientation shows that the headgroups of each compound adopt the same conformation when bound to the bromodomain, and that the KAc-mimicking methyl and carbonyl groups of both molecules overlay very closely. **B**. The top orientation shows that the interaction with R1173 is present for both molecules.

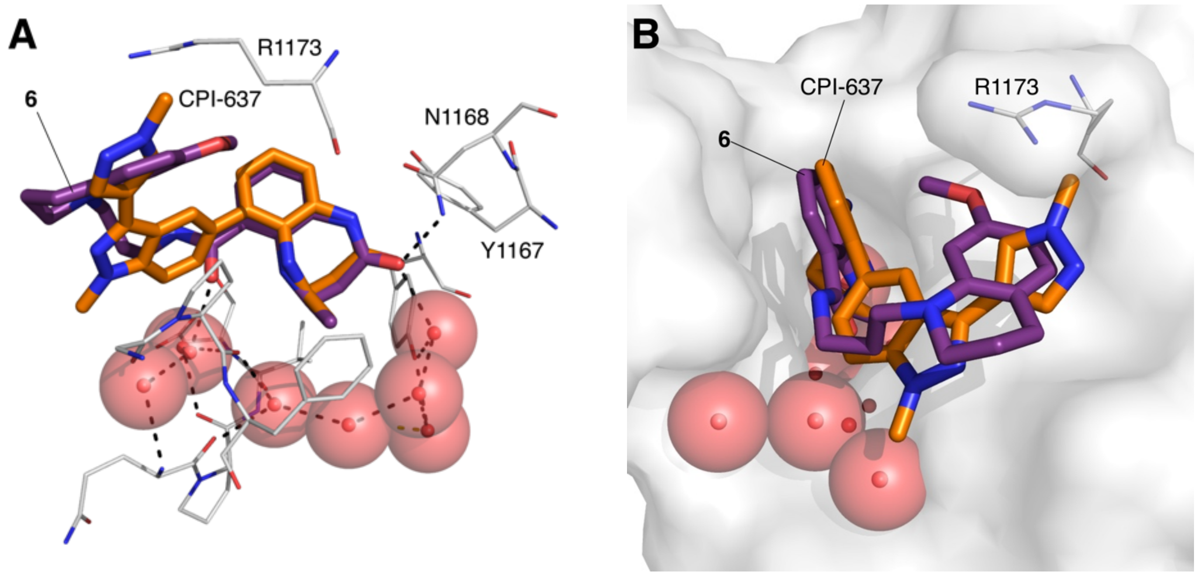

Description of the synthesis shown in Scheme 1

To make THBD analogues **6-8**, which have methyl groups positioned at two different positions, we prepared amines with the required methylation patterns, which could then be used in S_NAr reactions (Scheme 1B). For enantiomers **6** and **7**, we made the enantiopure chiral amines **12** and **13** using a chiral lithium amide reported by Davies *et al.*[11] Performing a conjugate addition to *tert*-butyl crotonate **9** gave **10** and **11**, which were hydrogenolysed with Pearlman's catalyst. Following the S_NAr reaction with **17**, the THBD headgroups were made in a two-step procedure, using iron in acetic acid to reduce the nitro group and form lactams **20** and **21** (Scheme 1C). Compound **8** was prepared as a racemic mixture. The synthesis used a conjugate addition of benzylamine to *tert*-butyl methacrylate ester **14**, followed by hydrogenolysis of the benzyl group with Pearlman's catalyst, to form a racemic mixture of **16**. This compound was reacted with **17**, followed by three-step to form lactam **23**. The THBD headgroups followed a benzyl deprotection and amide coupling with amine **24** to give the final THBD analogues.

Description of the synthesis shown in Scheme 2

A cross coupling approach was used to link the THBD and THQ fragments together, as it allowed rapid synthesis of all desired analogues. The preparation of THBD headgroups **31** and **32**, which have a bromide substituent in place of the ester, were synthesized by S_NAr reaction of **28** with the amines **12** and **13**. Treatment of the products with iron and acetic acid yielded the THBD headgroups **31** and **32**. The THQ fragments were prepared from the alkylation of THQ **33** to yield alkyne **34**. The alkyne (**34**) underwent an hydroboration with HBPin to give pinacol borate ester **35**, which was prepared with a catalytic amount of Schwartz's reagent to give the *E*-geometry in high selectivity.[12] Compound **35** was reacted in a Suzuki-Miyaura cross-coupling with **31** or **32** to give both enantiomers with *E*-double bond stereochemistry. To achieve the *Z*-double bond geometry, we employed a ruthenium-

catalyzed alkyne hydrosilylation, described by Aricó and Cox, to give **36**.[13] Compound **36** was treated with BCl_3 to form a boronate ester, which was used directly in a Suzuki-Miyaura cross-coupling reaction with **31** to give **25**, which contains the *Z*-double bond. To form the alkyne **26**, we initially tried a Heck alkynylation of compound **31** using **24**, however, this resulted in an unwanted Larock-indole side-product as the major product. To avoid this side reaction, alkyne **34** was used in a Sonogashira coupling with **28**. Compound **26** was afforded by an S_NAr reaction followed by a lactam ring closure, as described above. Compound **27** was prepared by hydrogenation of **26**.

Experimental Section

Bromodomain construct and plasmid information.

H_6-BRD4(1) (44…168aa) sequence:

MHHHHHHSSGVDLGTENLYFQSMNPPPPETSNPNKPKRQTNQLQYLLRVVLKTLWKH

QFAWPFQQPVDAVKLNLPDYYKIIKTPMDMGTIKKRLENNYYWNAQECIQDFNTMFT

NCYIYNKPGDDIVLMAEALEKLFLQKINELPTEE

Plasmid: BRD4 (Addgene #38942)

Vector backbone: pNIC28-Bsa4 (Addgene #26103)

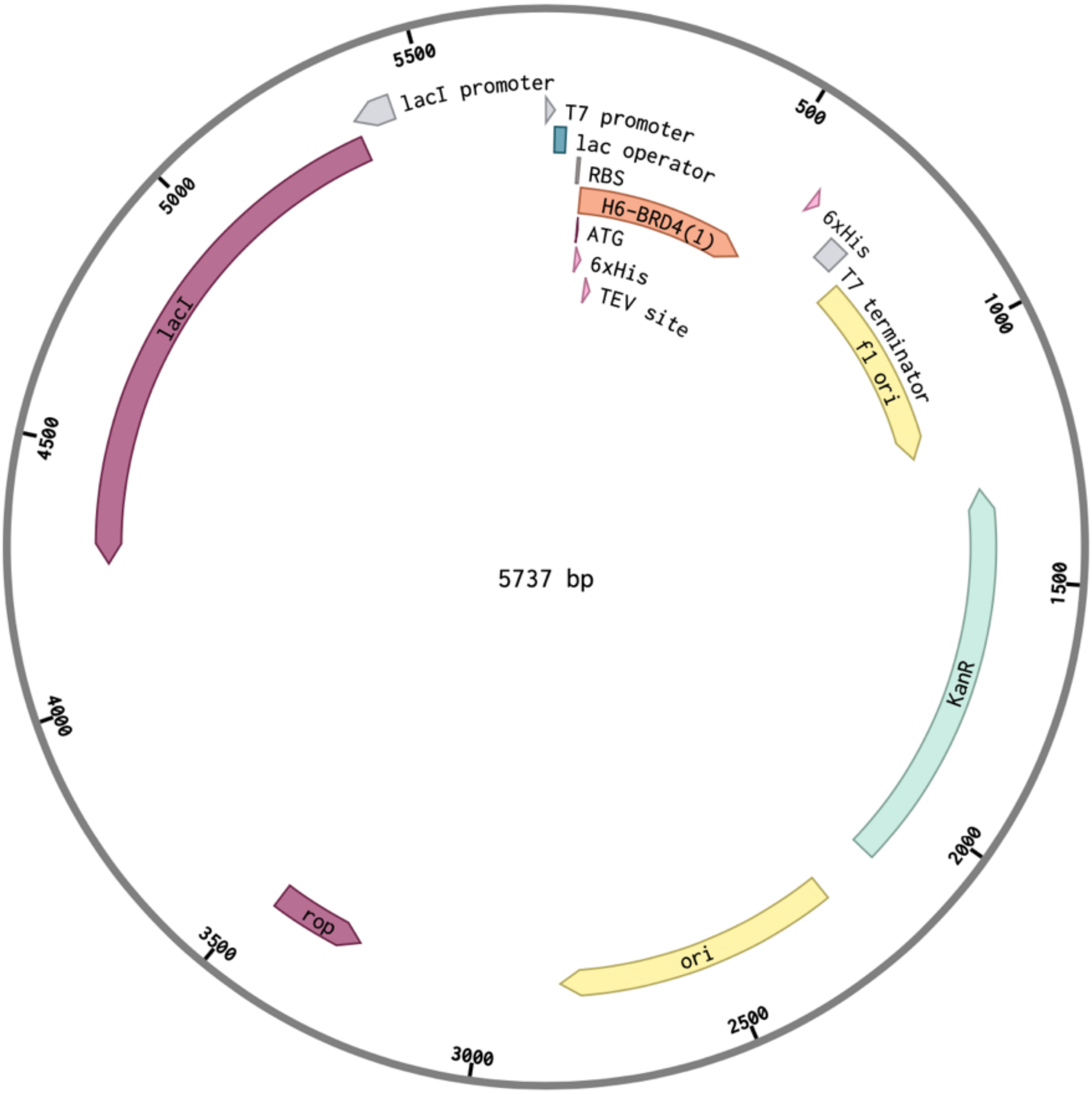

H_6-CREBBP (1081…1197aa) sequence:

MHHHHHHSSGVDLGTENLYFQSMRKKIFKPEELRQALMPTLEALYRQDPESLPFRQP

VDPQLLGIPDYFDIVKNPMDLSTIKRKLDTGQYQEPWQYVDDVWLMFNNAWLYNRKT

SRVYKFCSKLAEVFEQEIDPVMQSLG

Plasmid: CREBBP (Addgene #38977)

Vector backbone: pNIC28-Bsa4 (Addgene #26103)

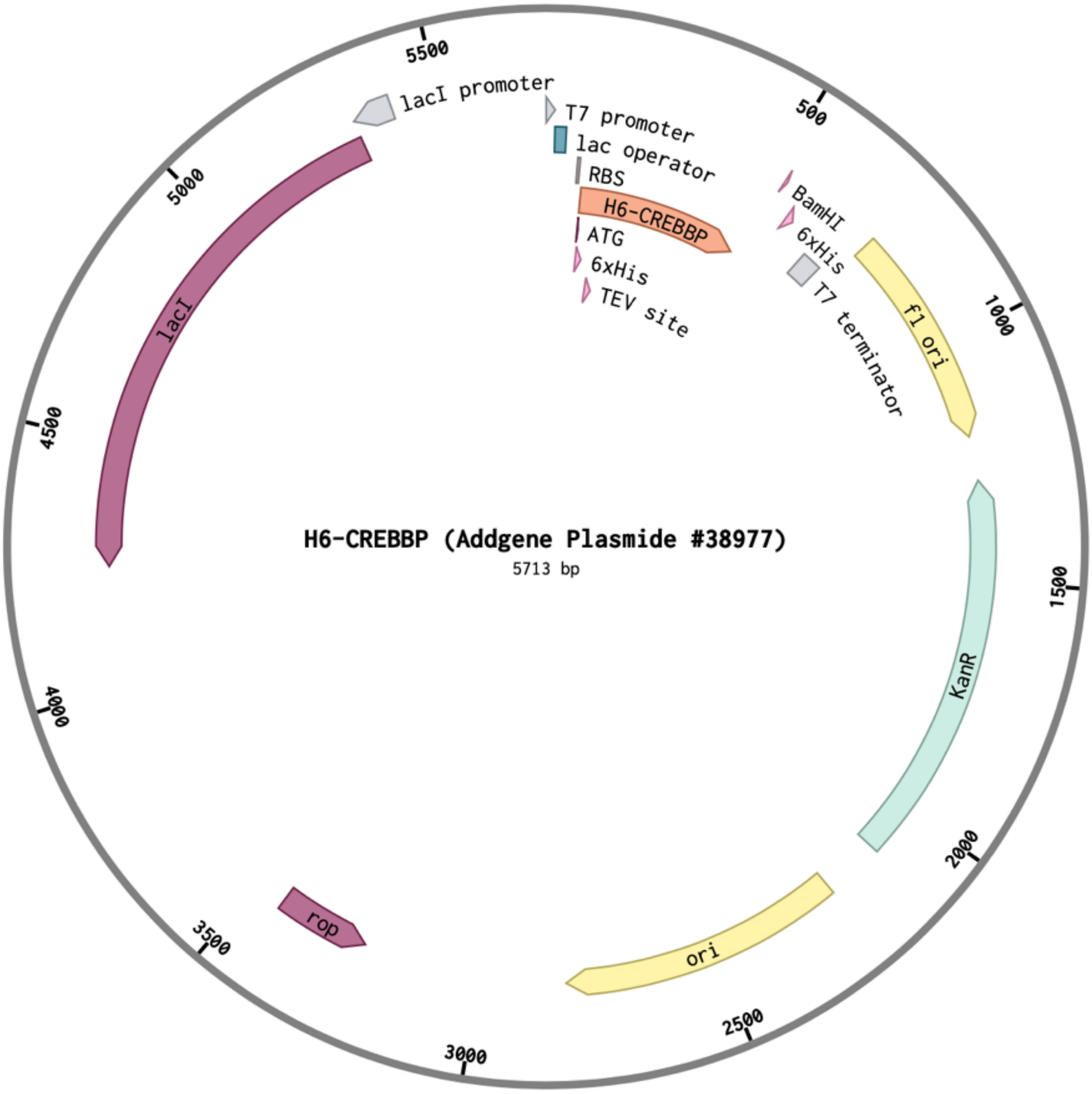

Protein Expression and Purification. The CREBBP bromodomain (Addgene plasmid # 38977) and BRD4(1) bromodomain (Addgene plasmid #38942) constructs were transformed into *E. coli* BL21 (DE3) cells for expression, as described previously.[1,14] The

proteins were purified using Immobilized Metal Affinity Chromatography (IMAC) with an HisTrapTM column (GE Healthcare) followed by gel filtration chromatography with Superdex 75 resin (GE Healthcare). The protein purity was assessed by SDS-PAGE.

Crystallization. Aliquots of the complex were set up for crystallization using a mosquito® crystallization robot (TTP Labtech, Royston UK). Coarse screens were setup onto Greiner 3-well plates using three different drop ratios of precipitant to protein per condition (100+50 nL, 75+75 nL and 50+100 nL). Initial hits were optimized further scaling up the drop sizes. All crystallizations were carried out using the sitting drop vapor diffusion method at 4 °C. CREBBP crystals with **6** were grown by mixing 100 nL of the protein (10 mg/mL and 2 mM final ligand concentration) with 200 nL of reservoir solution containing 20% PEG3350, 10% ethylene glycol and 0.2 M sodium fluoride. CREBBP crystals with **2** were grown by mixing 150 nL of protein (6.45 mg/mL) with 150 nL of reservoir solution containing 20% ethylene glycol, 21% PEG6K, 0.1 M TRIS pH 8.3 and 0.125 M LiCl. Diffraction quality crystals grew within two weeks. BRD4(1) crystals with **6** were grown by mixing 150 nL of protein (9 mg/mL and 3 mM final ligand concentration) with an equal volume of reservoir solution containing 30% PEG1000 and 0.1 M MIB pH 7.0. Initial crystals were crashed and used as seeds to obtain diffraction quality crystals which grew within a few days.

Data Collection and Structure Refinement. Crystals were cryo-protected using the well solution supplemented with additional ethylene glycol and were flash frozen in liquid nitrogen. Data were collected at Diamond beamline I03 at 0.97625 Å or I04-1 at 0.91587 Å on Pilatus3 6M detectors. Indexing and integration was carried out using XDS[15–17] and scaling was performed with SCALA.[18] Initial phases were calculated by molecular replacement with PHASER[19] using the previously determined model of CREBBP (PDB ID: 3DWY).[20] Initial models were built by ARP/wARP[21] followed by manual building in COOT.[22]

Refinement was carried out in REFMAC5.[23] Thermal motions were analyzed using TLSMD[24] and hydrogen atoms were included in late refinement cycles. Data collection and refinement statistics can be found in Tables S2 and S3. The model and structure factors have been deposited with PDB accession codes: 6YIJ (CREBBP/**2**), 6YIM (CREBBP/**6**) and 6YIN (BRD4(1)/**6**).

Single Crystal Data Collection and Structure Refinement. Single crystal X-ray diffraction data were collected using a Rigaku Oxford Diffraction SuperNova diffractometer fitted with an Oxford Cryosystems Cryostream 700 plus open flow nitrogen cooling device.[25] The CrysAlisPro software was used for data collection and integration. In general, structures were solved using SuperFlip[26] within the CRYSTALS suite.[27,28] The structures were then modified, improved and optimised by full-matrix least squares on F2. Hydrogen atoms were not generally provided by the initial solution; however, they were usually clearly visible in the difference Fourier map.

Expression of the 5FW-labeled CREBBP Bromodomain. The pNIC28-Bsa4 plasmid containing the bromodomain of CREBBP was a kind gift from Nicola Burgess-Brown (Addgene plasmid #38977; http://n2t.net/addgene:38977; RRID:Addgene_38977). The procedure for fluorinated protein expression by Gee *et al.* was followed.[29] The *E. coli* strain BL21 (DE3)-RIL was transformed with the plasmid containing the desired CREBBP bromodomain gene and plated onto an agar plate containing kanamycin and chloramphenicol. The plate was incubated overnight at 37 °C. A 5 mL LB culture containing antibiotics was inoculated using a single colony form this plate and grown overnight at 37 °C and shaking at 220 rpm. The primary culture was used to inoculate 1 L of LB media containing chloramphenicol (35 mg/L) and kanamycin (100 mg/L). This secondary culture was grown at 37 °C at 215 rpm until the optical density at 600 nm had reached 0.6-0.8. For

5FW-labeling,[29] the cells were pelleted by centrifugation and re-suspended in 1 L of defined media and 5-fluoroindole (80 mg) dissolved in dimethyl sulfoxide (DMSO, 200 µL) was added. After a recovery time of 60 min at 37 °C and 215 rpm, followed by a 30-mins cooling to the induction temperature of 20 °C, the culture was induced with 1 mM IPTG and allowed to shake for 16-20 h. Cells were pelleted by centrifugation at 8000 *g* for 10 minutes and stored at −80 °C until purification.

Site-directed mutagenesis to prepare W1151F, W1158F, and W1165F CREBBP. Site-directed mutagenesis of W1151F and W1158F on CREBBP BD was conducted using previously reported transfer-PCR procedures in 50 µL reaction mixtures.[30] Phusion High-Fidelity Master Mix (NEB), template DNA, 50 nM each of the forward primer, and T7-reverse primer (5'-GCTAGTTATGCTCAGCGG-3') was added to the reaction mixtures. Mutagenesis was confirmed by DNA Sanger sequencing.

W1151F forward primer: 5´-cagtatcaggaaccgtttcagtatgtggatgatg-3´

W1158F: 5´-tatgtggatgatgtttttctgatgtttaataatgcg-3´

Protein Purification method. To the cell pellet was added 40 mL of lysis buffer (50 mM phosphate, 300 mM NaCl, pH 7.4) and 40 mg phenylmethanesulfonyl fluoride (PMSF) and the mixture was allowed to thaw at room temperature for 30 minutes. Cells were put on ice and sonicated in 30 second intervals followed by 60 seconds of cooling for a total of 12 min sonication time. The lysed cells were centrifuged at 10 0000 *g* for 30 minutes. The supernatant was decanted from the pelleted cell debris and filtered using Whatman filters. Ni affinity purification was done using a Ni HisTrap FF 5 mL column (GE Healthcare) on an AKTA Fast Protein Liquid Chromatography (FPLC) system by monitoring the absorbance at 280 nm. Proteins were eluted with a 0-100% gradient of wash buffer (50 mM phosphate, 100 mM NaCl, 40 mM imidazole, pH 7.4) and elution buffer (50 mM phosphate, 100 mM NaCl, 400 mM imidazole, pH 7.4) across 20 column volumes. Purified protein was then

buffer exchanged into storage buffer (50 mM HEPES, 100 mM NaCl, pH 7.4) using a HiPrep desalting column (GE Healthcare) equilibrated with 1 column volume of buffer. The hexahistidine tag was removed by adding Tobacco Etch Virus (TEV) protease and incubating for 4-16 hours at 4 °C. Nickel NTA affinity resin was added, incubated for 2-24 h at 4 °C, then filtered to remove the TEV. Protein purity was assessed using SDS-polyacrylamide gel electrophoresis (12% Bis-Tris, 1.0 mM gels. Running conditions: 120 V, 90 min in MES buffer). Protein was concentrated to ~35-50 µM using Amicon Ultra-15 (Millipore) centrifugal filters with a 3 kDa molecular weight cut off (MWCO = 3000 Da), flash frozen and stored at -20°C. Quadrupole Time-of-Flight (Q-TOF) LC/MS was used to confirm the identity of the protein and determine percent fluorine incorporation using the following equation.

$$\%\, Incorporation = \frac{(0\, F\, protein * 0) + (1F\, protein * 1) + \cdots (nF\, protein * n)}{(0F\, protein * n) + (1F\, protein * n) + \cdots (nF\, protein * n)} * 100$$

Protein	Calculated m/z (Da)	Observed m/z (Da)	% Fluorine incorporation
His_6-5FW CREBBP	16699	16699	99
His_6-5FW W1151F CREBBP	16670	16669	99
His_6-5FW W1158F CREBBP	16670	16669	99

Computational Methods. Electrostatic surface potentials (ESPs) of the aniline and THQ derivative were obtained from DFT densities at the B3LYP/6-31G(d,p) level of theory (gas and solution-phase); with B3LYP/6-31G++(d,p) and M06-2X/6- 31G(d,p). Calculations used the Solvation Model Density (SMD) and SM8 Minnesota implicit solvation models of diethyl

ether (ε = 4.24) to mimic the hydrophobic environment of the binding site of CREBBP, with little effect upon the resulting ESP. ESP values were computed at 3.5 Å above the center of mass of the aromatic ring and at 3.5 Å above the substituent. These values were further summed for SAR purposes. 120 ns MD simulations were performed on the 3,4-dihydroquinoxalinone and 4,5-dihydrobenzodiazepinone ring systems using the Amber force field and following the same methodology as described in the literature.[1,31] To probe the monomeric behavior of **2** binding CREBBP, chain A was isolated from multimeric structure 6YIJ, with all crystallographic waters removed except the five water molecules found within the KAc-binding pocket and the two water molecules found in the ZA channel. Structures of **6** bound to BRD4(1) and CREBBP underwent initial preparation, with missing residues added using the WHAT-IF web interface[32] and co-crystallization factors removed. Docking models required no further preparation before MD studies. For all MD simulations, the AMBER99SB-IDLN forcefield was used for the protein.[33] Ligand parameters were derived from the General Amber forcefield (v. 1.8) from AmberTools16,[34] with ligand protonation states calculated using the Marvin Suite 16.16.6.0 from ChemAxon (https://www.chemaxon.com). The five water molecules found within the KAc-binding pocket and the two water molecules found in the ZA channel were retained from the protein structures, with all water modelled using the TIP3P model.[35] The systems were solvated in a dodecahedral box, where the minimum distance between the protein and box edge was 1.2 nm. System charges were neutralized using sodium ions, with additional NaCl ions added to form an overall salt concentration of 150 nM. All systems then underwent initial energy minimization using the steepest decent algorithm to remove poor contacts, which had a maximum force cut off of 1000 kJ mol^{-1}. The systems then underwent further equilibration in the isothermal-isobaric ensemble for 200 ps. The temperature was coupled using a Langevin thermostat, with a target temperature of 298.15 K, and the pressure was coupled using the Berendsen weak coupling algorithm to a target pressure of 1 atm.[36,37]

Simulations of **2** in CREBBP were performed using GROMACS 2016.3, while all other simulations were performed using GROMACS 2018.2.[38] All simulations underwent 50 ns of production MD, in triplicate. Analysis was performed using the MDAnalysis package for Python,[39] all rolling averages were performed over 50 2 fs timesteps.

Molecular Docking. The protein model for CREBBP was extracted from the structure of **6** bound to CREBBP (PDB: 6YIM), while a protein model for BRD4(1) was extracted from PDB:6CZV. All binding site waters were retained in the protein model, which was protonated at pH 7.4 using the MOE (v. 2018.2) software package. Molecular docking of **2**, **25** and **26** to CREBBP and BRD4(1) was performed using AutoDock4,[40] where 100 poses were generated and clustered using a 2 Å cut-off. In each case of docking to CREBBP, the lowest energy pose of each ligand cluster corresponded to an analogous binding mode to **6** in CREBBP. These poses were used for subsequent MD studies. For BRD4(1) docking, poses that were analogous to the binding mode of **6** in BRD4(1) were selected for further MD studies.

SEC Dimerization Experiment. The dimeric state of the CREBBP bromodomain in solution was analyzed by gel filtration in a buffer containing 50 mM HEPES, pH 7.6, and 500 mM NaCl using a HiLoad® 16/600 Superdex® 75 column (GE Healthcare). Protein (50 µM) and ligand **2** (50 µM) or DMSO were mixed and incubated at 20 °C for 30 min before injection. Eluting peaks were monitored using ultraviolet absorbance at 280 nm.

AlphaScreen™. Bromodomain AlphaScreen™ assays were carried out as previously described.[14,41] The buffer (25 mM HEPES, 100 mM NaCl, 0.05% *w/v* CHAPS, 0.1% *w/v* BSA; pH 7.6) was filter sterilized through a 0.22 µm filter. For incubation steps, the plate was sealed, shaken for 10 seconds at 600 rpm on a plate oscillator and kept at room temperature

in the dark. Compounds, protein, and peptides were dispensed into a ProxiPlate-384 Plus (Perkin Elmer) using a Thermo-Fisher electronic multi-channel pipette. The plate was read using an Envision 2104 Multilabel Plate Reader (Perkin Elmer) using the built-in AlphaScreen™ 384 ProxiPlate function: excitation 680 nm, 0.18 sec; emission 570 nm, 0.37 sec. Ni^{2+} chelate acceptor and streptavidin donor beads were prepared as a mixture in a 1:300 dilution (0.007 mg/mL final assay concentration (FAC)). Compounds were dispensed into wells as serial dilutions from the specified concentrations from 50 mM DMSO stock solutions. Protein-peptide mixes were prepared then dispensed into wells. Assay beads were then added. Dose response curves were obtained for compounds in triplicate, with serial 1:2 dilutions. Data were processed by fitting a four-parameter equation to calculate IC_{50} values using Prism software. For TruHits Experiments, compounds (5 μL) were dispensed into wells as serial 1:2 dilutions from the specified concentrations from 50 mM DMSO stock solutions. Assay beads (15 μL) were pre-incubated for 30 minutes and added to the first plate attain a final assay volume of 20 μL, and then incubated for a further 10 minutes. To the second plate, streptavidin donor beads (7.5 μL) were added to the compounds and incubated for 30 minutes. Biotinylated acceptor beads (7.5 μL) were then added to the second plate to attain a final assay volume of 20 μL, and then incubated for a further 30 minutes. Data were processed by calculating the percentage decrease in emission intensity with respect to the average of the DMSO control wells on each respective plate, which were set as 100%.

Ligand-Observed Proton NMR. BRD4(1) bromodomain protein in HEPES containing storage buffers were exchanged into Invitrogen 10 mM Na/K phosphate buffer (0.9% NaCl, pH 7.6) using dialysis. BRD4(1) bromodomain protein concentrations in the phosphate buffer were calculated from by measuring the absorbance at 280 nm using a NanoDrop Lite spectrophotometer (Nanodrop® Technologies Inc.) by using the predicted protein

absorbance (ε_{280}: 28420 M^{-1} cm^{-1}). Samples were prepared to a total sample volume of 160 µL phosphate buffer, with 50 µM compound from 5 mM D_6-DMSO stock solutions, 10% D_2O for signal locking and the appropriate amount of protein. After loading into 3 mm Hildenberg NMR tubes for Bruker Match™ system, samples were briefly centrifuged in a manual benchtop rotor. Spectra were recorded on a Bruker AVIII 700 MHz spectrometer with a TXI probe using a standard excitation-sculpting water suppression pulse sequence (256 or 512 scans each). For each compound, a compound only sample was recorded as a reference, followed by samples with protein. Each spectrum was processed with Bruker Topspin™ 3.2, the data were recorded using Microsoft Excel®, then the K_d value was calculated using the "one site – specific binding; non-linear regression" function using Graphpad Prism®. In Topspin, spectra were phased corrected and calibrated to the residual DMSO peak, then local baseline corrections were performed ("absf n" function). The integral of the studied peaks and the DMSO reference peaks were calculated in each spectra for the determined chemical shift range (constant ranges were used for each studied compound). Using the ratio of integrals, the integral decrease of the peak of interest with increasing protein concentration was plotted for each compound.

Protein-Observed Fluorine NMR (PrOF NMR). All experiments were run on a Bruker Avance III HD 500 with a 5 mm Prodigy TCI inverse cryoprobe (^{19}F S:N 2000:1). The 5FW-labeled CREBBP bromodomain was diluted in 50 mM HEPES, 100 mM NaCl, pH=7.4 buffer by the addition of D_2O and 0.1% TFA to final concentrations of 5% and 0.05%, respectively. Two one-dimensional ^{19}F NMR spectra were taken of the control protein sample at an O1P of -75 ppm, NS=16, D1=1, AQ=0.5 (TFA Reference set to −75.25 ppm) and an O1P of −125 ppm, NS=750-1000, D1=0.6, AQ=0.05s (protein resonances). Ligand stock solutions of 100 mM were prepared in D_6-DMSO and titrated into the bromodomain protein solution kept at a constant concentration (35-50 µM). The change in chemical shift of each protein

resonances ($\Delta\delta_{obs}$) was then compared to the spectrum of the fluorinated bromodomain in the absence of ligand.

Isothermal Titration Calorimetry. All calorimetric experiments were performed on a MicroCal iTC200 or MicroCal PEAQ-ITC Automated (Malvern) and analyzed with the MicroCal PEAQ-ITC Analysis software (Malvern 1.1.0.1262) using a single binding site model, with the N number set to 1. The first data point was excluded from the analysis. BRD4(1) and CREBBP bromodomains were dialyzed at 4 °C overnight in a Slide-A-Lyzer® MINI Dialysis Device (2000 MWCO; Thermo Scientific Life Technologies) into 50 mM HEPES, 150 mM NaCl containing 0.2% DMSO; pH 7.4. Proteins were centrifuged to remove aggregates (3 min, 3,000 rpm, 25 °C). Protein concentration were determined by measuring the absorbance at 280 nm using a NanoDrop Lite spectrophotometer (Nanodrop® Technologies Inc.) by using the predicted protein absorbance (CREBBP: ε_{280}: 26930 M^{-1} cm^{-1} and BRD4(1): ε_{280}: 28420 M^{-1} cm^{-1}). Small molecules ligands were dissolved as 10 to 50 mM DMSO stock solution and diluted to the required concentration using dialysis buffer. The cell was stirred at 750 rpm, reference power set to 5 µcal/sec and temperature held at 25 ˚C. After an initial delay of 60 sec, 20 × 2 µL injections (first injection 0.4 µL) were performed with a spacing of 150 sec. Heated dilutions were measured under the same conditions and subtracted for analysis. Small molecule solutions in the calorimetric cell (250 µL, (10 to 40 µM)) were titrated with the protein solutions in the syringe (60 µL, 160 to 220 µM).

Cell line. Human colorectal carcinoma HCT116 cells from ATCC collection were cultured in DMEM (Sigma-Aldrich, UK) supplemented with 10% FBS, penicillin (100 U/mL) and streptomycin (100 µg/mL at 37 °C in a 5% CO_2 humidified environment. Cells were routinely screened for the presence of mycoplasma and found to be negative.

Immunoblotting. Cells were seeded at a density of 1 × 10^6 cells/dish in 100-mm Petri dishes overnight. Following compound treatment, cells were collected and lysed in urea buffer (9 M urea, 75 mM Tris-HCl pH 7.5, 0.15 M β-mercaptoethanol). Proteins (50 μg) were separated by gel electrophoresis on a 4-20% Tris-glycine gel and subjected to immunoblotting. The following primary antibodies were used: CREBBP (7389S Cell Signaling Technology), c-MYC (5605S Cell Signaling Technology), p53 (DO-I, Santa Cruz), H3AcK18 (13998S Cell Signaling Technology), H3 total (3638S Cell Signaling Technology), H3AcK18β-actin (AC-15, Santa Cruz). Secondary antibodies IRDye® 680RD Goat anti-Mouse IgG (H+L) and IRDye® 680RD Goat anti-Rabbit IgG (H+L) were from LI-COR Biosciences. The Odyssey infrared system (LI-COR) was used to image Western blots.

RNA extraction and qPCR. Cells were seeded at a density of 1 × 10^6 cells/dish in 100-mm Petri dishes overnight. Following compound treatment, cells were collected, and RNA was isolated using TRIzol (Invitrogen/Life Technologies) according to the manufacturer's instructions. For qPCR expression analysis, cDNA was reverse transcribed from total RNA using the Verso Kit (Thermo Scientific). qPCR was performed using the SYBR Green PCR Master Mix Kit (Applied Biosystems) in a 7500 FAST Real-Time PCR thermocycler with v2.0.5 software (Applied Biosystems). The following sequences of qPCR primers were used: c-MYC For: CCCTCCACTCGGAAGGACTA, c-MYC Rev: GCTGGTGCATTTTCGGTTGT, 18S For: TAGAGGGACAAGTGGCGTTC, 18S Rev: CGGACATCTAAGGGCATCAC. The c-MYC mRNA fold change was calculated using a $2^{-\Delta\Delta Ct}$ method and expressed in relation to the 18S reference gene. The qPCR graph shown represents the mean ± SD of 3 biological replicates.

Table S1. Biophysical data and calculated properties for all tested compounds.

Number	Structure	AlphaScreen IC_{50}/µM CREBBP	ITC K_d/µM CREBBP	ITC K_d/µM BRD4(1)	NMR K_d/µM BRD4(1)	pK_d or pIC_{50} CREBBP (A)	LE	cLogP	LLE (A – B)	$cLogD_{pH7.4}$ (B)	SFI
1[a]	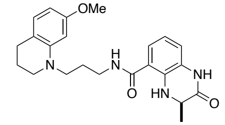	0.323*	0.353 ± 0.06 / 0.390*	1.01 ± 0.30 / 1.40*	n.t.	6.0 (pK_d)	0.28	3.63	2.86	3.14	5.14
6	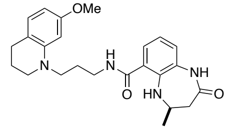	1.4 ± 0.2	0.466 ± 0.03 (n=2 ± s.d.)	3.04 ± 0.79	5.80 ± 0.99	6.3 (pK_d)	0.28	4.06	2.60	3.70	5.7
7	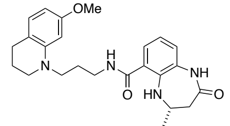	>50	n.b.	n.t.	n.t.	-	-	4.06	-	3.70	5.7
8	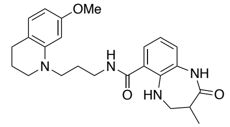	10.2 ± 0.5	n.t.	n.t.	n.t.	5.0 (pIC_{50})	0.23	4.06	1.3	3.70	5.7
2	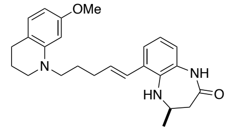	n.t.	0.102 ± 0.01 (n=5 ± s.d.)	n.b.	10.3 ± 1.89	7.0 (pK_d)	0.33	4.70	1.92	5.08	7.08
3	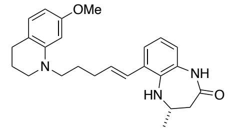	n.t.	n.b.	n.t.	n.t.	-	-	4.70	-	5.08	7.08
25	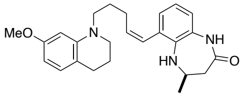	n.t.	0.329 ± 0.08	1.41 ± 0.21	2.30 ± 0.41	6.5 (pK_d)	0.30	4.70	1.42	5.08	7.08
26	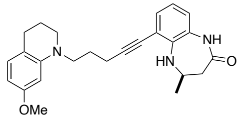	n.t.	0.154 ± 0.03 (n=5 ± s.d.)	n.b.	n.t.	6.8 (pK_d)	0.32	4.85	2.02	4.78	6.78

**27**	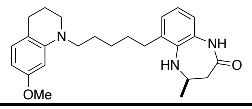	n.t.	n.b.	n.t.	n.t.	-	-	5.14	-	5.55	7.55

[a]Compounds previously published by our group.[1] n.t.: not tested; n.b.: no binding.*Previously published value.

Table S2. Data collection and refinement statistics for the X-ray crystal structures of **2** and **6** bound to the CREBBP bromodomain and **6** bound to BRD4(1).

**Data Collection**			
PDB ID	**6YIM**	**6YIJ**	**6YIN**
Protein/Ligand	CREBBP/**6**	CREBBP/**2**	BRD4(1)/**6**
Space group	C2	$P4_32_12$	$P2_12_12_1$
Cell dimensions: a, b, c (Å)	94.38 34.68 40.05	96.69 96.69 251.71	41.60 48.35 58.96
α, β, γ (deg)	90.00 107.18 90.00	90.00 90.00 90.00	90.00 90.00 90.00
Resolution* (Å)	1.23 (1.30-1.23)	2.20 (2.32-2.20)	1.52 (1.61-1.52)
Unique observations*	36020 (5196)	61638 (8823)	18722 (2686)
Completeness* (%)	99.7 (99.3)	99.9 (100.0)	99.9 (100.0)
Redundancy*	2.9 (2.8)	12.7 (12.6)	6.3 (6.7)
Rmerge*	0.052 (0.059)	0.160 (1.438)	0.049 (0.746)
I/ σI*	20.7 (13.7)	11.1 (2.0)	15.1 (2.0)
**Refinement**			
Resolution (Å)	1.23	2.20	1.52
R_{work} / R_{free} (%)	14.1/16.7	20.4 / 26.9	20.6/24.5
Number of atoms (protein/other/water)	980/163/31	6743 / 210 / 224	1047/31/73
B-factors (Å^2) (protein/other/water)21.85	11.98/24.26/13.68	48.77 / 42.08 / 38.44	30.18/32.86/35.35
r.m.s.d bonds (Å)	0.014	0.015	0.016
r.m.s.d angles (°)	1.750	2.171	1.543
Ramachadran Favoured (%)	99.08	99.00	97.54
Allowed (%)	0.92	1.00	2.46
Disallowed (%)	0.00	0.00	0.00

* Values in parentheses correspond to the highest resolution shell.

NMR spectra of novel compounds

^{1}H NMR (*R*)-*N*-(3-(7-Methoxy-3,4-dihydroquinolin-1(2*H*)-yl)propyl)-3-methyl-2-oxo-1,2,3,4-tetrahydroquinoxaline-5-carboxamide

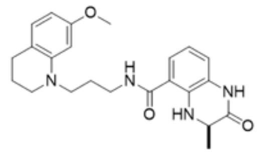

1

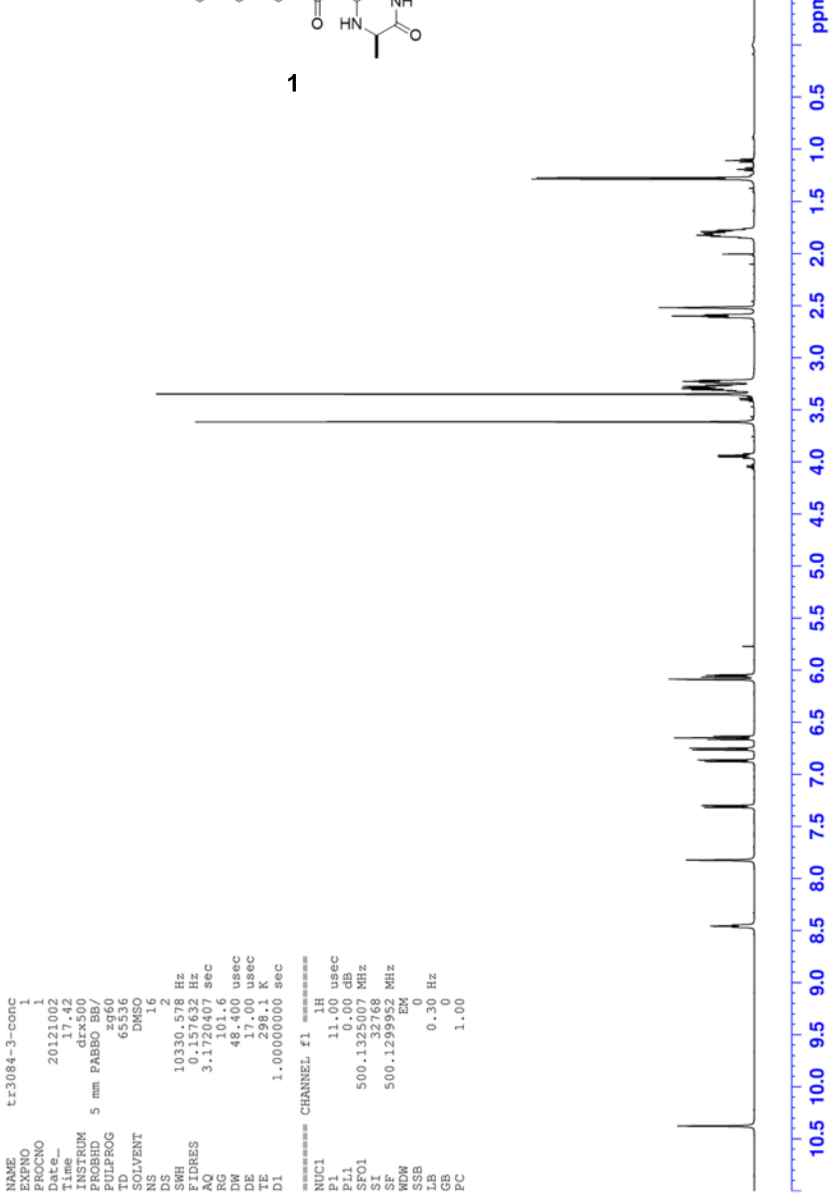

^{1}H NMR (*R*)-Benzyl 2-(4-*tert*-butoxy-4-oxobutan-2-ylamino)-3-nitrobenzoate

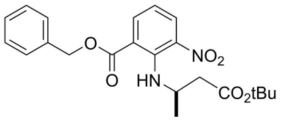

18

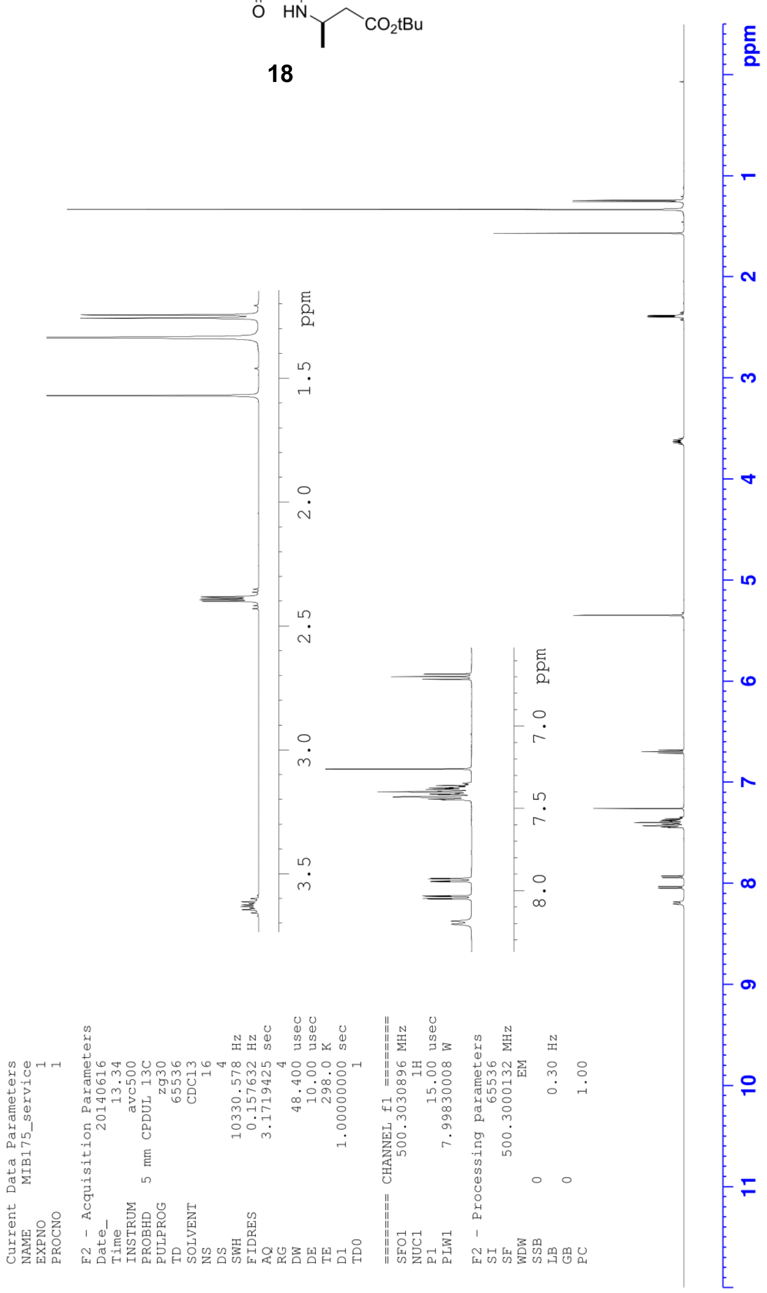

^{13}C NMR (*R*)-Benzyl 2-(4-*tert*-butoxy-4-oxobutan-2-ylamino)-3-nitrobenzoate

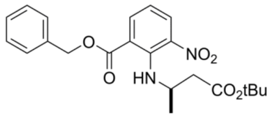

18

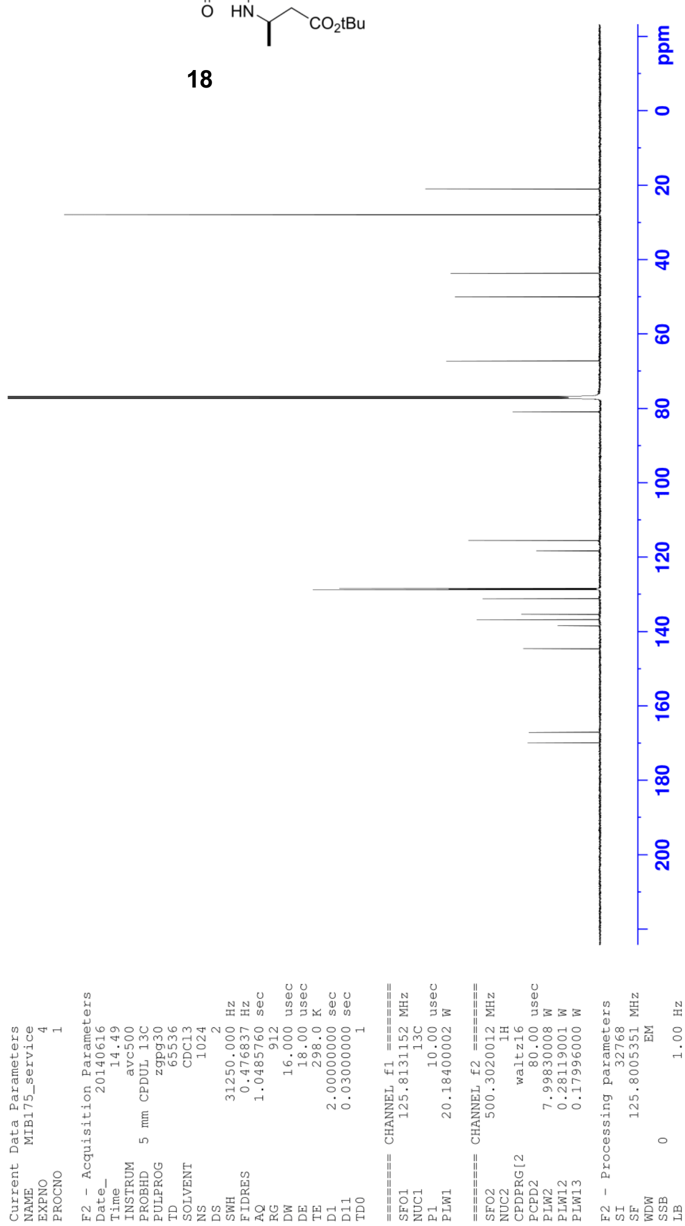

^{1}H NMR (*R*)-Benzyl 4-methyl-2-oxo-2,3,4,5-tetrahydro-1*H*-benzo[*b*][1,4]diazepine-6-carboxylate

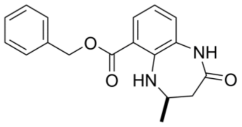

20

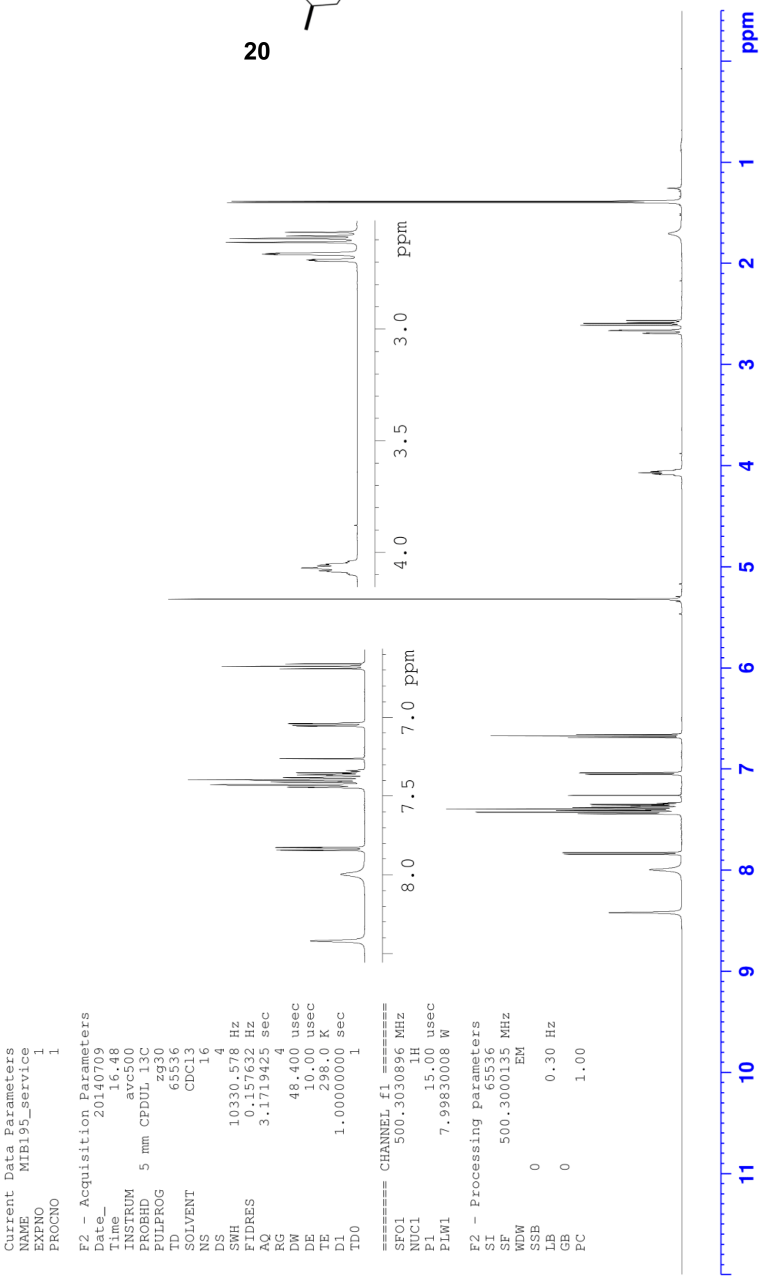

^{13}C NMR (*R*)-Benzyl 4-methyl-2-oxo-2,3,4,5-tetrahydro-1*H*-benzo[*b*][1,4]diazepine-6-carboxylate

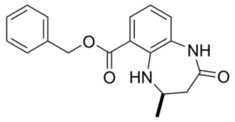

20

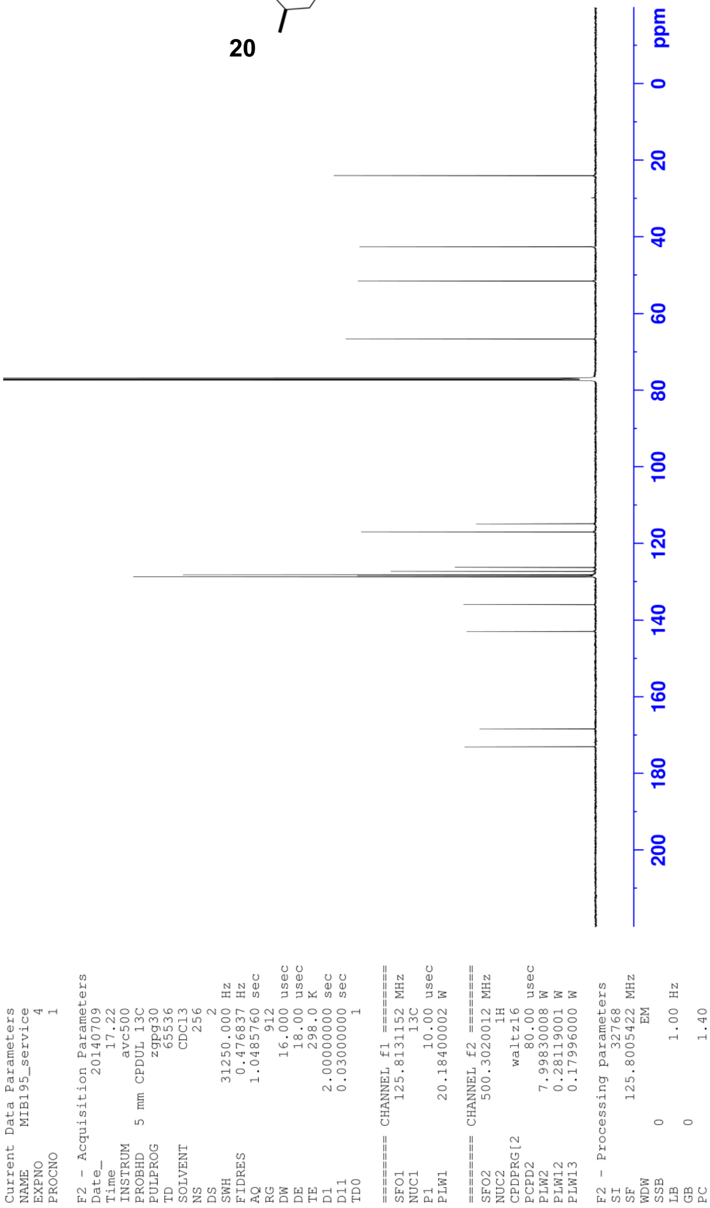

^{1}H NMR (*R*)-3-(7-Methoxy-3,4-dihydroquinolin-1(2*H*)-yl)propyl 4-methyl-2-oxo-2,3,4,5-tetrahydro-1*H*-benzo[*b*][1,4]diazepine-6-carboxylate

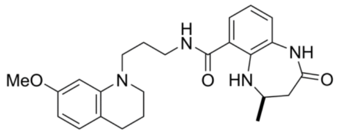

6

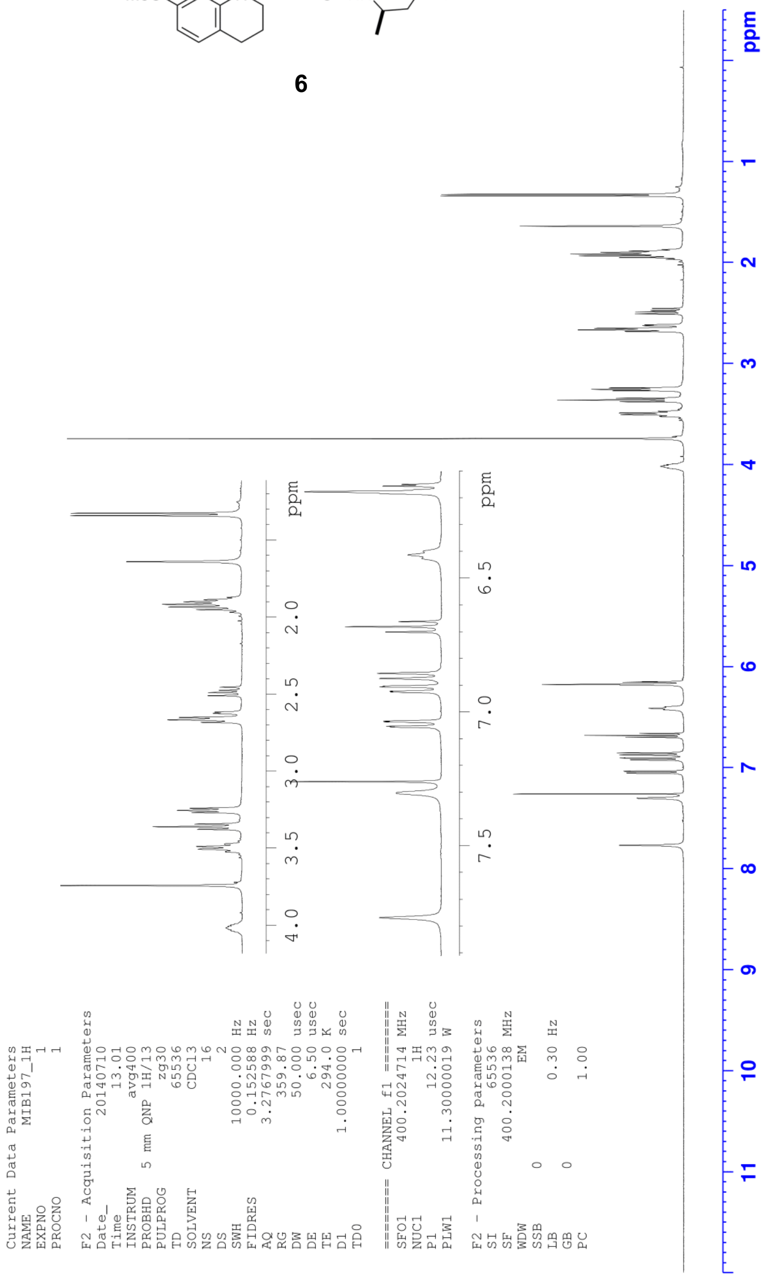

^{13}C NMR (*R*)-3-(7-Methoxy-3,4-dihydroquinolin-1(2*H*)-yl)propyl 4-methyl-2-oxo-2,3,4,5-tetrahydro-1*H*-benzo[*b*][1,4]diazepine-6-carboxylate

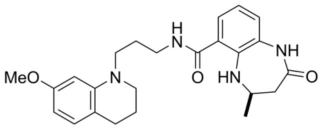

6

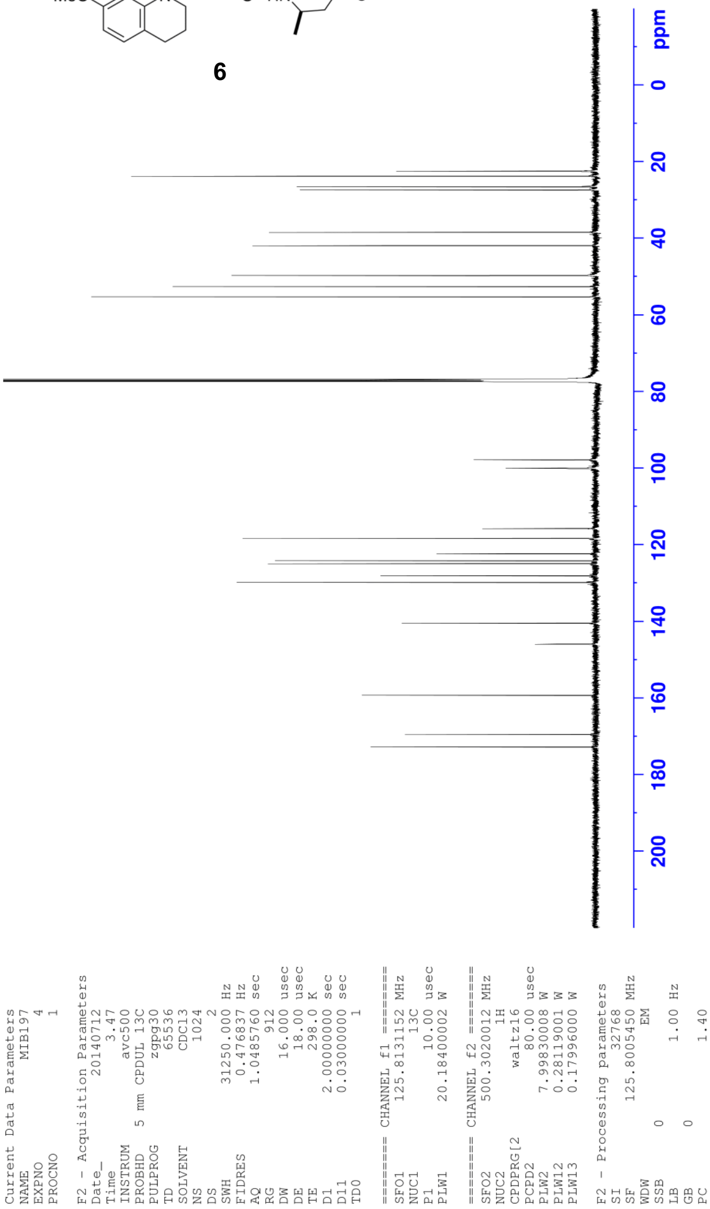

^{1}H NMR (*S*)-3-(7-Methoxy-3,4-dihydroquinolin-1(2*H*)-yl)propyl 4-methyl-2-oxo-2,3,4,5-tetrahydro-1*H*-benzo[*b*][1,4]diazepine-6-carboxylate

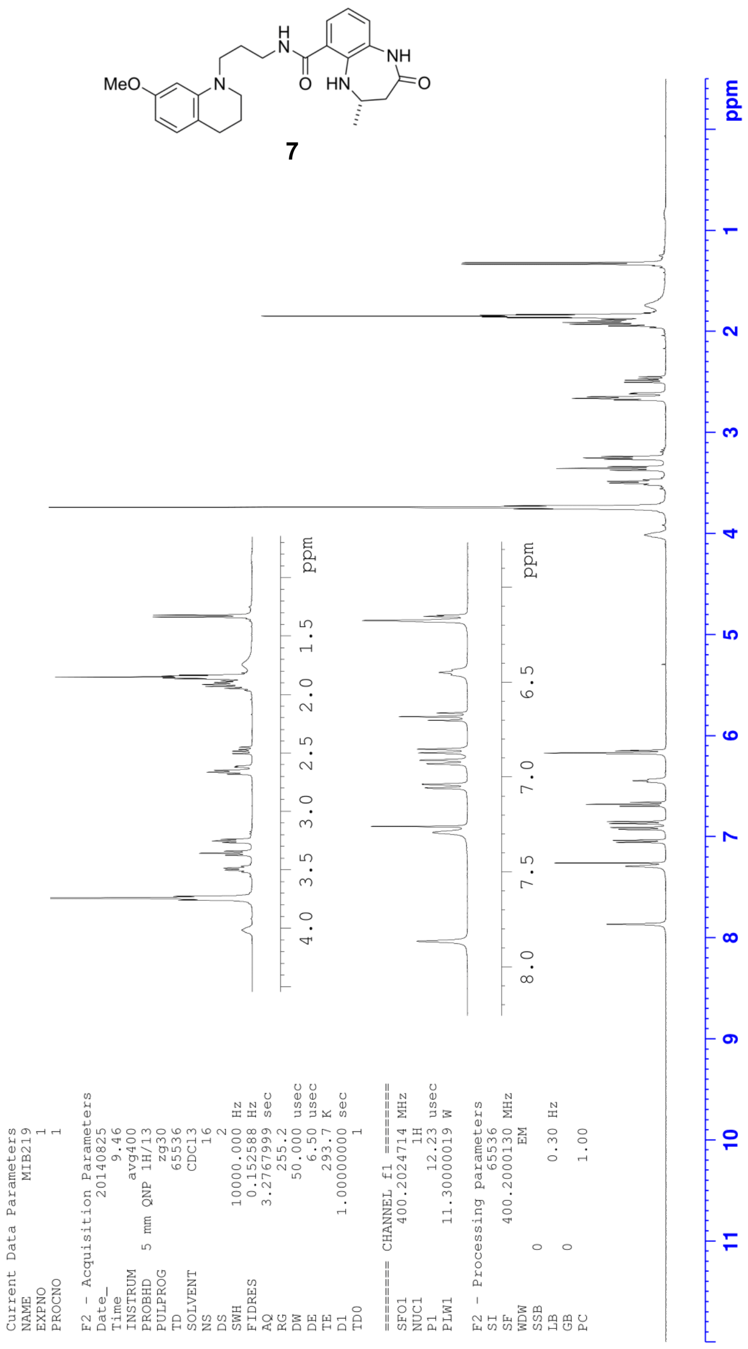

^{1}H NMR Benzyl 2-(3-*tert*-butoxy-2-methyl-3-oxopropylamino)-3-nitrobenzoate

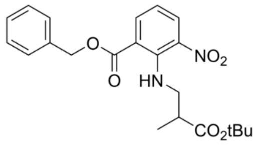

22

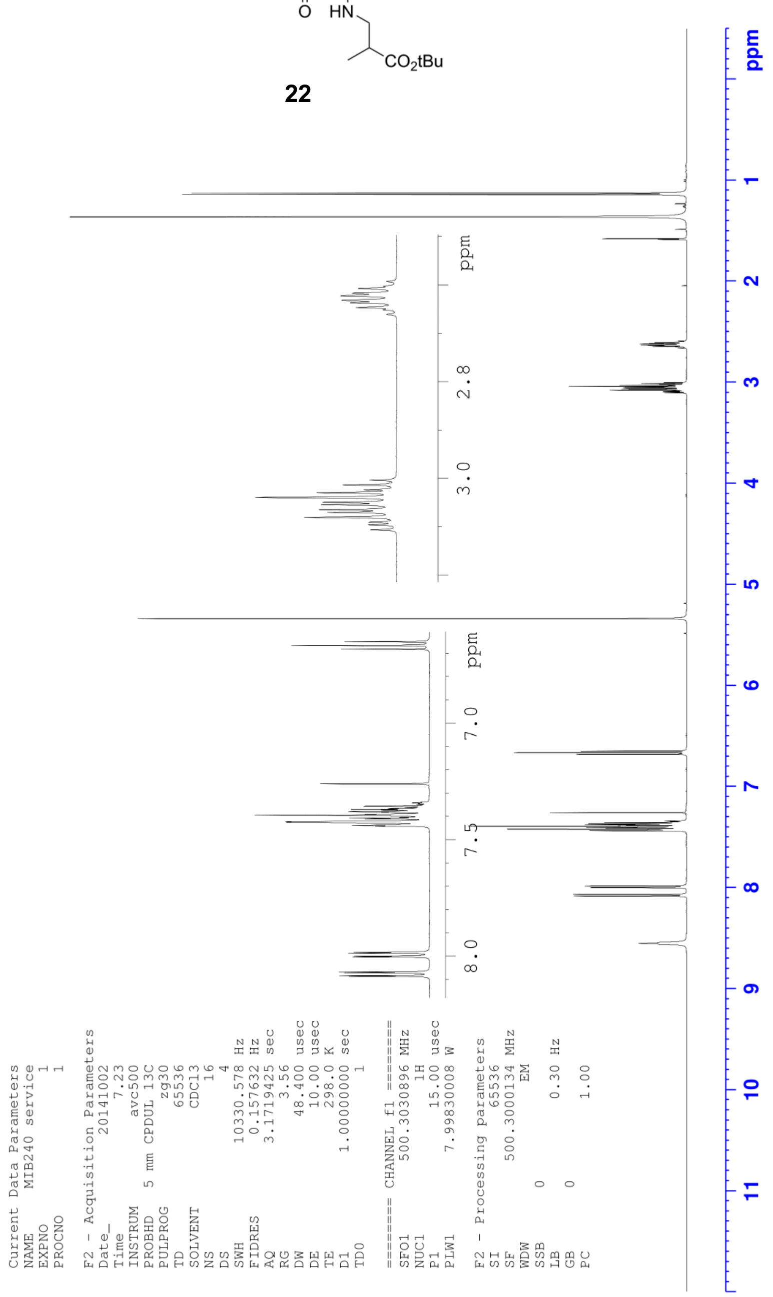

^{13}C NMR Benzyl 2-(3-*tert*-butoxy-2-methyl-3-oxopropylamino)-3-nitrobenzoate

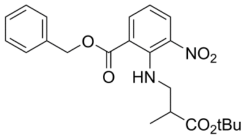

22

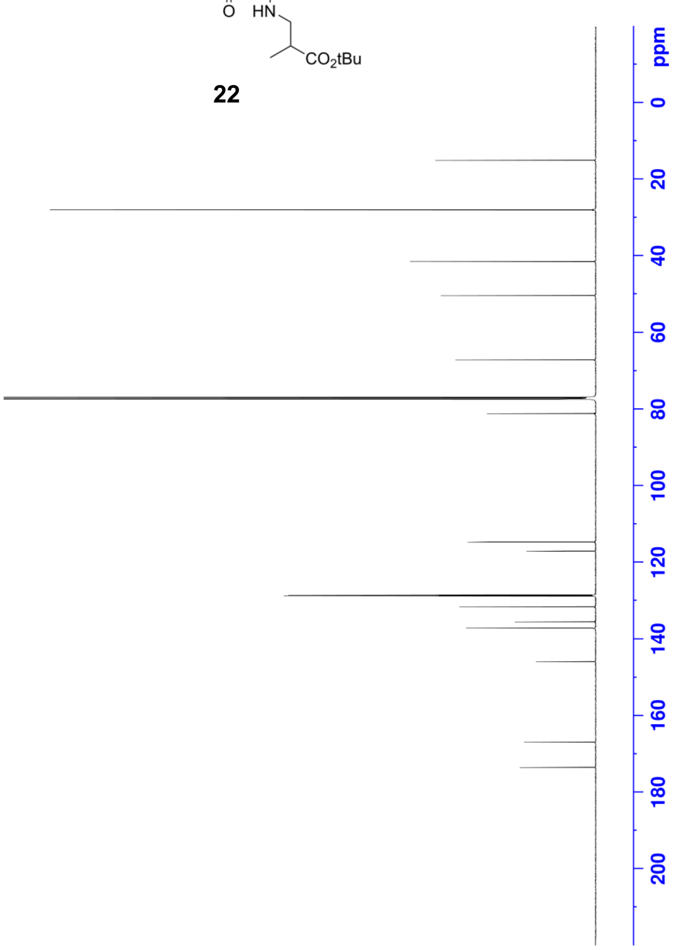

Current Data Parameters
NAME MIB240 service
EXPNO 4
PROCNO 1

F2 - Acquisition Parameters
Date_ 20141002
Time 8.37
INSTRUM avc500
PROBHD 5 mm CPDUL 13C
PULPROG zgpg30
TD 65536
SOLVENT CDCl3
NS 1024
DS 2
SWH 31250.000 Hz
FIDRES 0.476837 Hz
AQ 1.0485760 sec
RG 912
DW 16.000 usec
DE 18.00 usec
TE 298.0 K
D1 2.00000000 sec
D11 0.03000000 sec
TD0 1

======== CHANNEL f1 ========
SFO1 125.8131152 MHz
NUC1 13C
P1 10.00 usec
PLW1 20.18400002 W

======== CHANNEL f2 ========
SFO2 500.3020012 MHz
NUC2 1H
CPDPRG[2 waltz16
PCPD2 80.00 usec
PLW2 7.99830008 W
PLW12 0.28119001 W
PLW13 0.17996000 W

F2 - Processing parameters
SI 32768
SF 125.8005351 MHz
WDW EM
SSB 0
LB 1.00 Hz
GB 0
PC 1.40

^{1}H NMR Benzyl 3-methyl-2-oxo-2,3,4,5-tetrahydro-1*H*-benzo[*b*][1,4]diazepine-6-carboxylate

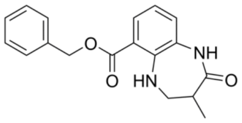

23

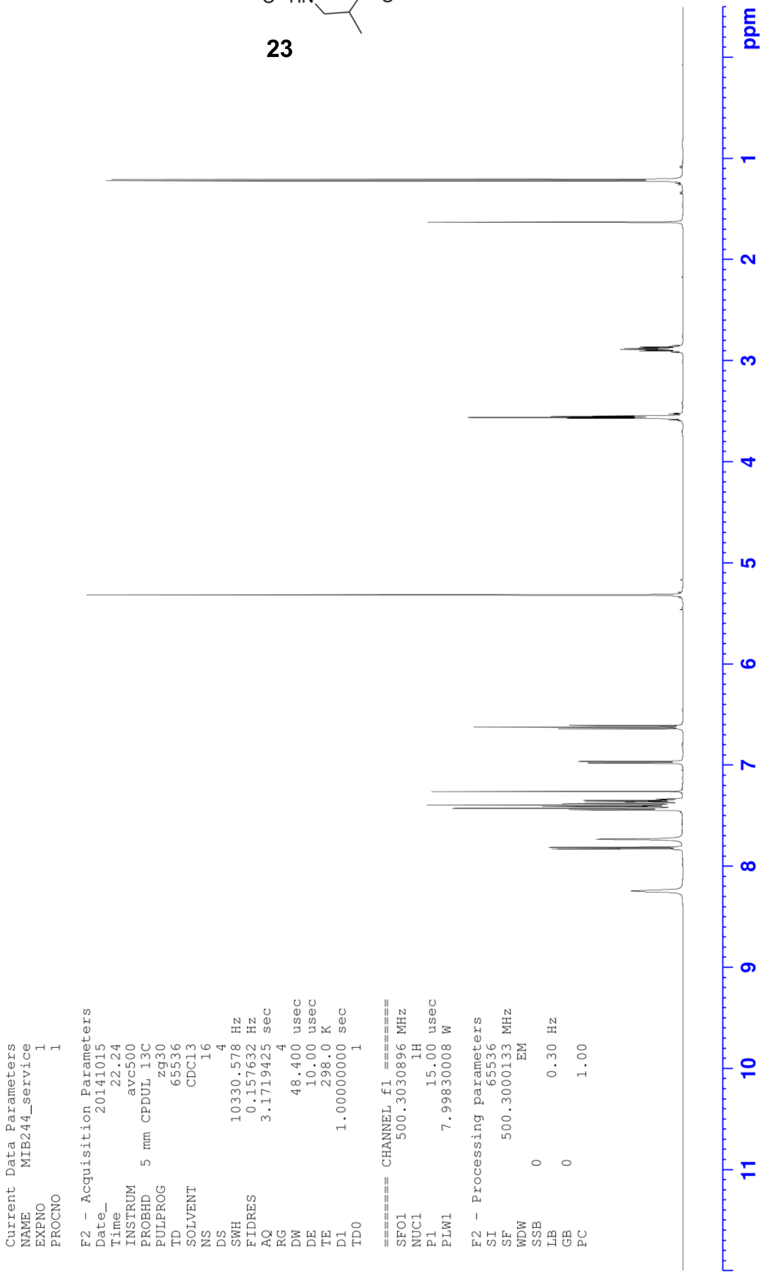

^{13}C NMR Benzyl 3-methyl-2-oxo-2,3,4,5-tetrahydro-1*H*-benzo[*b*][1,4]diazepine-6-carboxylate

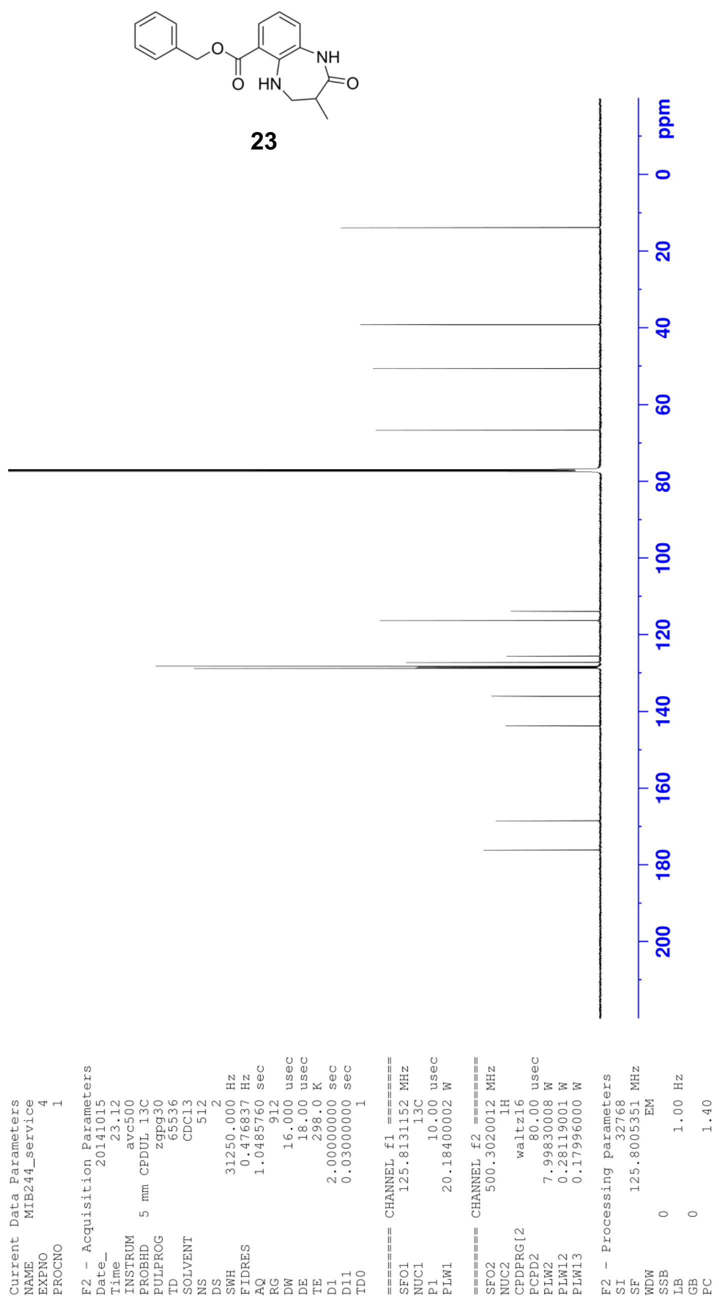

^{1}H NMR *N*-(3-(7-Methoxy-3,4-dihydroquinolin-1(2*H*)-yl)propyl)-3-methyl-2-oxo-2,3,4,5-tetrahydro-1*H*-benzo[b][1,4]diazepine-6-carboxamide

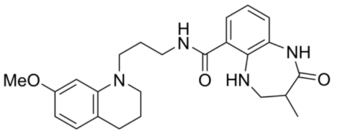

8

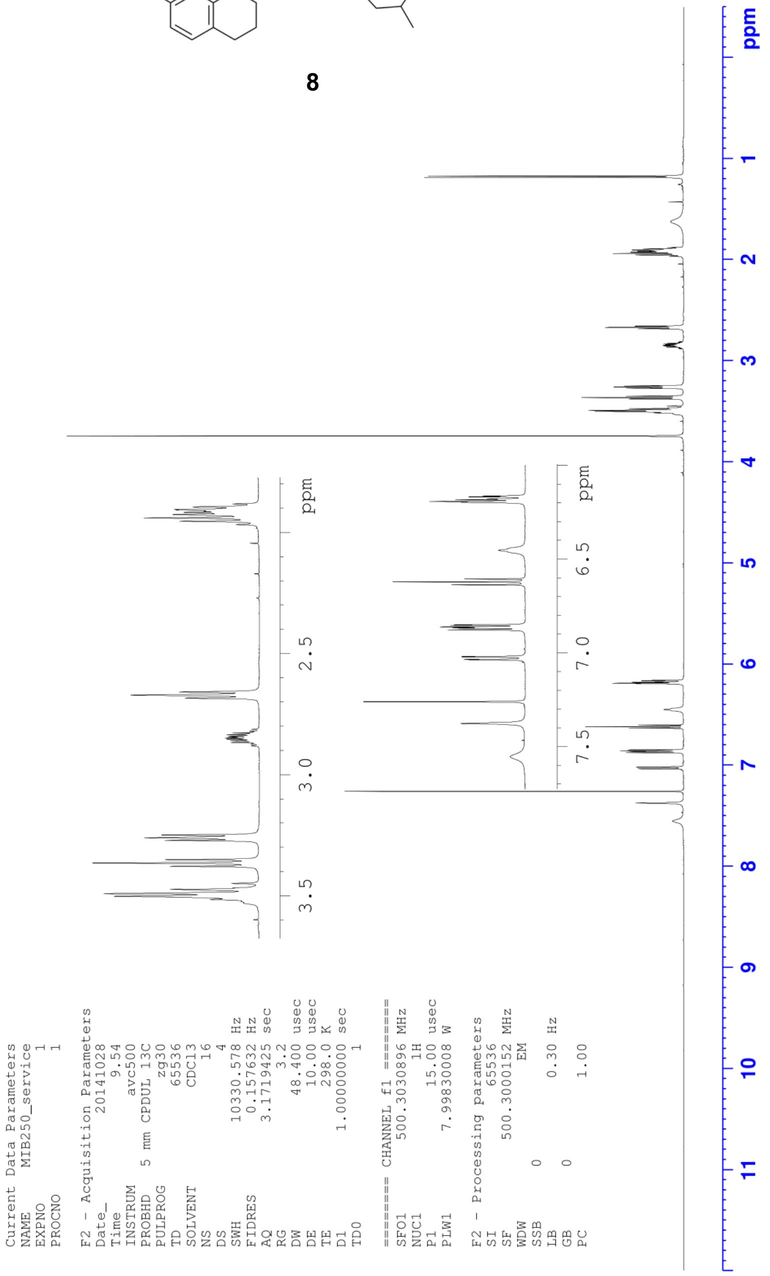

^{13}C NMR *N*-(3-(7-Methoxy-3,4-dihydroquinolin-1(2*H*)-yl)propyl)-3-methyl-2-oxo-2,3,4,5-tetrahydro-1*H*-benzo[b][1,4]diazepine-6-carboxamide

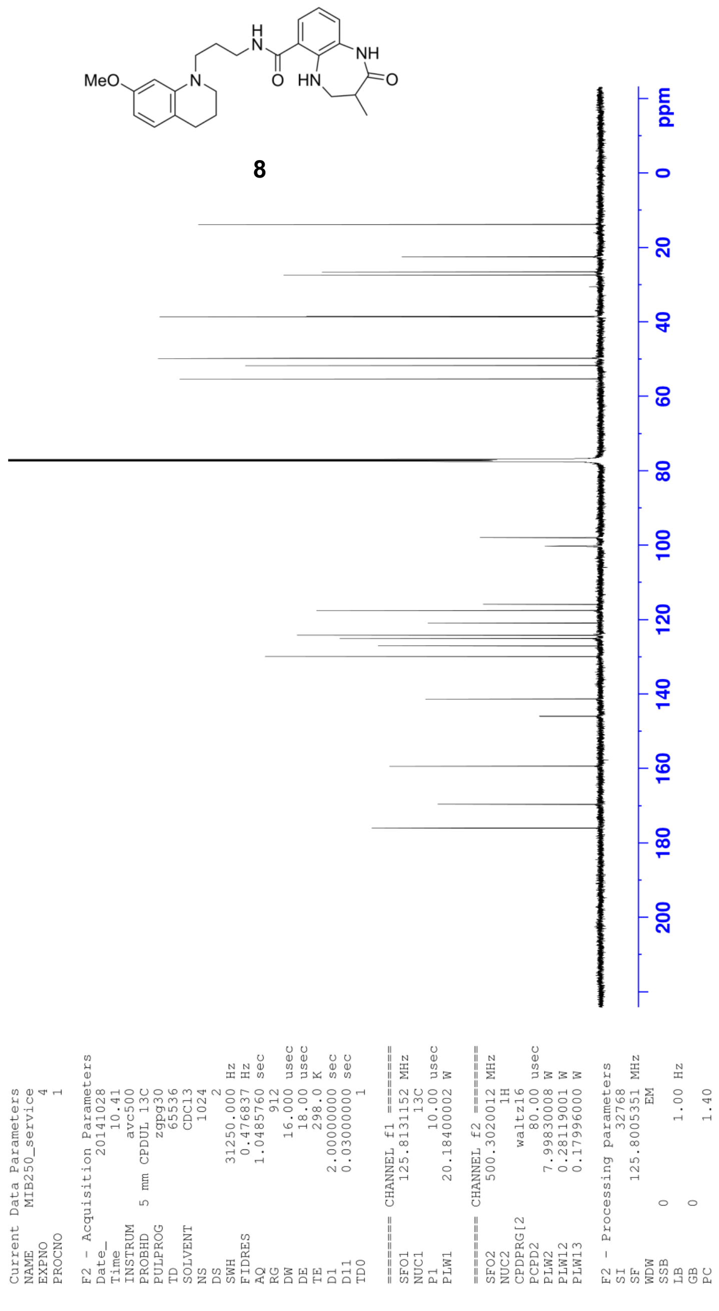

^{1}H NMR *tert*-Butyl (*S*)-3-((2-bromo-6-nitrophenyl)amino)butanoate

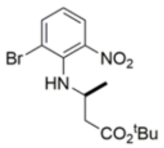

30

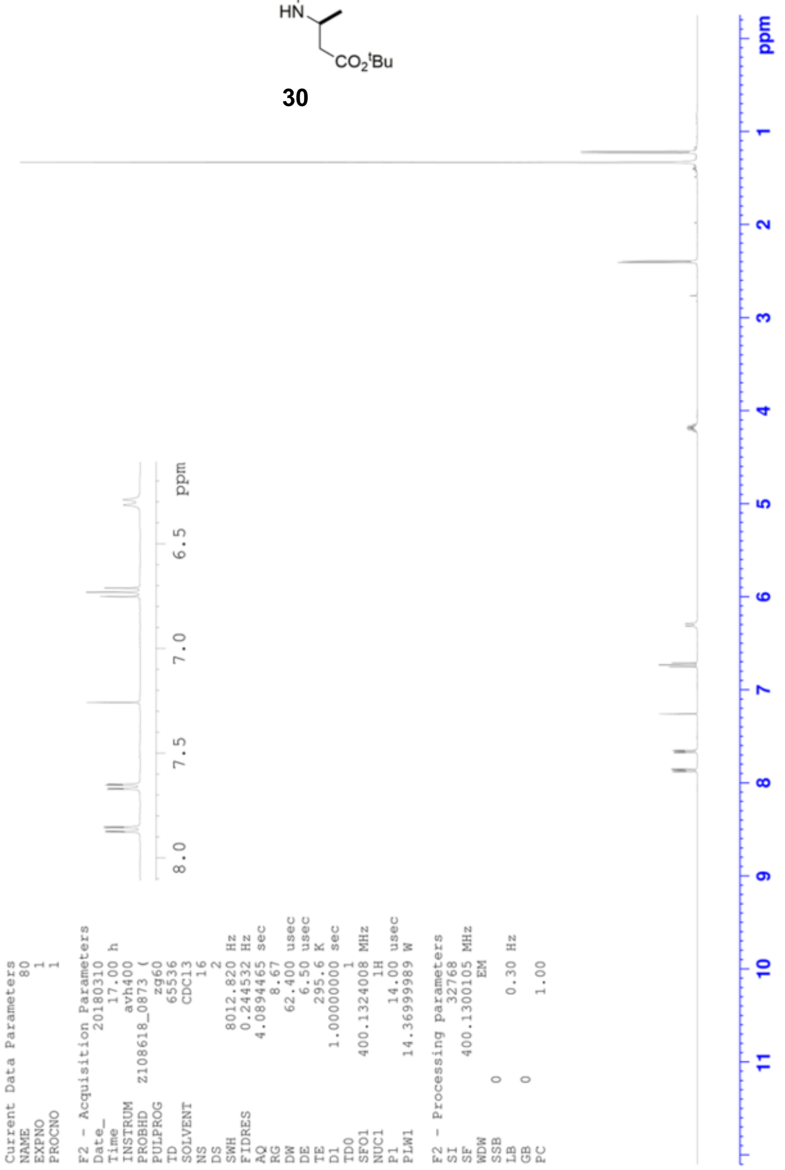

^{13}C NMR *tert*-Butyl (*S*)-3-((2-bromo-6-nitrophenyl)amino)butanoate

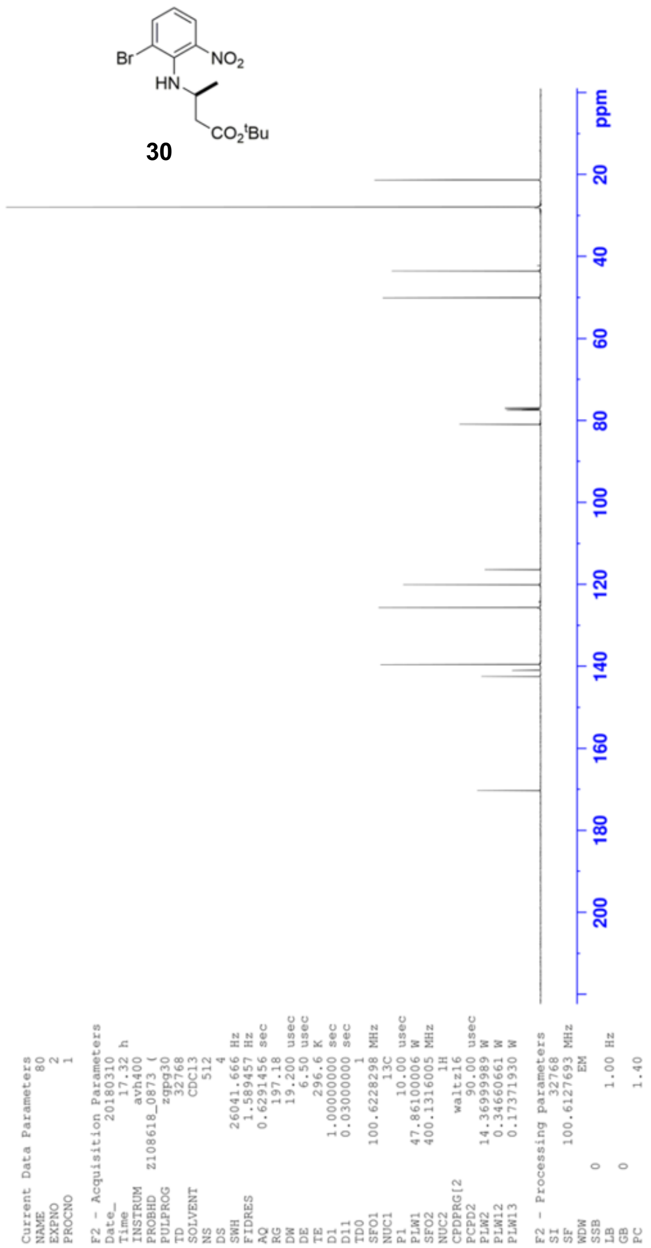

^{1}H NMR (*R*)-6-Bromo-4-methyl-1,3,4,5-tetrahydro-2*H*-benzo[*b*][1,4]diazepin-2-one

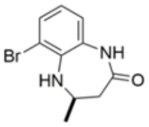

31

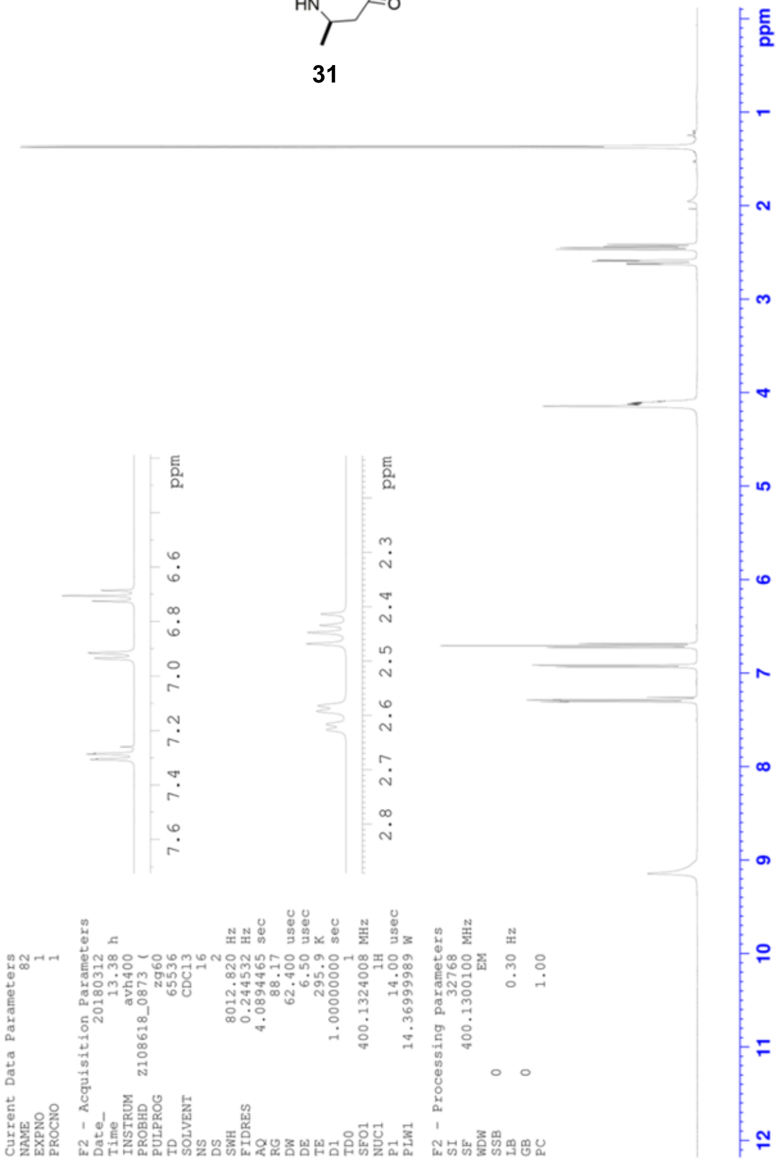

^{13}C NMR (*R*)-6-Bromo-4-methyl-1,3,4,5-tetrahydro-2*H*-benzo[*b*][1,4]diazepin-2-one

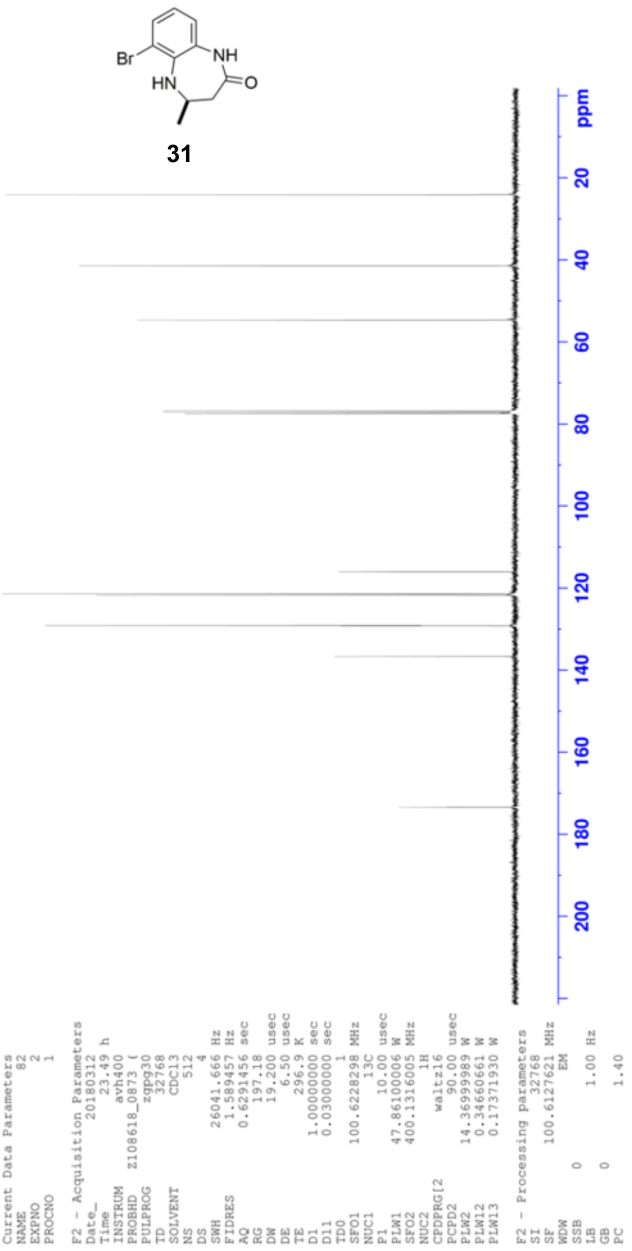

^{1}H NMR 7-Methoxy-1-(pent-4-yn-1-yl)-1,2,3,4-tetrahydroquinoline

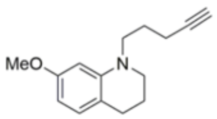

34

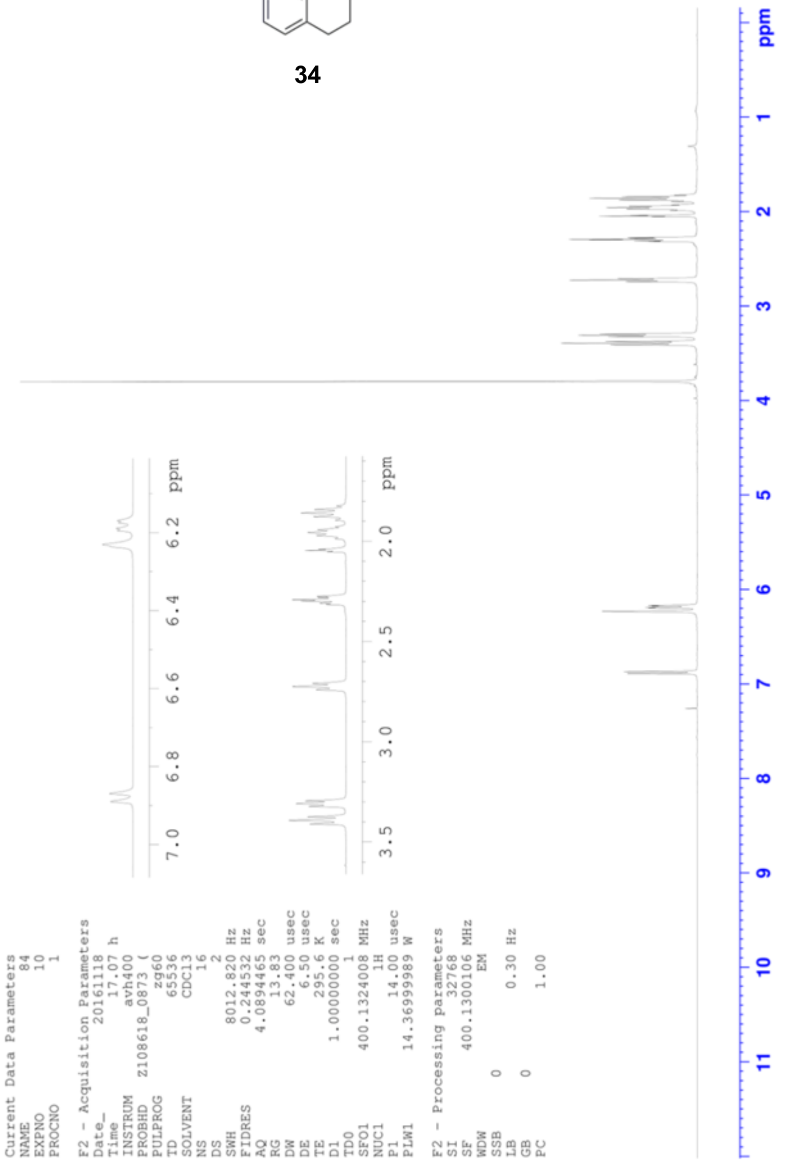

^{13}C NMR 7-Methoxy-1-(pent-4-yn-1-yl)-1,2,3,4-tetrahydroquinoline

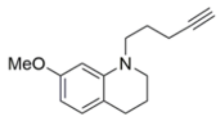

34

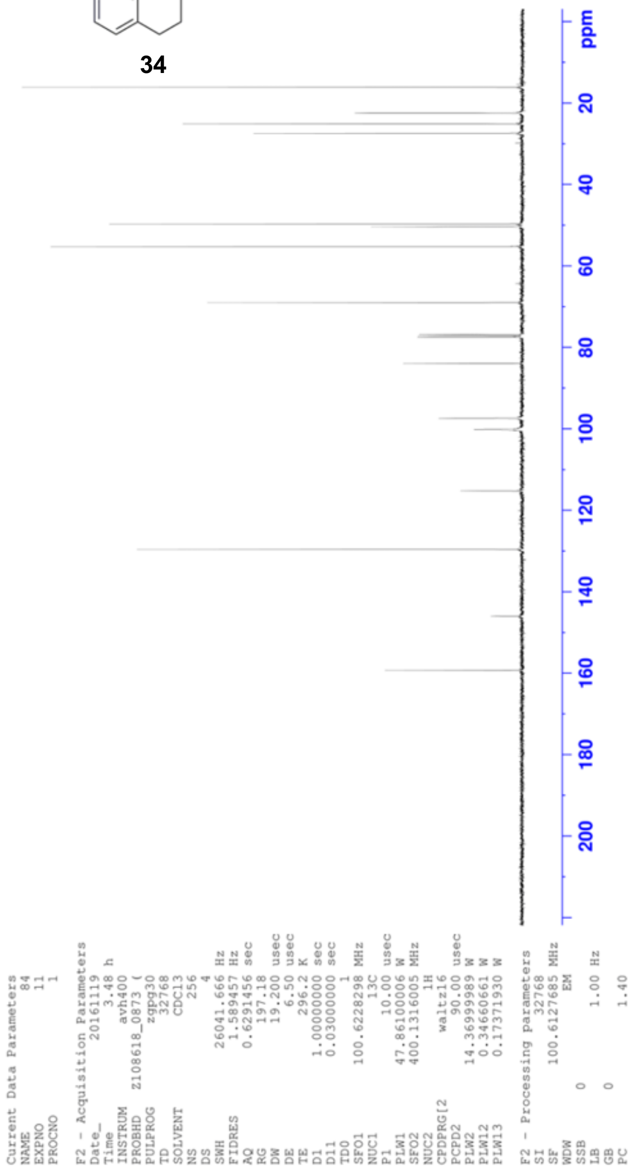

^{1}H NMR (*E*)-7-Methoxy-1-(5-(4,4,5,5-tetramethyl-1,3,2-dioxaborolan-2-yl)pent-4-en-1-yl)-1,2,3,4-tetrahydroquinoline

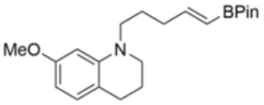

35

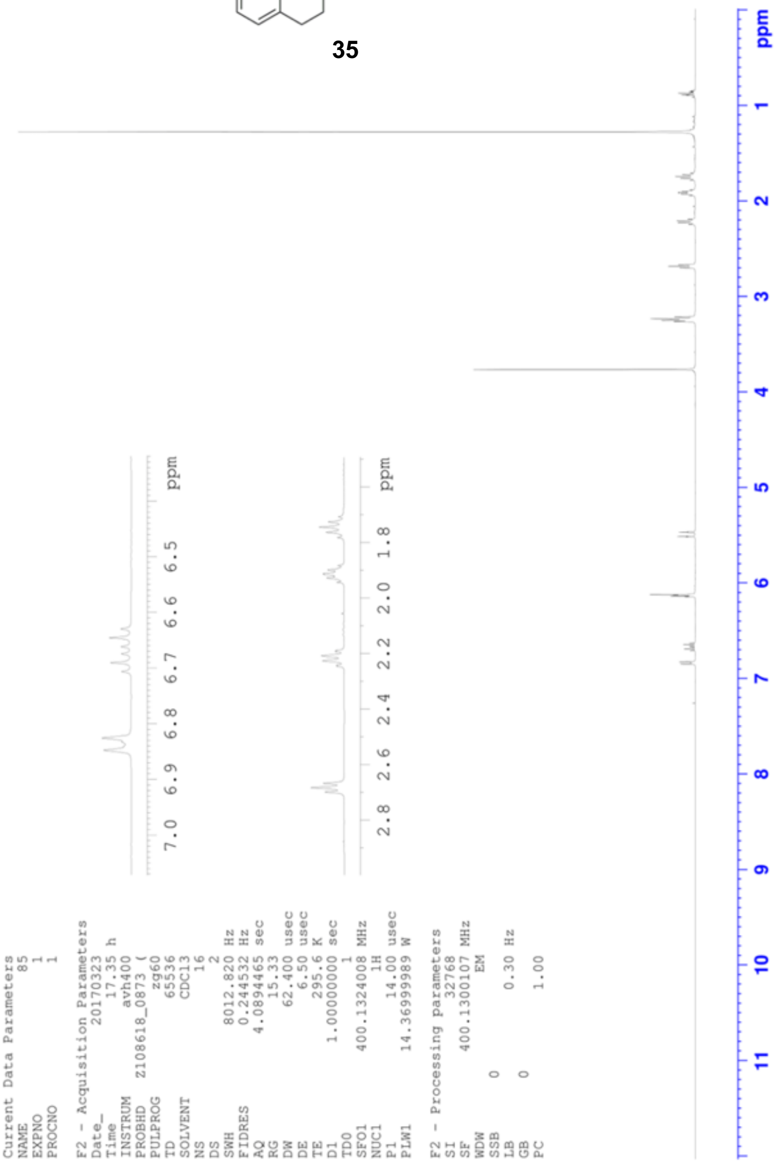

^{13}C NMR (*E*)-7-Methoxy-1-(5-(4,4,5,5-tetramethyl-1,3,2-dioxaborolan-2-yl)pent-4-en-1-yl)-1,2,3,4-tetrahydroquinoline

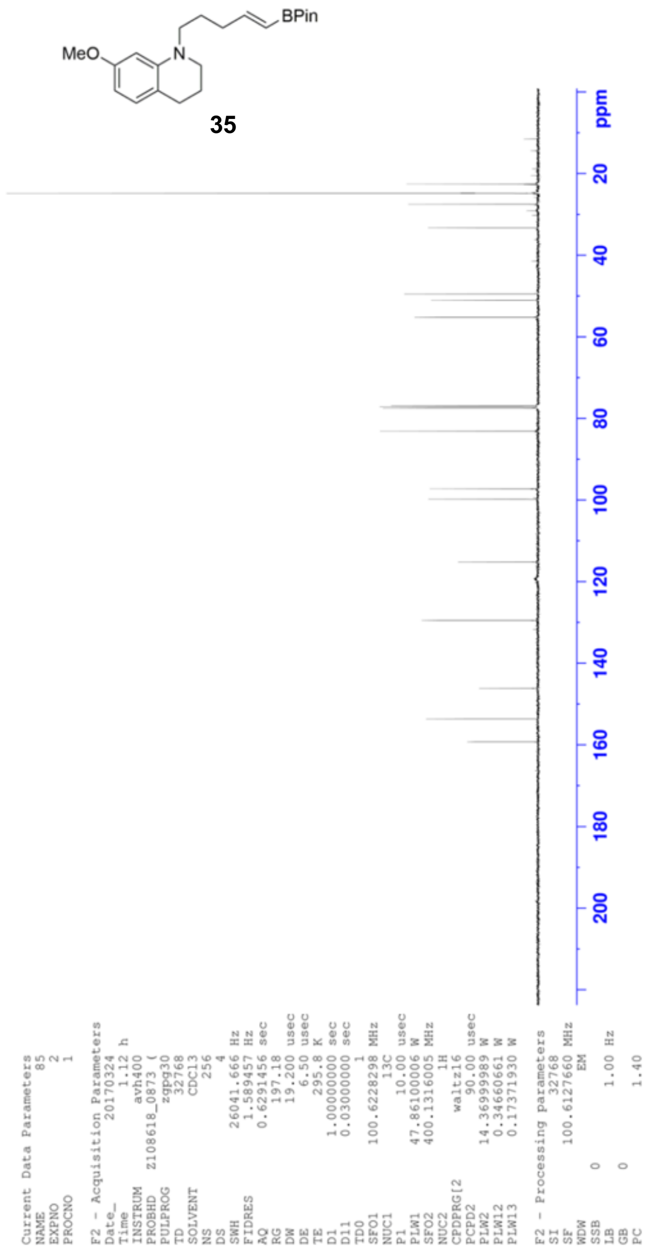

^{1}H NMR (*R*,*E*)-6-(5-(7-Methoxy-3,4-dihydroquinolin-1(2*H*)-yl)pent-1-en-1-yl)-4-methyl-1,3,4,5-tetrahydro-2*H*-benzo[*b*][1,4]diazepin-2-one

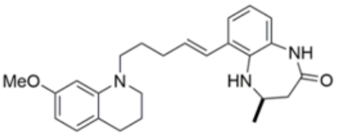

2

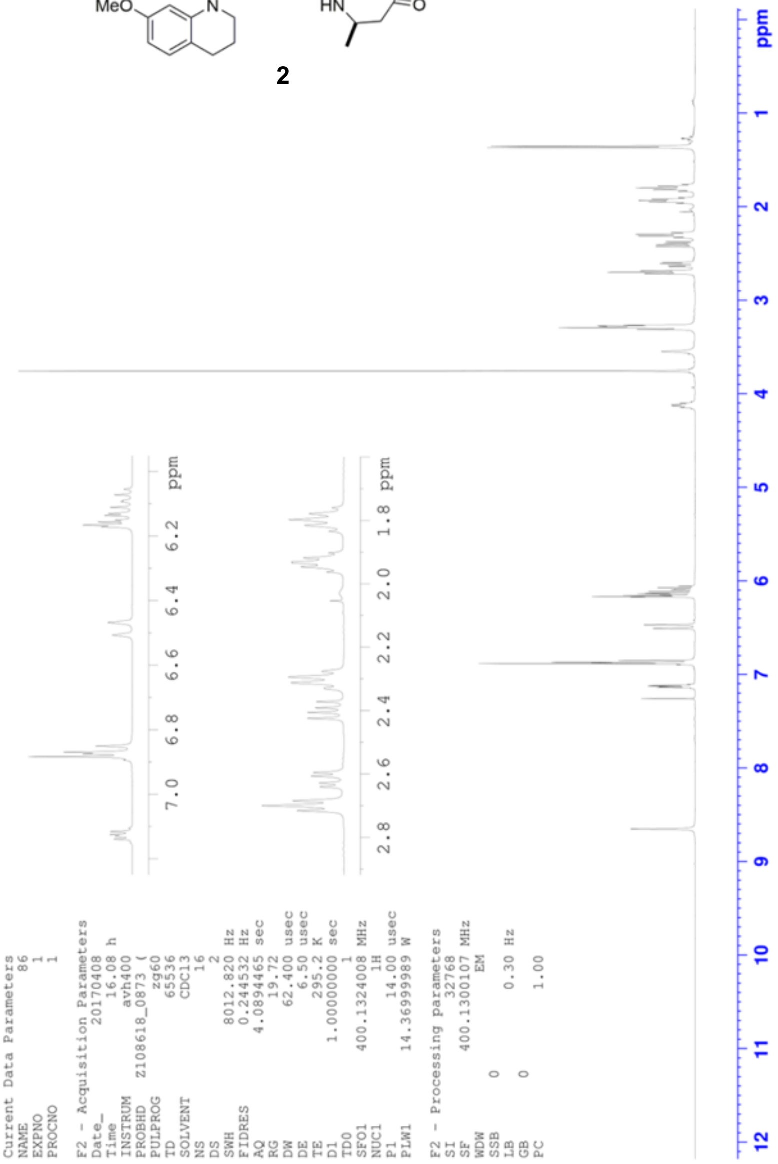

^{13}C NMR (*R,E*)-6-(5-(7-Methoxy-3,4-dihydroquinolin-1(2*H*)-yl)pent-1-en-1-yl)-4-methyl-1,3,4,5-tetrahydro-2*H*-benzo[*b*][1,4]diazepin-2-one

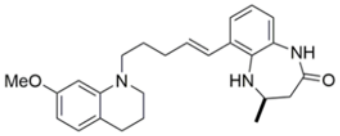

2

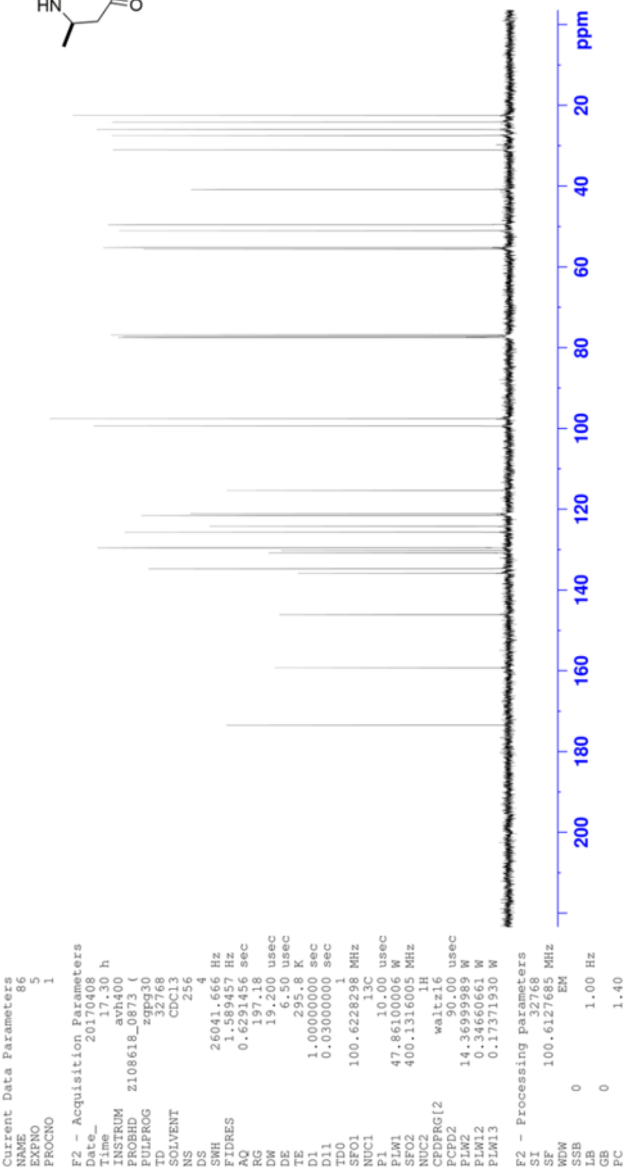

^{1}H NMR (*S,E*)-6-(5-(7-Methoxy-3,4-dihydroquinolin-1(2*H*)-yl)pent-1-en-1-yl)-4-methyl-1,3,4,5-tetrahydro-2*H*-benzo[*b*][1,4]diazepin-2-one

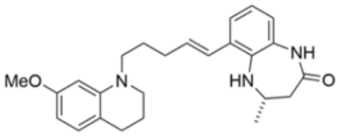

3

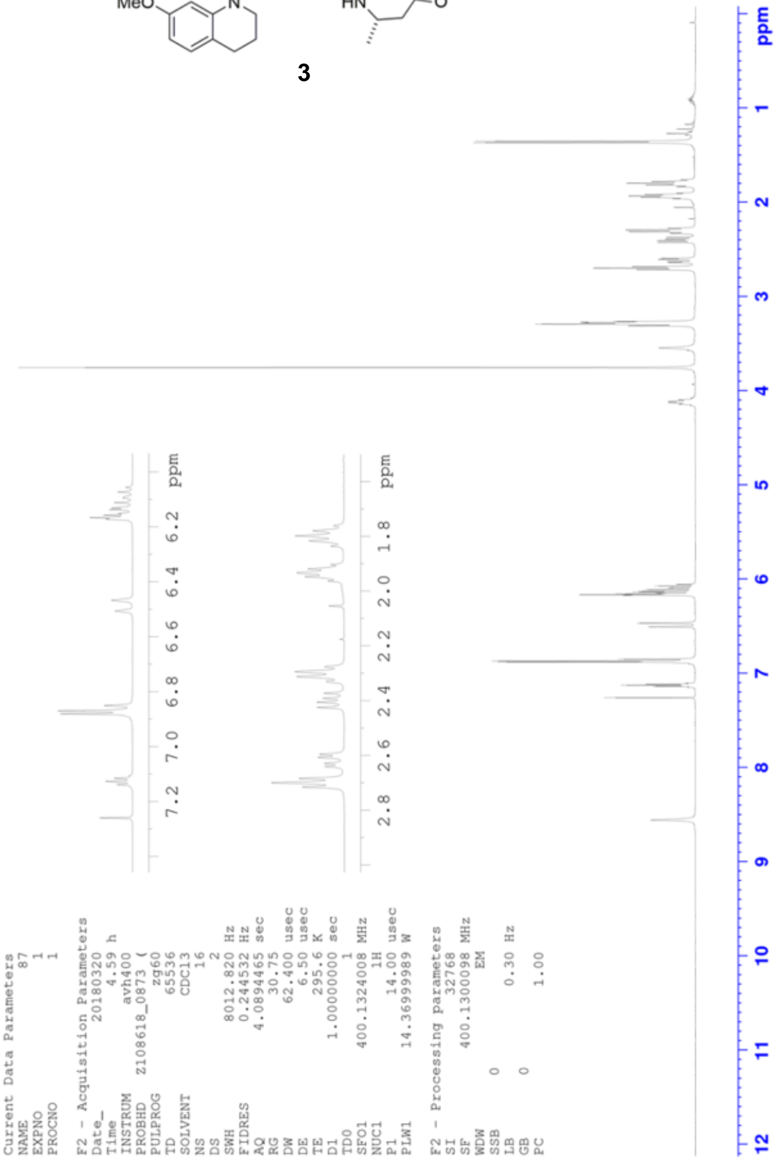

^{1}H NMR (*Z*)-7-Methoxy-1-(5-(triethylsilyl)pent-4-en-1-yl)-1,2,3,4-tetrahydroquinoline

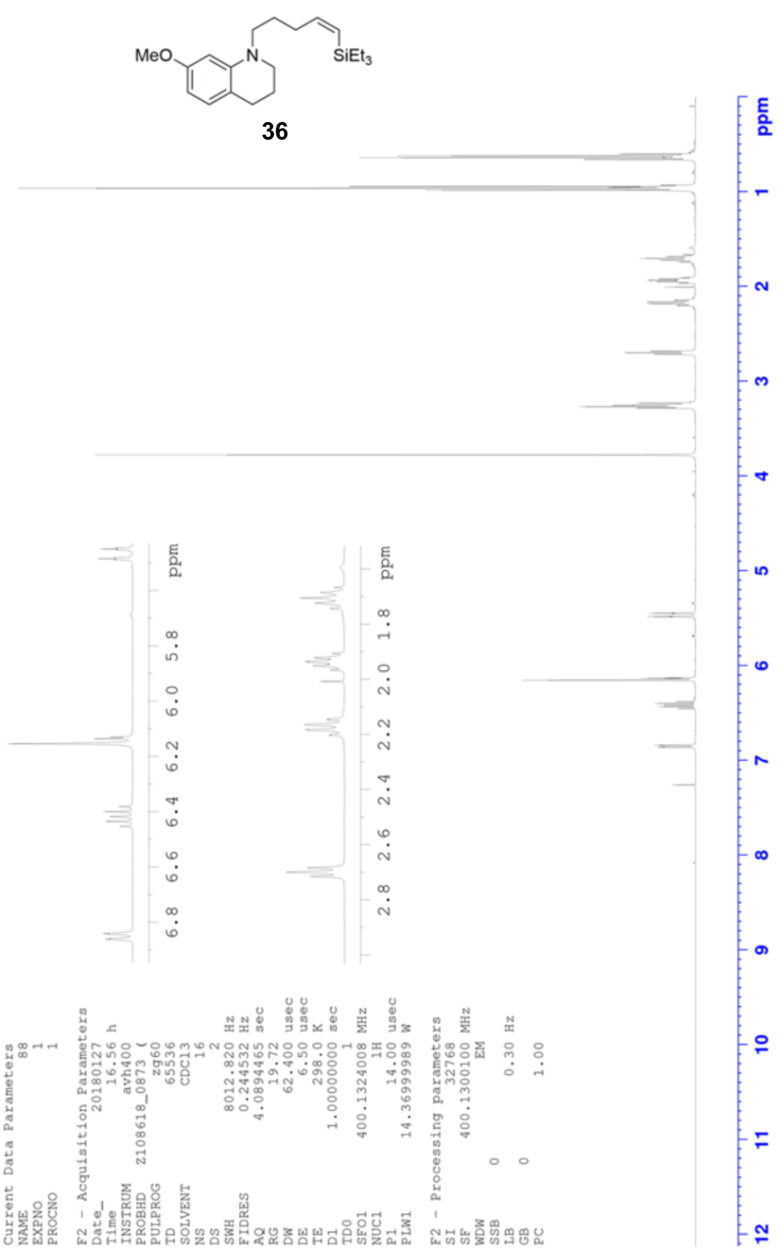

^{13}C NMR (*Z*)-7-Methoxy-1-(5-(triethylsilyl)pent-4-en-1-yl)-1,2,3,4-tetrahydroquinoline

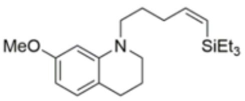

36

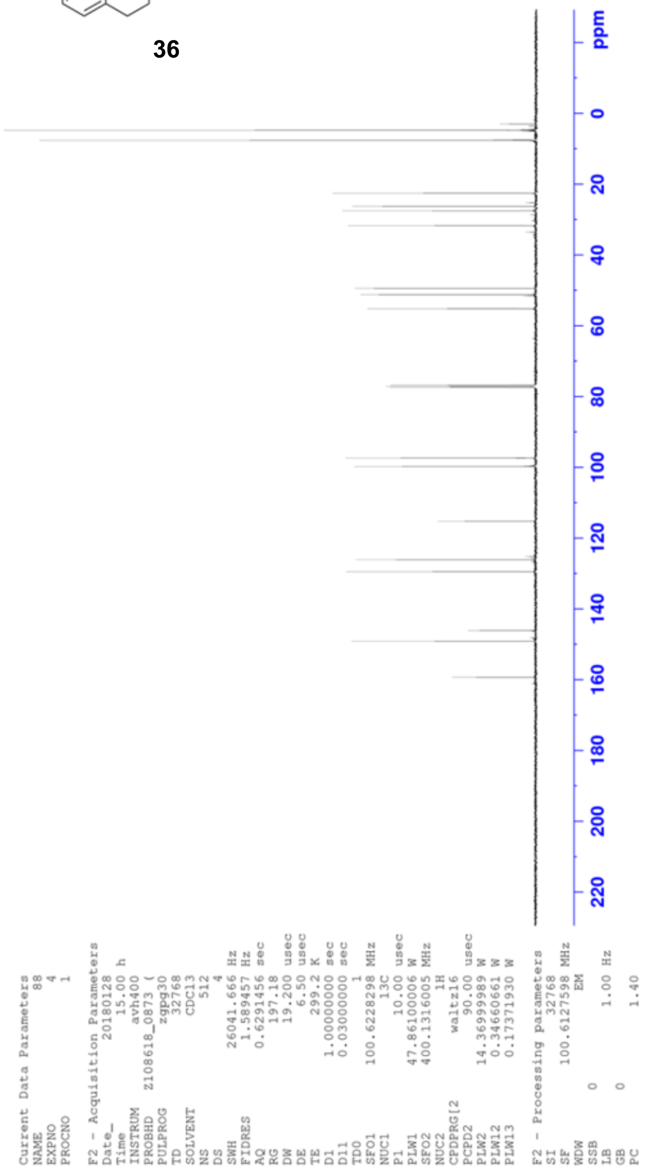

^{1}H NMR (*R,Z*)-6-(5-(7-Methoxy-3,4-dihydroquinolin-1(2*H*)-yl)pent-1-en-1-yl)-4-methyl-1,3,4,5-tetrahydro-2*H*-benzo[*b*][1,4]diazepin-2-one

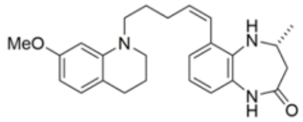

25

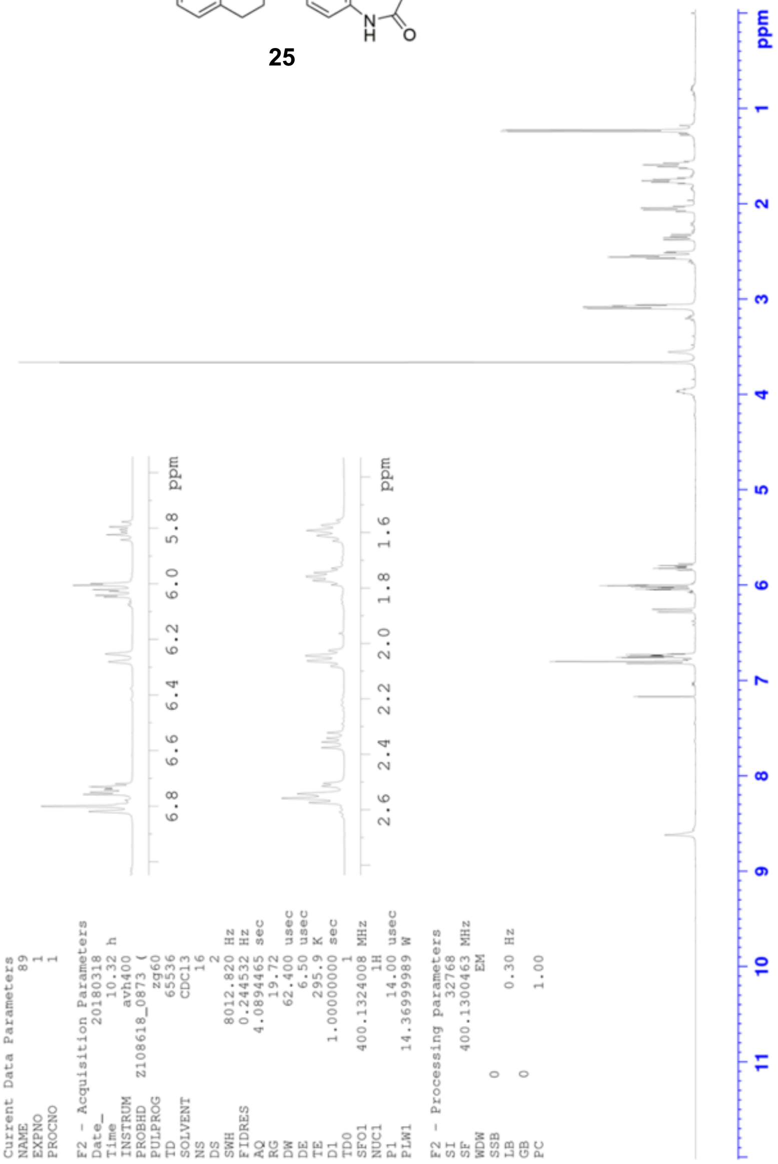

^{13}C NMR (*R*,*Z*)-6-(5-(7-Methoxy-3,4-dihydroquinolin-1(2*H*)-yl)pent-1-en-1-yl)-4-methyl-1,3,4,5-tetrahydro-2*H*-benzo[*b*][1,4]diazepin-2-one

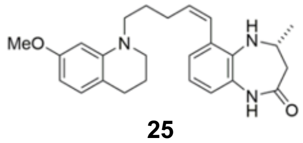

25

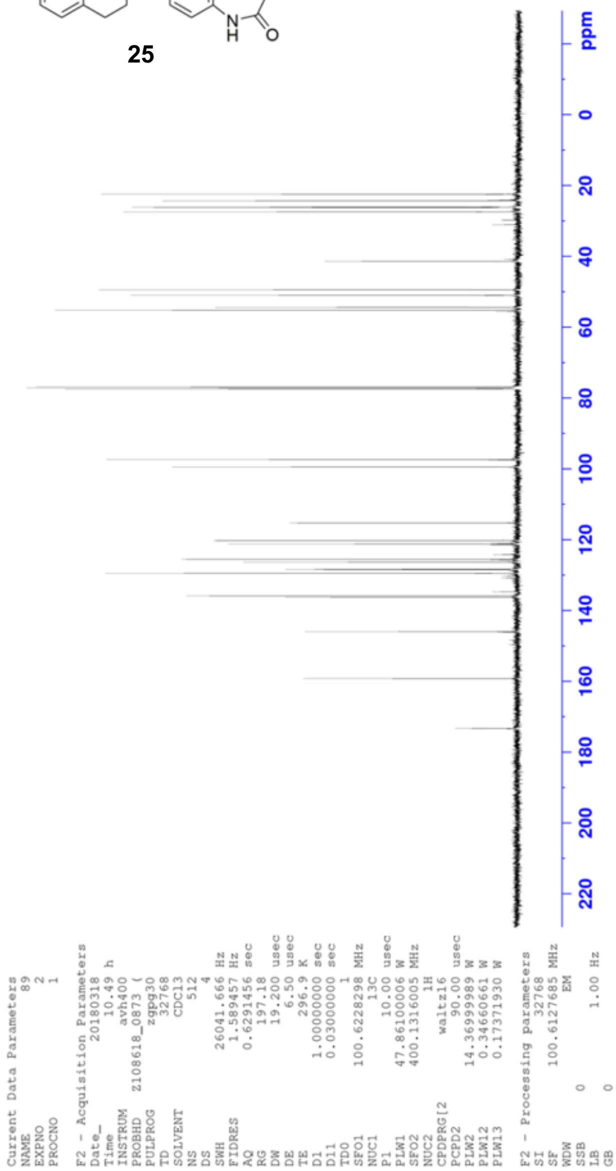

^{1}H NMR 1-(5-(2-Fluoro-3-nitrophenyl)pent-4-yn-1-yl)-7-methoxy-1,2,3,4-tetrahydroquinoline

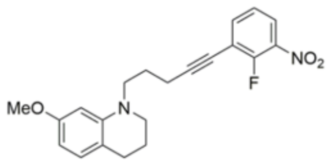

37

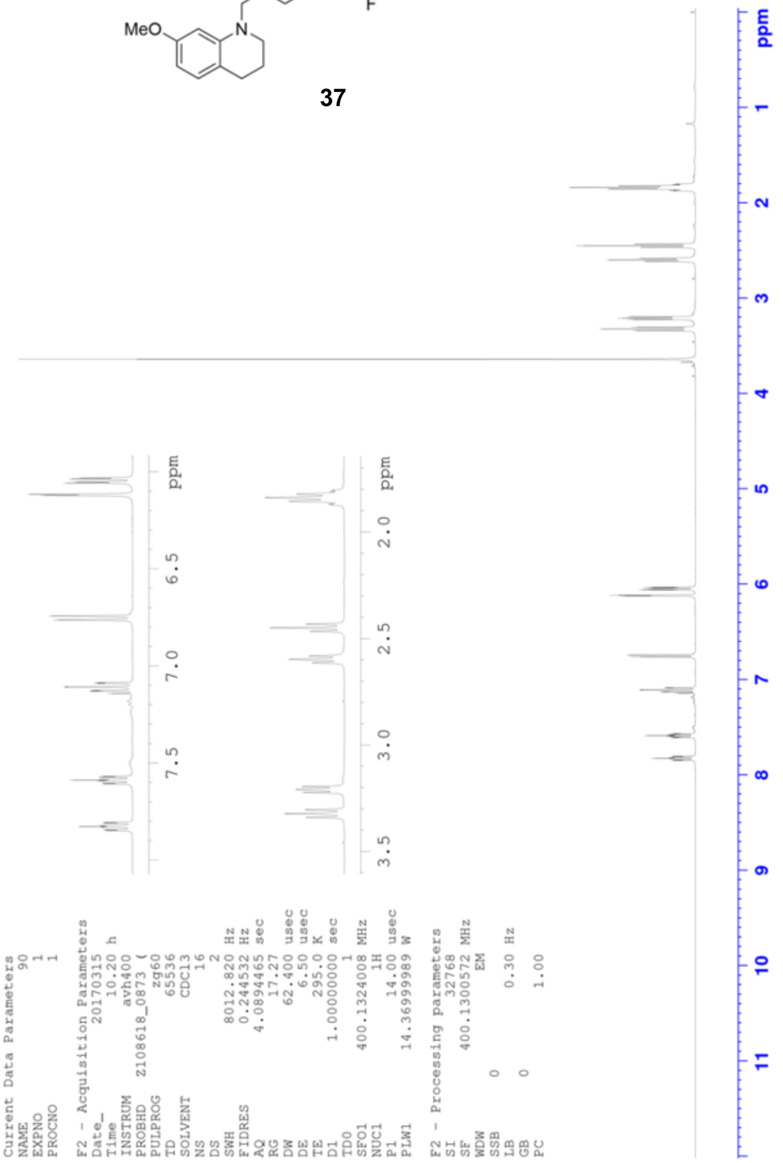

^{19}F NMR 1-(5-(2-Fluoro-3-nitrophenyl)pent-4-yn-1-yl)-7-methoxy-1,2,3,4-tetrahydroquinoline

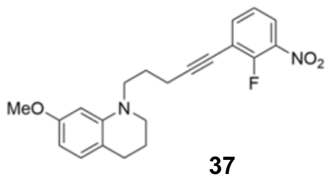

37

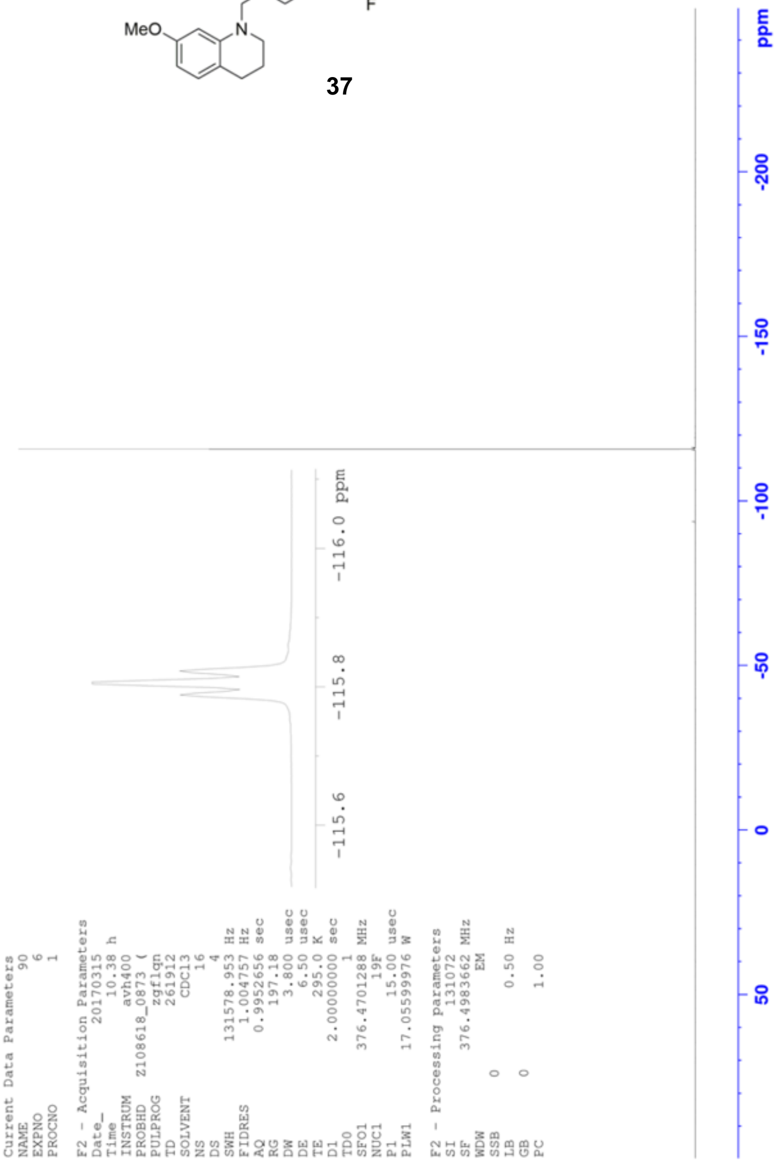

^{13}C NMR 1-(5-(2-Fluoro-3-nitrophenyl)pent-4-yn-1-yl)-7-methoxy-1,2,3,4-tetrahydroquinoline

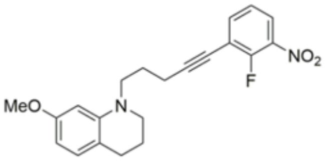

37

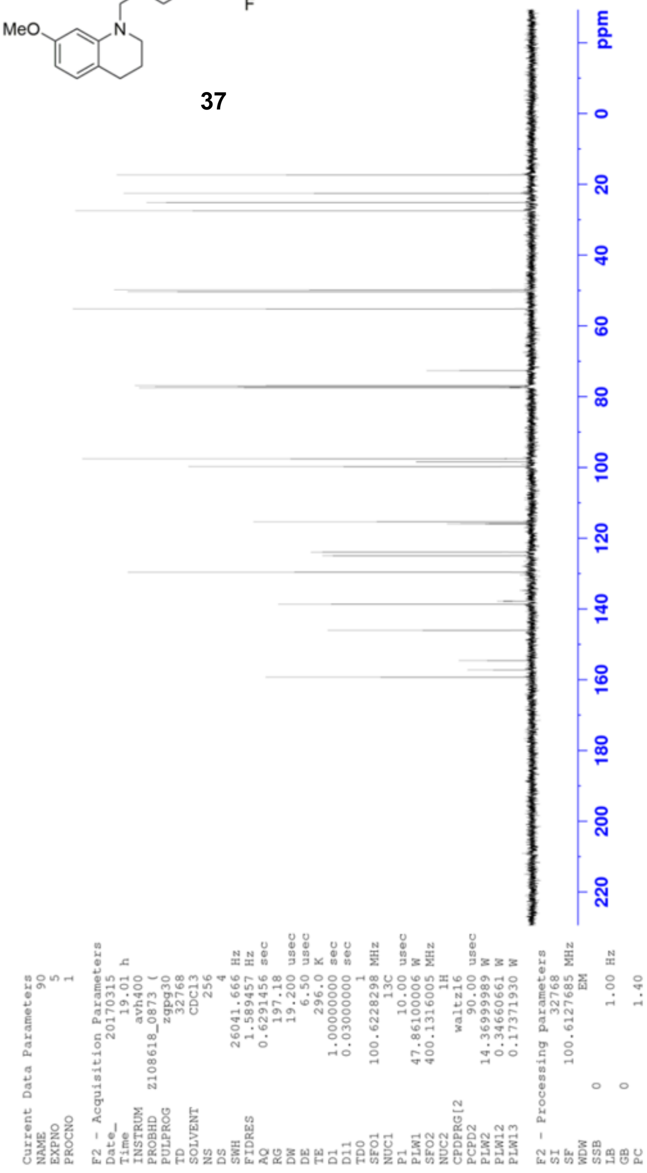

^{1}H NMR *tert*-Butyl (*S*)-3-((2-(5-(7-methoxy-3,4-dihydroquinolin-1(2*H*)-yl)pent-1-yn-1-yl)-6-nitrophenyl)amino)butanoate

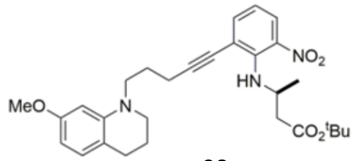

38

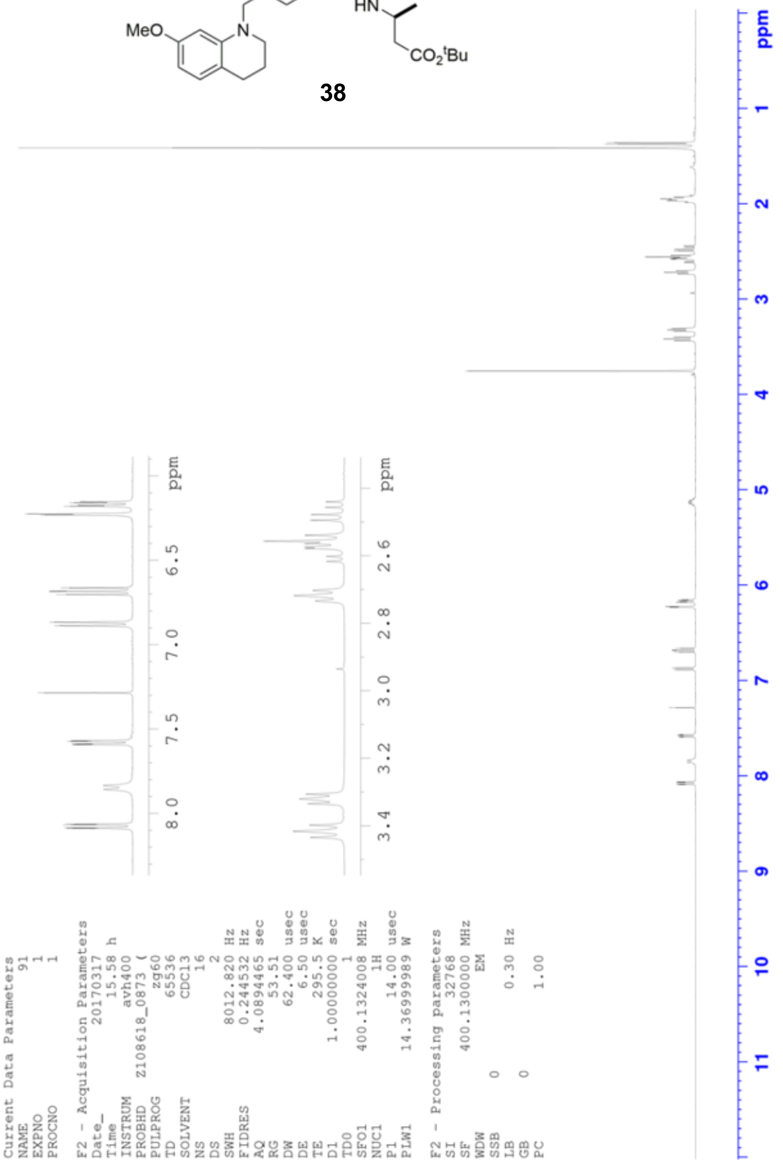

^{13}C NMR *tert*-Butyl (*S*)-3-((2-(5-(7-methoxy-3,4-dihydroquinolin-1(2*H*)-yl)pent-1-yn-1-yl)-6-nitrophenyl)amino)butanoate

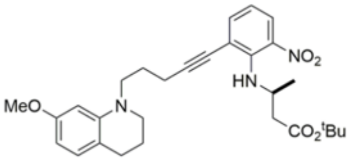

38

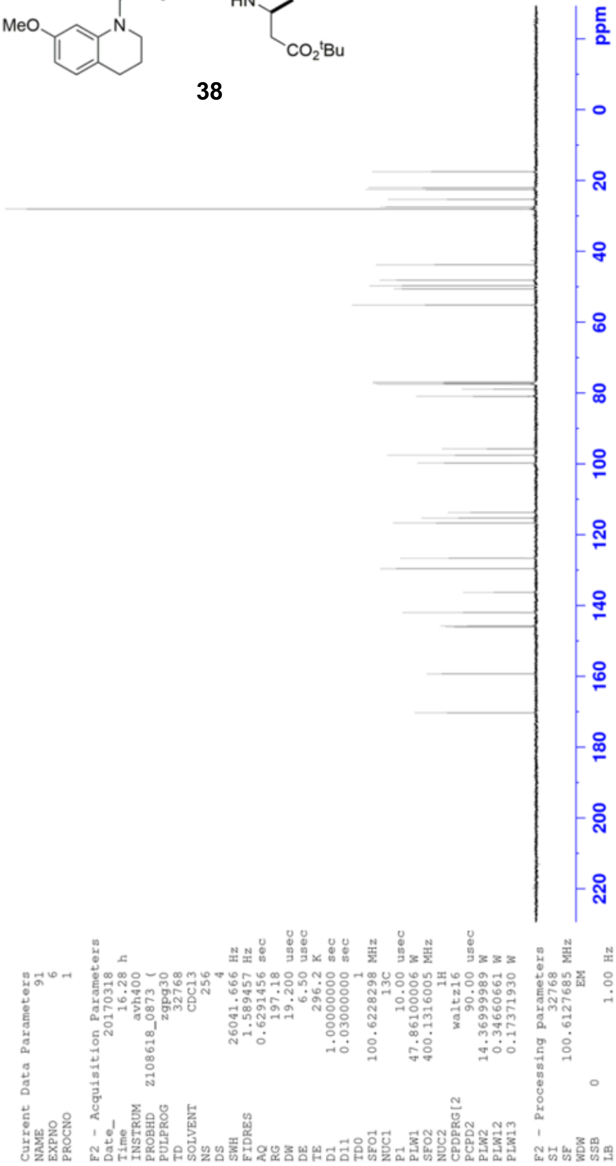

^{1}H NMR (*R*)-6-(5-(7-Methoxy-3,4-dihydroquinolin-1(2*H*)-yl)pent-1-yn-1-yl)-4-methyl-1,3,4,5-tetrahydro-2*H*-benzo[*b*][1,4]diazepin-2-one

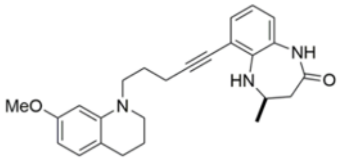

26

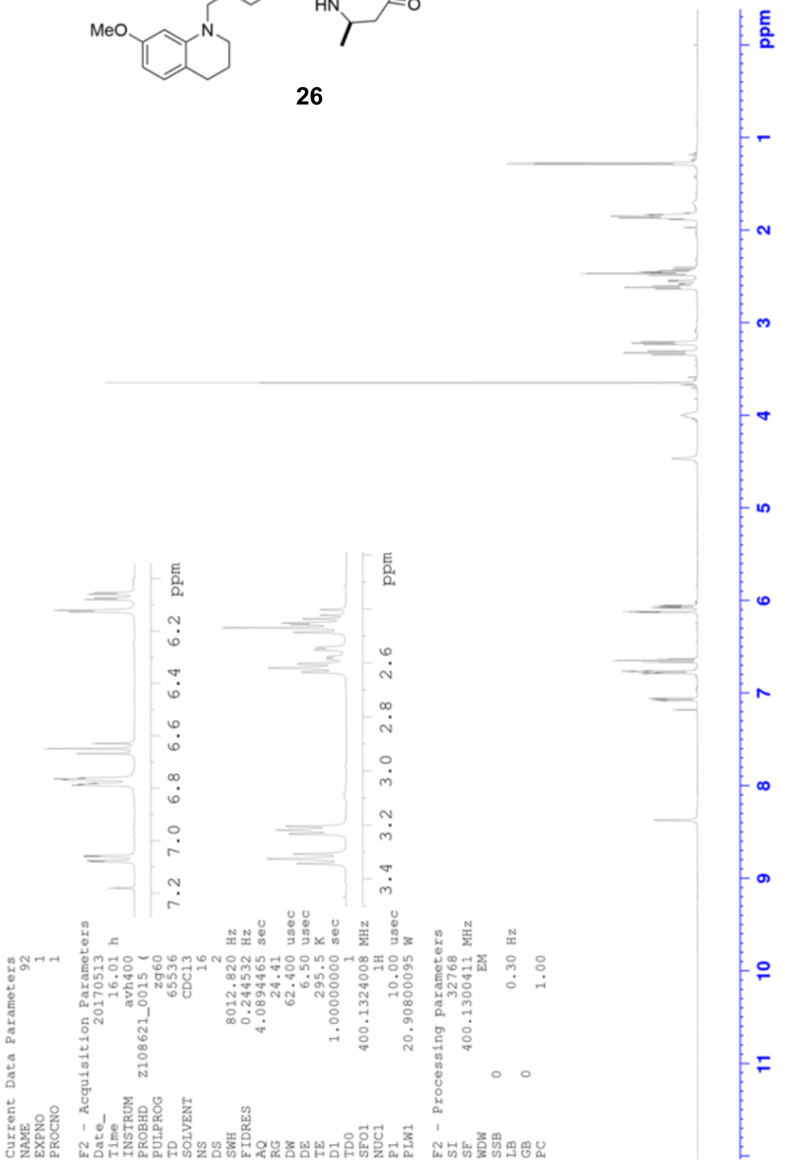

^{13}C NMR (*R*)-6-(5-(7-Methoxy-3,4-dihydroquinolin-1(2*H*)-yl)pent-1-yn-1-yl)-4-methyl-1,3,4,5-tetrahydro-2*H*-benzo[*b*][1,4]diazepin-2-one

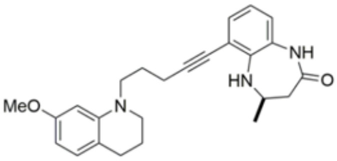

26

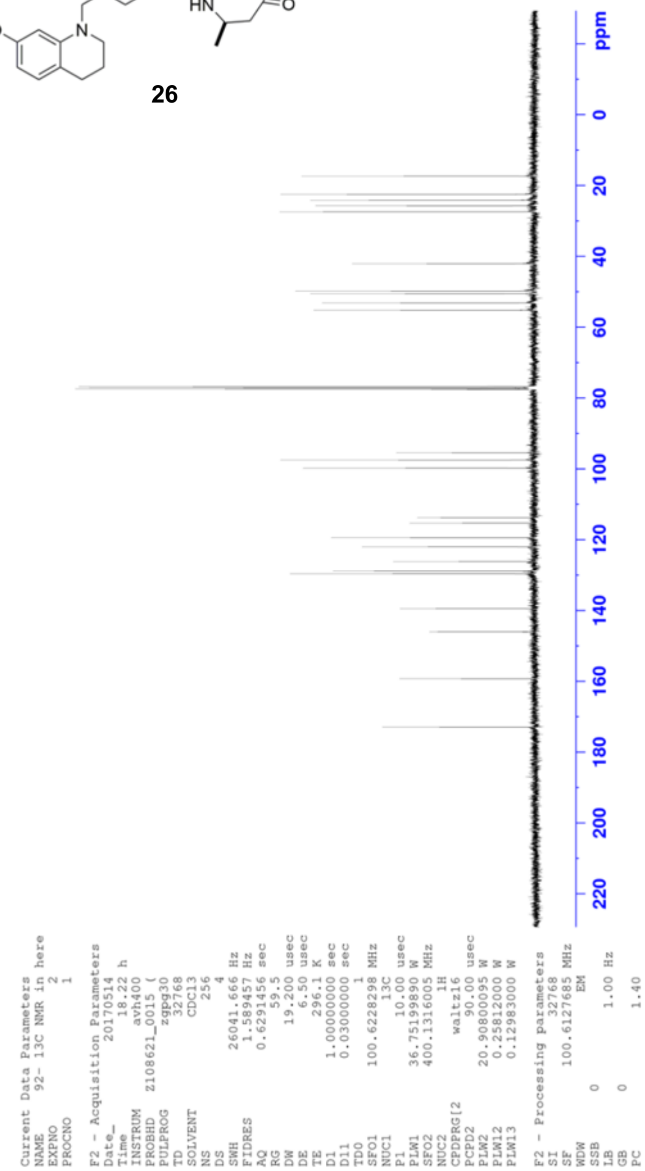

^{1}H NMR (*R*)-6-(5-(7-Methoxy-3,4-dihydroquinolin-1(2*H*)-yl)pentyl)-4-methyl-1,3,4,5-tetrahydro-2*H*-benzo[*b*][1,4]diazepin-2-one

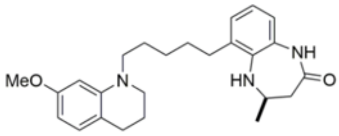

27

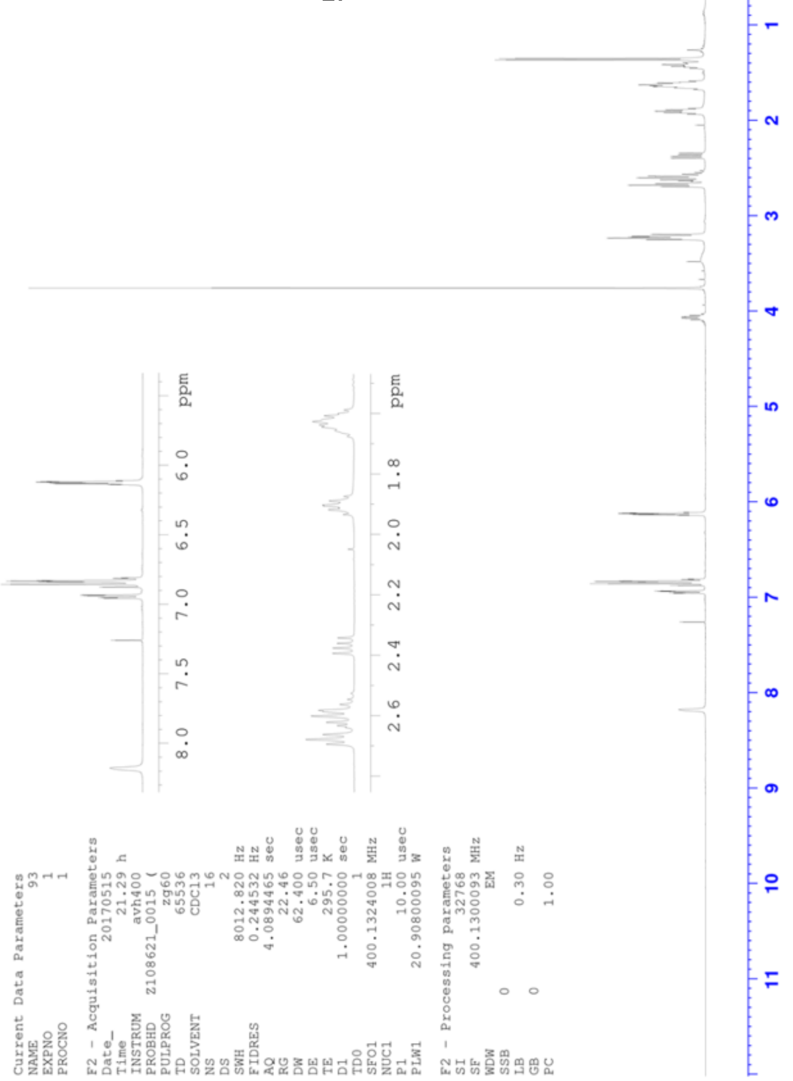

^{13}C NMR (*R*)-6-(5-(7-Methoxy-3,4-dihydroquinolin-1(2*H*)-yl)pentyl)-4-methyl-1,3,4,5-tetrahydro-2*H*-benzo[*b*][1,4]diazepin-2-one

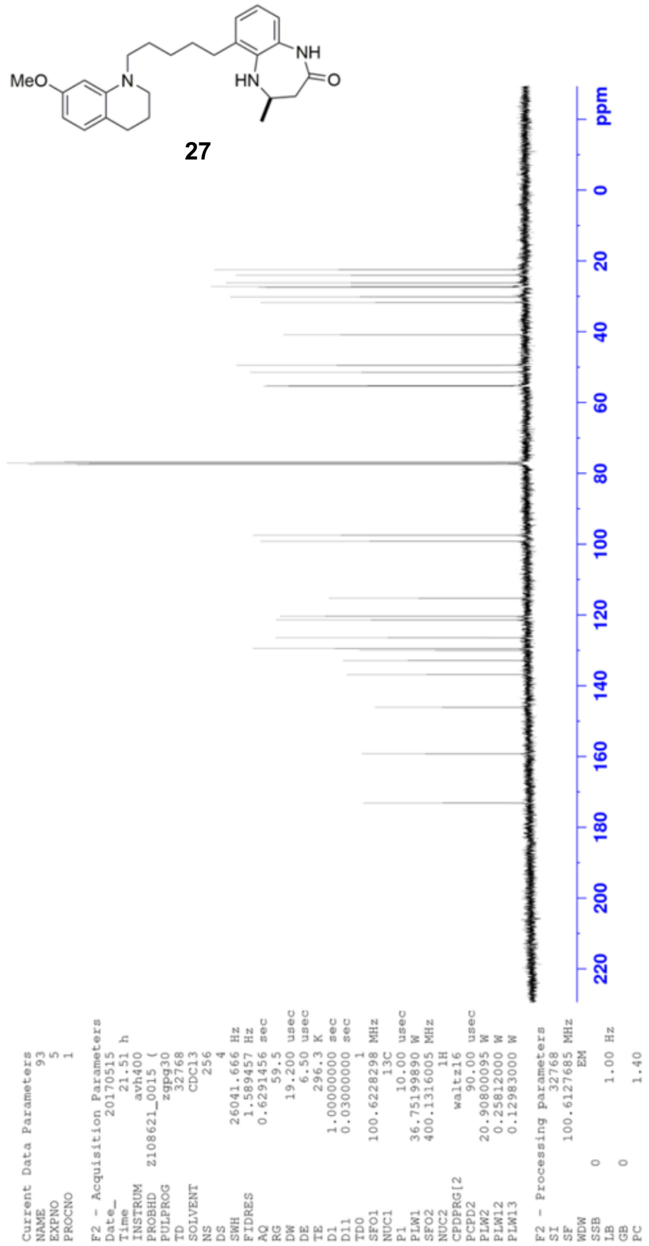

HPLC traces of biological tested compounds

02MM031-1 **Purity short run @254 nm** **5/5/2017 11:36 am**

Sample Name	02MM031-1
Vial Number	1
Injection Volume	10
Acquisition Date/Time	5/5/2017 11:15 am
Acquisition Method	Purity short run @254 nm
Processing Method	Purity short run @254 nm

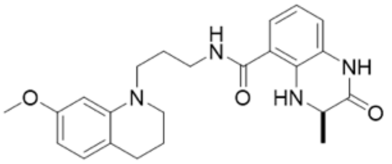

1

02MM031-1 : Injection 1

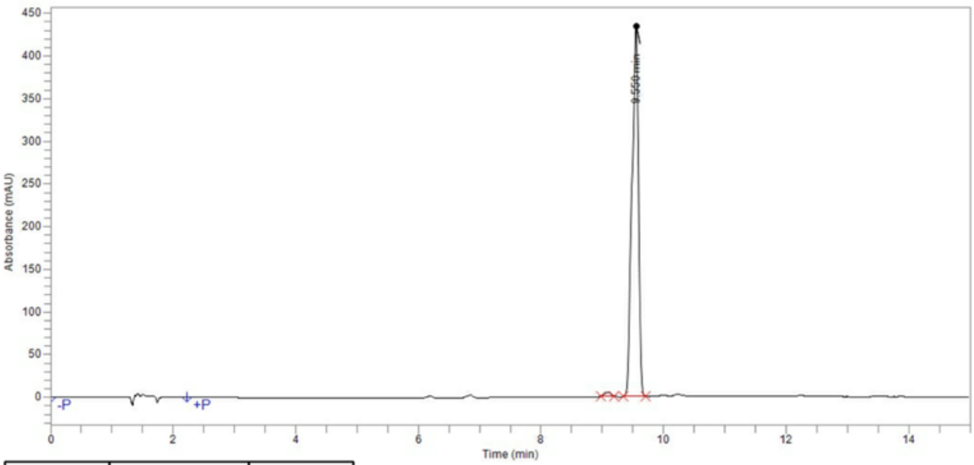

Time	Area	Area %
9.098	33,319.7	1.04
9.550	3,155,853.2	98.96
**Total**	3,189,172.9	100.00

5/5/2017 11:36 am **Flexar HPLC 2**

Sample Name	Michael's compound
Vial Number	1
Injection Volume	10
Acquisition Date/Time	5/4/2017 9:56 am
Acquisition Method	Purity short run @254 nm
Processing Method	Purity short run @254 nm

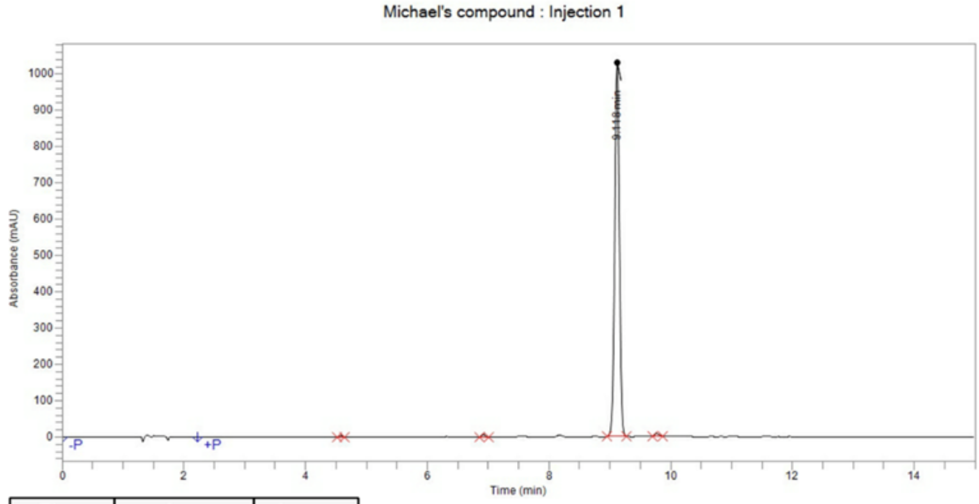

Time	Area	Area %
4.579	15,878.0	0.29
6.922	27,874.3	0.51
9.118	5,369,405.7	98.57
9.774	34,049.8	0.63
**Total**	5,447,207.8	100.00

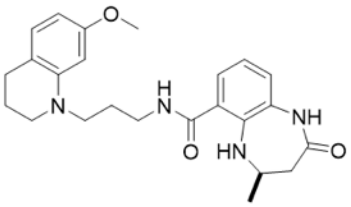

6

Acquisition Date/Time 12/15/2014 10:36 am Sample Name MIB219

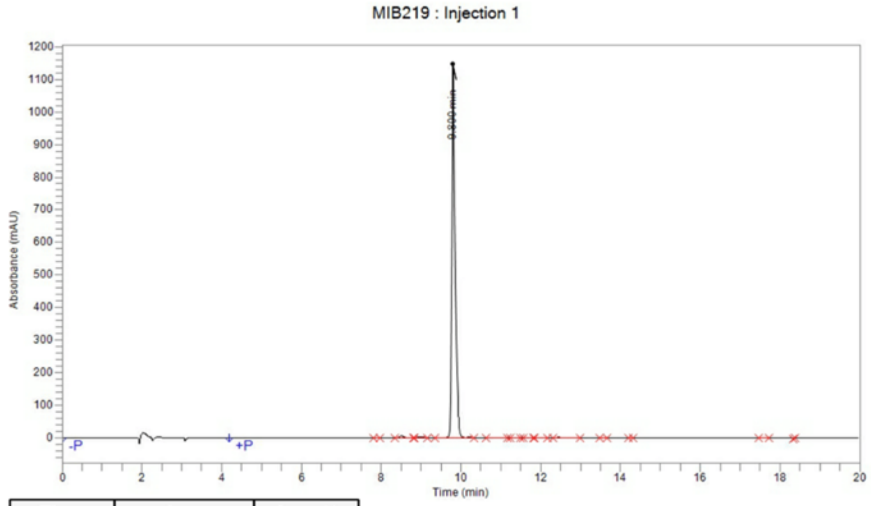

Time	Area	Area %
7.951	666	0.01
8.505	64,176	0.92
8.982	37,517	0.54
9.470	1,576	0.02
9.800	6,790,462	97.18
10.202	16,059	0.23
10.699	1,382	0.02
10.763	6,422	0.09
10.992	8,924	0.13
11.336	4,287	0.06
11.651	4,851	0.07
11.959	9,880	0.14
12.449	24,252	0.35
12.725	9,822	0.14
13.571	2,391	0.03
14.264	731	0.01
17.575	1,708	0.02
17.654	864	0.01
18.348	1,277	0.02
**Total**	6,987,246	100.00

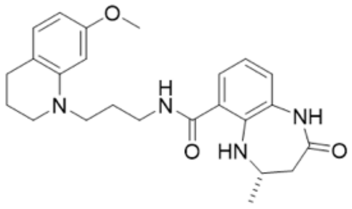

7

1/30/2015 3:56 pm

Acquisition Date/Time 11/3/2014 1:34 pm Sample Name MIB250_1

MIB250_1 : Injection 1

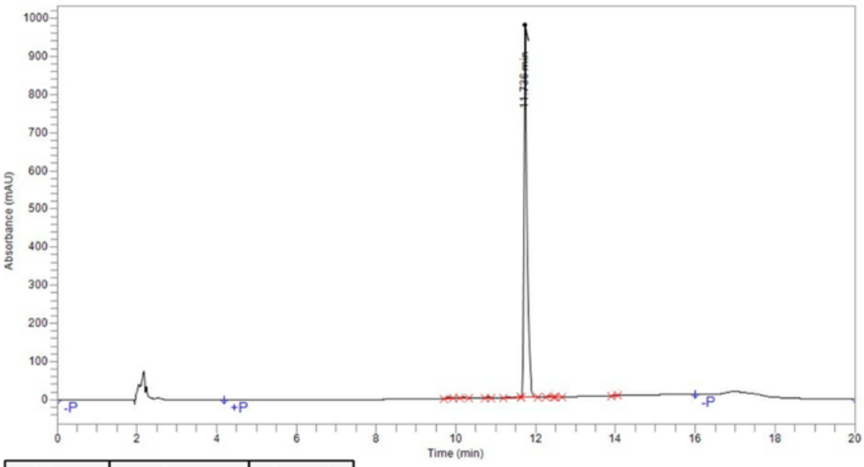

Time	Area	Area %
9.820	21,257	0.39
10.193	23,705	0.43
10.797	12,809	0.23
11.303	14,957	0.27
11.508	4,494	0.08
11.736	5,402,052	98.30
12.327	11,749	0.21
12.575	2,525	0.05
14.023	1,686	0.03
**Total**	5,495,234	100.00

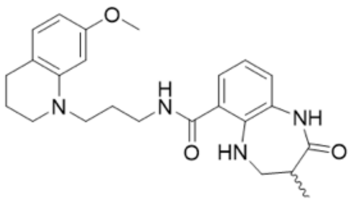

8

5/24/2016 8:32 am

Sample Name	JCI-C-045
Vial Number	1
Injection Volume	10
Acquisition Date/Time	4/10/2017 2:53 pm
Acquisition Method	Purity short run @254 nm
Processing Method	Purity short run @254 nm

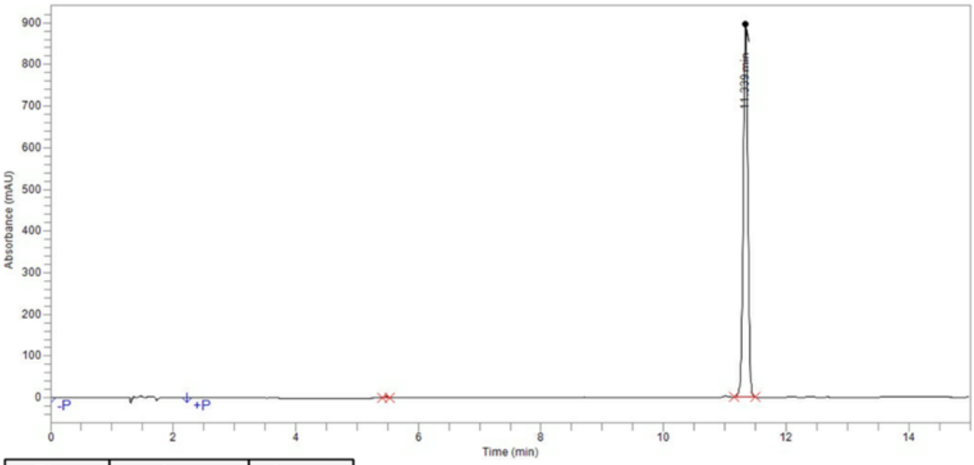

Time	Area	Area %
5.478	15,978.9	0.34
11.339	4,657,470.1	99.66
**Total**	4,673,449.0	100.00

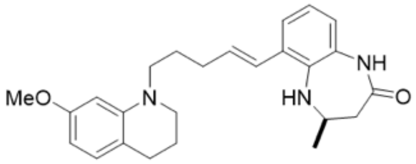

2

Acquisition Method	Purity short run @254 nm
Acquisition Date/Time	4/9/2018 1:26 pm
Injection Volume	10
Sample Name	JCI-D-27
Sample Description	
Batch Description	For start up

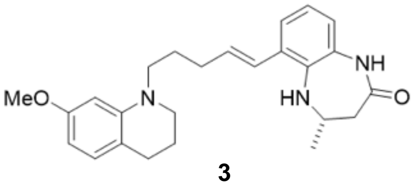

3

JCI-D-27 : Injection 1

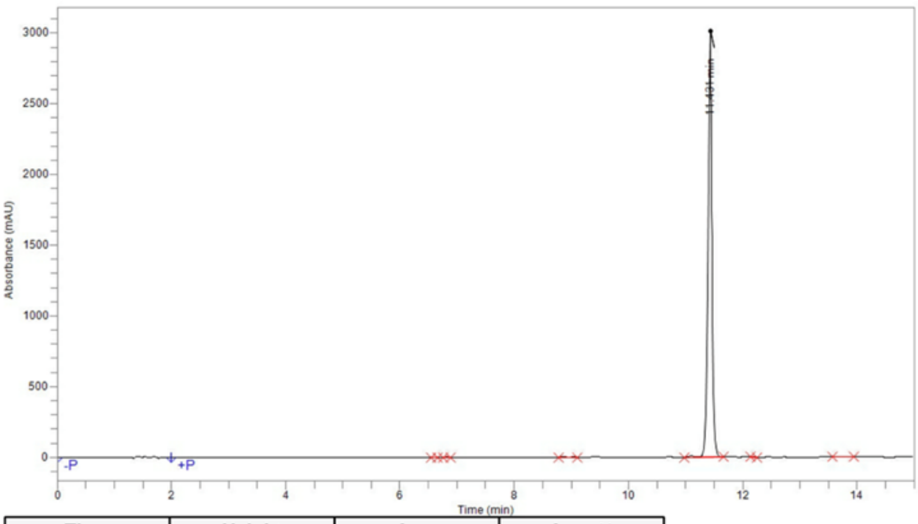

Time	Height	Area	Area %
6.602	1,007.7	3,142.8	0.02
6.826	2,359.6	8,344.5	0.06
8.858	5,646.4	23,426.5	0.16
9.039	1,145.2	5,134.6	0.03
11.101	9,539.5	62,711.0	0.42
11.431	2,967,697.5	14,637,888.4	98.98
12.182	1,127.7	4,137.0	0.03
13.736	4,227.7	43,387.4	0.29
**Total**		14,788,172.1	100.00

Acquisition Method	Purity short run @254 nm
Acquisition Date/Time	4/9/2018 4:24 pm
Injection Volume	10
Sample Name	JCI-D-24
Sample Description	
Batch Description	For start up

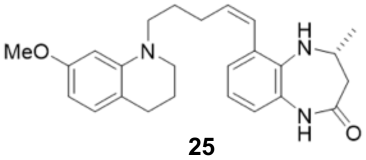

25

JCI-D-24 : Injection 1

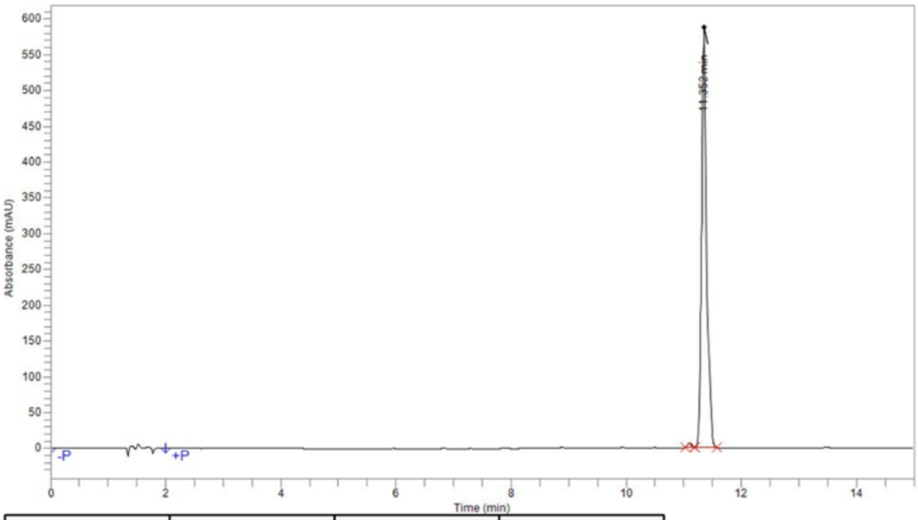

Time	Height	Area	Area %
11.097	6,027.5	26,959.5	0.76
11.352	588,244.1	3,535,796.4	99.24
**Total**		3,562,755.9	100.00

Acquisition Method	Purity short run @254 nm
Acquisition Date/Time	6/23/2017 12:50 pm
Injection Volume	10
Sample Name	JCLC-049 Alkyne
Sample Description	
Batch Description	For start up

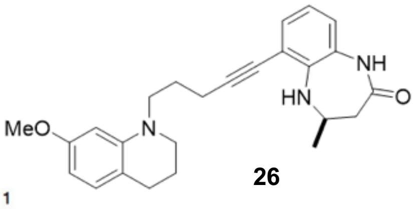

26

JCLC-049 Alkyne : Injection 1

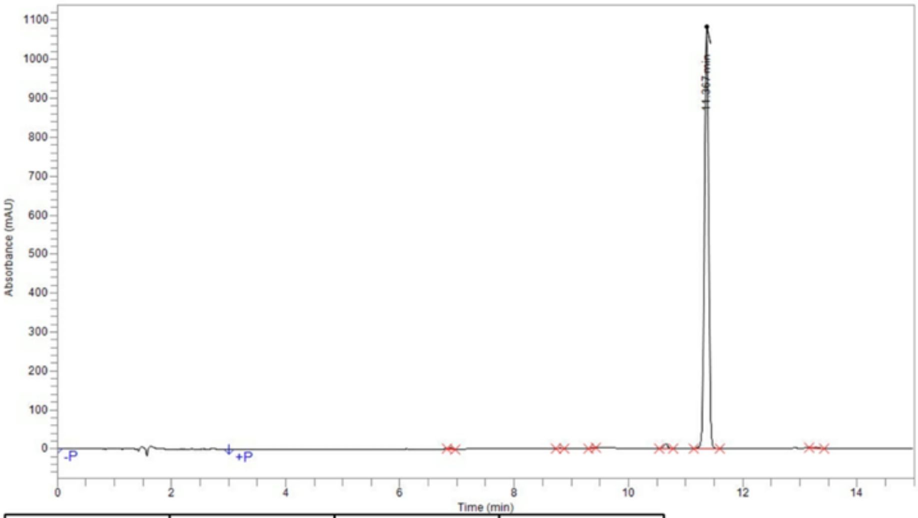

Time	Height	Area	Area %
6.890	3,828.5	12,425.4	0.24
8.809	1,866.7	6,543.7	0.13
9.373	1,461.5	5,207.6	0.10
10.653	12,085.5	58,136.2	1.11
11.367	1,084,261.4	5,129,105.3	98.05
13.280	2,723.0	19,533.4	0.37
**Total**		5,230,951.6	100.00

Sample Name	JCI-C-051 254
Vial Number	1
Injection Volume	10
Acquisition Date/Time	5/16/2017 11:35 am
Acquisition Method	Purity short run @254 nm
Processing Method	Purity short run @254 nm

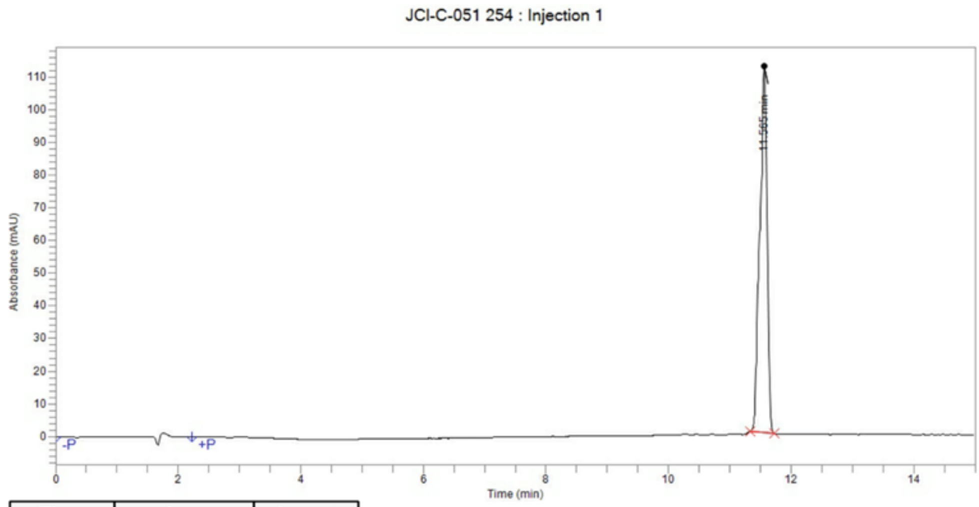

Time	Area	Area %
11.565	943,349.6	100.00
**Total**	943,349.6	100.00

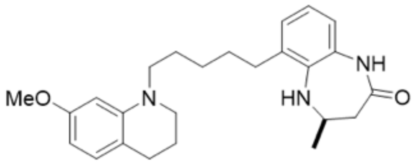

27

References

(1) Rooney, T. P. C.; Filippakopoulos, P.; Fedorov, O.; Picaud, S.; Cortopassi, W. A.; Hay, D. A.; Martin, S.; Tumber, A.; Rogers, C. M.; Philpott, M.; Wang, M.; Thompson, A. L.; Heightman, T. D.; Pryde, D. C.; Cook, A.; Paton, R. S.; Muller, S.; Knapp, S.; Brennan, P. E.; Conway, S. J. A Series of Potent CREBBP Bromodomain Ligands Reveals an Induced-Fit Pocket Stabilized by a Cation-π Interaction. *Angew. Chem. Int. Ed.* **2014**, *53*, 6126–6130.

(2) Hay, D. A.; Fedorov, O.; Martin, S.; Singleton, D. C.; Tallant, C.; Wells, C.; Picaud, S.; Philpott, M.; Monteiro, O. P.; Rogers, C. M.; Conway, S. J.; Rooney, T. P. C.; Tumber, A.; Yapp, C.; Filippakopoulos, P.; Bunnage, M. E.; Muller, S.; Knapp, S.; Schofield, C. J.; Brennan, P. E. Discovery and Optimization of Small-Molecule Ligands for the CBP/P300 Bromodomains. *J. Am. Chem. Soc.* **2014**, *136*, 9308–9319.

(3) Popp, T. A.; Tallant, C.; Rogers, C.; Fedorov, O.; Brennan, P. E.; Müller, S.; Knapp, S.; Bracher, F. Development of Selective CBP/P300 Benzoxazepine Bromodomain Inhibitors. *J. Med. Chem.* **2016**, *59*, 8889–8912.

(4) Unzue, A.; Xu, M.; Dong, J.; Wiedmer, L.; Spiliotopoulos, D.; Caflisch, A.; Nevado, C. Fragment-Based Design of Selective Nanomolar Ligands of the CREBBP Bromodomain. *J. Med. Chem.* **2016**, *59*, 1350–1356.

(5) Hügle, M.; Lucas, X.; Ostrovskyi, D.; Regenass, P.; Gerhardt, S.; Einsle, O.; Hau, M.; Jung, M.; Breit, B.; Günther, S.; Wohlwend, D. Beyond the BET Family: Targeting CBP/P300 with 4-Acyl Pyrroles. *Angew. Chem. Int. Ed.* **2017**, *56*, 12476–12480.

(6) Taylor, A. M.; Côté, A.; Hewitt, M. C.; Pastor, R.; Leblanc, Y.; Nasveschuk, C. G.; Romero, F. A.; Crawford, T. D.; Cantone, N.; Jayaram, H.; Setser, J.; Murray, J.; Beresini, M. H.; de Leon Boenig, G.; Chen, Z.; Conery, A. R.; Cummings, R. T.; Dakin, L. A.; Flynn, E. M.; Huang, O. W.; Kaufman, S.; Keller, P. J.; Kiefer, J. R.; Lai, T.; Li, Y.; Liao, J.; Liu, W.; Lu, H.; Pardo, E.; Tsui, V.; Wang, J.; Wang, Y.; Xu, Z.; Yan, F.; Yu, D.; Zawadzke, L.; Zhu, X.; Zhu, X.; Sims, R. J.; Cochran, A. G.; Bellon, S.; Audia, J. E.; Magnuson, S.; Albrecht, B. K. Fragment-Based Discovery of a Selective and Cell-Active Benzodiazepinone CBP/EP300 Bromodomain Inhibitor (CPI-637). *ACS Med. Chem. Lett.* **2016**, *7*, 531–536.

(7) Crawford, T. D.; Romero, F. A.; Lai, K. W.; Tsui, V.; Taylor, A. M.; de Leon Boenig, G.; Noland, C. L.; Murray, J.; Ly, J.; Choo, E. F.; Hunsaker, T. L.; Chan, E. W.; Merchant, M.; Kharbanda, S.; Gascoigne, K. E.; Kaufman, S.; Beresini, M. H.; Liao, J.; Liu, W.; Chen, K. X.; Chen, Z.; Conery, A. R.; Côté, A.; Jayaram, H.; Jiang, Y.; Kiefer, J. R.; Kleinheinz, T.; Li, Y.; Maher, J.; Pardo, E.; Poy, F.; Spillane, K. L.; Wang, F.; Wang, J.; Wei, X.; Xu, Z.; Xu, Z.; Yen, I.; Zawadzke, L.; Zhu, X.; Bellon, S.; Cummings, R.; Cochran, A. G.; Albrecht, B. K.; Magnuson, S. Discovery of a Potent and Selective in Vivo Probe (GNE-272) for the Bromodomains of CBP/EP300. *J. Med. Chem.* **2016**, *59*, 10549–10563.

(8) Romero, F. A.; Murray, J.; Lai, K. W.; Tsui, V.; Albrecht, B. K.; An, L.; Beresini, M. H.; de Leon Boenig, G.; Bronner, S. M.; Chan, E. W.; Chen, K. X.; Chen, Z.; Choo, E. F.; Clagg, K.; Clark, K.; Crawford, T. D.; Cyr, P.; de Almeida Nagata, D.; Gascoigne, K. E.; Grogan, J. L.; Hatzivassiliou, G.; Huang, W.; Hunsaker, T. L.; Kaufman, S.; Koenig, S. G.; Li, R.; Li, Y.; Liang, X.; Liao, J.; Liu, W.; Ly, J.; Maher, J.; Masui, C.; Merchant, M.; Ran, Y.; Taylor, A. M.; Wai, J.; Wang, F.; Wei, X.; Yu, D.; Zhu, B.-Y.; Zhu, X.; Magnuson, S. GNE-781, A Highly Advanced Potent and Selective Bromodomain Inhibitor of Cyclic Adenosine Monophosphate Response Element Binding Protein, Binding Protein (CBP). *J. Med. Chem.* **2017**, *60*, 9162–9183.

(9) Xiang, Q.; Wang, C.; Zhang, Y.; Xue, X.; Song, M.; Zhang, C.; Li, C.; Wu, C.; Li, K.; Hui, X.; Zhou, Y.; Smaill, J. B.; Patterson, A. V; Wu, D.; Ding, K.; Xu, Y. Discovery and Optimization of 1-(1H-Indol-1-Yl)Ethanone Derivatives as CBP/EP300 Bromodomain Inhibitors for the Treatment of Castration-Resistant Prostate Cancer. *Eur. J. Med. Chem.* **2018**, *147*, 238–252.

(10) Jennings, L. E.; Schiedel, M.; Hewings, D. S.; Picaud, S.; Laurin, C. M. C.; Bruno, P. A.; Bluck, J. P.; Scorah, A. R.; See, L.; Reynolds, J. K.; Moroglu, M.; Mistry, I. N.; Hicks, A.; Guzanov, P.; Clayton, J.; Evans, C. N. G.; Stazi, G.; Biggin, P. C.; Mapp, A. K.; Hammond, E. M.; Humphreys, P. G.; Filippakopoulos, P.; Conway, S. J. BET Bromodomain Ligands: Probing the WPF Shelf to Improve BRD4 Bromodomain Affinity and Metabolic Stability. *Bioorg. Med. Chem.* **2018**, *26*, 2937–2957.

(11) Davies, S. G.; Mulvaney, A. W.; Russell, A. J.; Smith, A. D. Parallel Synthesis of Homochiral β-Amino Acids. *Tetrahedron: Asymmetry* **2007**, *18*, 1554–1566.

(12) Srebnik, S. P. M. Hydroboration of Alkynes with Pinacolborane Catalyzed by HZrCp2Cl. *Organometallics* **1995**, *14*, 3127–3128.

(13) Aricó, C. S.; Cox, L. R. Regio- and Stereoselective Hydrosilylation of Terminal Alkynes Using Grubbs' First-Generation Olefin-Metathesis Catalyst. *Org. Biomol. Chem.* **2004**, *2*, 2558–2562.

(14) Hewings, D. S.; Wang, M.; Philpott, M.; Fedorov, O.; Uttarkar, S.; Filippakopoulos, P.; Picaud, S.; Vuppusetty, C.; Marsden, B.; Knapp, S.; Conway, S. J.; Heightman, T. D. 3,5-Dimethylisoxazoles Act As Acetyl-Lysine-Mimetic Bromodomain Ligands. *J. Med. Chem.* **2011**, *54*, 6761–6770.

(15) Kabsch, W. Automatic Indexing of Rotation Diffraction Patterns. *J. Appl. Cryst.* **1988**, *21*, 67–71.

(16) Kabsch, W. Evaluation of Single-Crystal X-Ray Diffraction Data from a Position-Sensitive Detector. *J. Appl. Cryst.* **1988**, *21*, 916–924.

(17) Kabsch, W. XDS. *Acta Crystallogr. D Biol. Crystallogr.* **2010**, *66*, 125–132.

(18) Evans, P. SCALA - Scale Together Multiple Observations of Reflections, 3.3.0. *MRC Laboratory of Molecular Biology, Cambridge* **2007**.

(19) McCoy, A. J.; Grosse-Kunstleve, R. W.; Storoni, L. C.; Read, R. J. Likelihood-Enhanced Fast Translation Functions. *Acta Crystallogr. D Biol. Crystallogr.* **2005**, *61*, 458–464.

(20) Filippakopoulos, P.; Picaud, S.; Mangos, M.; Keates, T.; Lambert, J.-P.; Barsyte-Lovejoy, D.; Felletar, I.; Volkmer, R.; Müller, S.; Pawson, T.; Gingras, A.-C.; Arrowsmith, C. H.; Knapp, S. Histone Recognition and Large-Scale Structural Analysis of the Human Bromodomain Family. *Cell* **2012**, *149*, 214–231.

(21) Perrakis, A.; Morris, R.; Lamzin, V. S. Automated Protein Model Building Combined with Iterative Structure Refinement. *Nat. Struct. Biol.* **1999**, *6*, 458–463.

(22) Emsley, P.; Cowtan, K. Coot: Model-Building Tools for Molecular Graphics. *Acta Crystallogr. D Biol. Crystallogr.* **2004**, *60*, 2126–2132.

(23) Murshudov, G. N.; Vagin, A. A.; Dodson, E. J. Refinement of Macromolecular Structures by the Maximum-Likelihood Method. *Acta Crystallogr. D Biol. Crystallogr.* **1997**, *53*, 240–255.

(24) Painter, J.; Merritt, E. A. Optimal Description of a Protein Structure in Terms of Multiple Groups Undergoing TLS Motion. *Acta Crystallogr. D Biol. Crystallogr.* **2006**, *62*, 439–450.

(25) Cosier, J.; Glazer, A. M. A Nitrogen-Gas-Stream Cryostat for General X-Ray Diffraction Studies. *J. Appl. Crystall.* **1986**, *19*, 105–107.

(26) Palatinus, L.; Chapuis, G. SUPERFLIP - a Computer Program for the Solution of Crystal Structures by Charge Flipping in Arbitrary Dimensions. *J. Appl. Crystall.* **2007**, *40*, 786–790.

(27) Cooper, R. I.; Thompson, A. L.; Watkin, D. J. CRYSTALS Enhancements: Dealing with Hydrogen Atoms in Refinement. *J. Appl. Crystall.* **2010**, *43*, 1100–1107.

(28) Parois, P.; Cooper, R. I.; Thompson, A. L. Crystal Structures of Increasingly Large Molecules: Meeting the Challenges with CRYSTALS Software. *Chemistry Central Journal* **2015**, *9*, 30.

(29) Gee, C. T.; Arntson, K. E.; Urick, A. K.; Mishra, N. K.; Hawk, L. M. L.; Wisniewski, A. J.; Pomerantz, W. C. K. Protein-Observed (19)F-NMR for Fragment Screening, Affinity Quantification and Druggability Assessment. *Nature Protocols* **2016**, *11*, 1414–1427.

(30) Erijman, A.; Dantes, A.; Bernheim, R.; Shifman, J. M.; Peleg, Y. Transfer-PCR (TPCR): A Highway for DNA Cloning and Protein Engineering. *J. Struct. Biol.* **2011**, *175*, 171–177.

(31) Cortopassi, W. A.; Kumar, K.; Paton, R. S. Cation-π Interactions in CREBBP Bromodomain Inhibition: An Electrostatic Model for Small-Molecule Binding Affinity and Selectivity. *Org. Biomol. Chem.* **2016**, *14*, 10926–10938.

(32) Vriend, G. WHAT IF: A Molecular Modeling and Drug Design Program. *Journal of Molecular Graphics* **1990**, *8*, 52–56.

(33) Lindorff-Larsen, K.; Piana, S.; Palmo, K.; Maragakis, P.; Klepeis, J. L.; Dror, R. O.; Shaw, D. E. Improved Side-Chain Torsion Potentials for the Amber Ff99SB Protein Force Field. *Proteins* **2010**, *78*, 1950–1958.

(34) Wang, J. M.; Wolf, R. M.; Caldwell, J. W.; Kollman, P. A.; Case, D. A. Development and Testing of a General Amber Force Field. *J. Comp. Chem.* **2004**, *25*, 1157–1174.

(35) Jorgensen, W. L.; Chandrasekhar, J.; Madura, J. D.; Impey, R. W.; Klein, M. L. Comparison of Simple Potential Functions for Simulating Liquid Water. *J. Chem. Phys.* **1983**, *79*, 926–935.

(36) Berendsen, H. J. C.; Postma, J. P. M.; van Gunsteren, W. F.; DiNola, A.; Haak, J. R. Molecular Dynamics with Coupling to an External Bath. *J. Chem. Phys.* **1984**, *81*, 3684–3690.

(37) Goga, N.; Rzepiela, A. J.; de Vries, A. H.; Marrink, S. J.; Berendsen, H. J. C. Efficient Algorithms for Langevin and DPD Dynamics. *J. Chem. Theory Comp.* **2012**, *8*, 3637–3649.

(38) Abraham, M. J.; Murtola, T.; Schulz, R.; Páll, S.; Smith, J. C.; Hess, B.; Lindahl, E. GROMACS: High Performance Molecular Simulations through Multi-Level Parallelism from Laptops to Supercomputers. *SoftwareX* **2015**, *1–2*, 19–25.

(39) Gowers, R. J.; Linke, M.; Barnoud, J.; Reddy, T. J. E.; Melo, M. N.; Seyler, S. L.; Domański, J.; Dotson, D. L.; Buchoux, S.; Kenney, I. M.; Beckstein, O. MDAnalysis: A Python Package for the Rapid Analysis of Molecular Dynamics Simulations. *Proceedings of the 15th Python in Science Conference*. SciPy 2016, pp 98–105.

(40) Morris, G. M.; Huey, R.; Lindstrom, W.; Sanner, M. F.; Belew, R. K.; Goodsell, D. S.; Olson, A. J. AutoDock4 and AutoDockTools4: Automated Docking with Selective Receptor Flexibility. *J. Comp. Chem.* **2009**, *30*, 2785–2791.

(41) Hewings, D. S.; Fedorov, O.; Filippakopoulos, P.; Martin, S.; Picaud, S.; Tumber, A.; Wells, C.; Olcina, M. M.; Freeman, K.; Gill, A.; Ritchie, A. J.; Sheppard, D. W.; Russell, A. J.; Hammond, E. M.; Knapp, S.; Brennan, P. E.; Conway, S. J. Optimization of 3,5-Dimethylisoxazole Derivatives as Potent Bromodomain Ligands. *J. Med. Chem.* **2013**, *56*, 3217–3227.
